# Isoliquiritigenin ameliorates abnormal oligodendrocyte development and behavior disorders induced by white matter injury

**DOI:** 10.3389/fphar.2024.1473019

**Published:** 2024-09-11

**Authors:** Dong Wu, Wenjuan Zhou, Jingyi Du, Tiantian Zhao, Naigang Li, Fan Peng, Anna Li, Xinyue Zhang, Meihua Zhang, Aijun Hao

**Affiliations:** ^1^ Key Laboratory of Maternal and Fetal Medicine of National Health Commission of China, Shandong Provincial Maternal and Child Health Care Hospital Affiliated to Qingdao University, Jinan, China; ^2^ Key Laboratory for Experimental Teratology of Ministry of Education, Shandong Key Laboratory of Mental Disorders, Department of Anatomy and Histoembryology, School of Basic Medical Sciences, Cheeloo College of Medicine, Shandong University, Jinan, China

**Keywords:** isoliquiritigenin, anxiety, depression, white matter injury, oligodendrocytes, microglia, HDAC3

## Abstract

**Background:**

White matter injury is a predominant form of brain injury in preterm infants. However, effective drugs for its treatment are currently lacking. Previous studies have shown the neuroprotective effects of Isoliquiritigenin (ISL), but its impact on white matter injury in preterm infants remains poorly understood.

**Aims:**

This study aimed to investigate the protective effects of ISL against white matter injury caused by infection in preterm infants using a mouse model of lipopolysaccharide-induced white matter injury, integrating network pharmacology as well as *in vivo* and *in vitro* experiments.

**Methods:**

This study explores the potential mechanisms of ISL on white matter injury by integrating network pharmacology. Core pathways and biological processes affected by ISL were verified through experiments, and motor coordination, anxiety-like, and depression-like behaviors of mice were evaluated using behavioral experiments. White matter injury was observed using hematoxylin-eosin staining, Luxol Fast Blue staining, and electron microscopy. The development of oligodendrocytes and the activation of microglia in mice were assessed by immunofluorescence. The expression of related proteins was detected by Western blot.

**Results:**

We constructed a drug-target network, including 336 targets associated with ISL treatment of white matter injury. The biological process of ISL treatment of white matter injury mainly involves microglial inflammation regulation and myelination. Our findings revealed that ISL reduced early nerve reflex barriers and white matter manifestations in mice, leading to decreased activation of microglia and release of proinflammatory cytokines. Additionally, ISL demonstrated the ability to mitigate impairment in oligodendrocyte development and myelination, ultimately improving behavior disorders in adult mice. Mechanistically, we observed that ISL downregulated HDAC3 expression, promoted histone acetylation, enhanced the expression of H3K27ac, and regulated oligodendrocyte pro-differentiation factors.

**Conclusion:**

These findings suggest that ISL can have beneficial effects on white matter injury in preterm infants by alleviating inflammation and promoting oligodendrocyte differentiation.

## Graphical Abstract

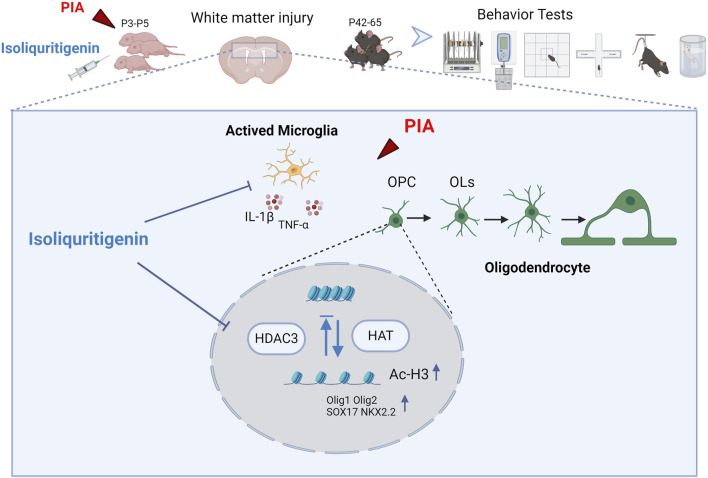

## 1 Introduction

According to the World Health Organization (WHO), approximately 15 million preterm babies are born every year, accounting for about one in ten live births. Preterm birth poses a significant international public health concern due to its close association with high rates of mortality and morbidity (Liu et al., 2016). Despite significant advances in preterm care that have greatly improved survival rates, there has been a concurrent rise in chronic neurological dysfunction among preterm infants (Wilson-Costello et al., 2005). Research indicates that a range of severe abnormalities in motor function, cognition, and emotion are present in 5–10 percent of preterm infants (Soria-Pastor et al., 2008; Volpe et al., 2011). It is important to recognize that brain injury in preterm infants not only detrimentally impacts their health and quality of life, but also imposes a significant economic burden on families and society (Kruse et al., 2009).

White matter injury is the primary form of brain injury observed in preterm infants (Volpe, 2009). Perinatal infection is widely recognized as one of the most significant risk factors for white matter injury (Edwards and Tan, 2006; Ledger, 2008). Infection-induced proinflammatory cytokines easily cross the blood-brain barrier and enter the brain parenchyma, subsequently activating microglia and initiating a detrimental cascade (Malaeb and Dammann, 2009; Kuypers et al., 2012). During the period of preterm birth, late oligodendrocyte progenitor cells (OPCs) are vulnerable to selective degeneration caused by adverse stimuli (Segovia et al., 2008). This damage disrupts the normal processes of oligodendrocyte maturation and myelination through aberrant regenerative and repair mechanisms (Back, 2017). Therefore, improving the inflammatory response of microglia and preserving the normal development of oligodendrocytes are critical therapeutic targets for white matter injury.

Isoliquiritigenin (ISL) is a compound extracted from licorice that possesses a chalcone structure. Numerous studies have demonstrated the diverse biological and pharmacological activities of ISL, including anti-inflammatory, antioxidant, and anti-tumor properties (Yadav et al., 2011; Peng et al., 2015). Notably, ISL exhibits notable neuroprotective effects (Shi et al., 2020). Research has revealed that ISL can inhibit the release of mitochondrial apoptosis-inducing factors, Bcl-2 and Bax, into the cytoplasm, suppress glutamate-induced reactive oxygen species (ROS) production, mitigate glutamate-induced damage to mitochondria, and prevent hippocampal neuronal death (Yang et al., 2012). Moreover, ISL has demonstrated the ability to enhance or maintain the antioxidant capacity of mice while inhibiting neuroinflammation (Zhu et al., 2019). Nevertheless, the role of ISL in white matter injury of the nervous system remains unexplored. This study aims to explore the therapeutic effects of ISL on white matter injury. First, network pharmacology will be used to predict the potential mechanisms by which ISL may treat white matter injury. Subsequently, an experimental model of white matter injury in premature infants will be established to verify whether ISL can reduce white matter injury and related neurobehavioral disorders, and to elucidate its specific mechanisms.

## 2 Material and methods

### 2.1 Animals and white matter injury mice model

The experiment was conducted using Kunming mice pups (n = 111). Approval for all animal procedures was obtained from the Ethics Committee on Animal Experiments of the Medical School of Shandong University. The dams and pups, with 9–11 pups per litter, were housed in animal facilities maintained at a constant temperature of 22°C ± 2°C, and subjected to a 12-h light/dark cycle. They were provided unlimited access to food and water. The day of birth was designated as postnatal day 1 (P1). In order to establish a white matter injury model, the mice were intraperitoneally injected with lipopolysaccharide (LPS) for three consecutive days, starting from P3.

### 2.2 Drug treatment

The experiment was conducted following previous studies (Pang et al., 2012; Fan et al., 2013; Xie et al., 2016). The experimental design and drug treatment schedule are presented in Figure 2A. The experimental animals were divided into three groups: the Control group (treated with vehicle solution), the LPS group (treated with LPS at a dosage of 1 mg/kg/24 h), and the LPS + ISL group (treated with LPS at a dosage of 1 mg/kg/24 h, and ISL at a dosage of 4 mg/kg/24 h). At the end of the experiment, the mice were euthanized in accordance with institutional guidelines and approved protocols. The euthanasia was carried out using carbon dioxide (CO2) inhalation, followed by cervical dislocation to ensure death. CO2 was introduced into the chamber at a rate that displaced 10%–30% of the chamber volume per minute. The animals were monitored for cessation of respiration and lack of a heartbeat before proceeding with the subsequent procedures. All efforts were made to minimize animal suffering and to ensure a humane endpoint. The brain was removed, and the corpus callosum was collected and quickly stored at −80°C until further use. Some of the mice were used in the behavioral experiments. The LPS (*Escherichia coli* strain O111:B4) used in the experiments was purchased from Sigma-Aldrich (St. Louis, MO, United States) and prepared in saline solution (0.9% NaCl). ISL (98.07% purity) was purchased from MCE (shanghai, China) and dissolved in sterile saline with 1% dimethyl sulfoxide (Solarbio, Beijing, China). The chosen dosage of the drug was based on the results of our study (Supplementary Figure 1A, B) and consistent with previous literature demonstrating the neuroprotective effects of the agent (Liu et al., 2019; Wang et al., 2020).

### 2.3 Behavioral tests

Behavioral tests of mice were conducted between P42 and P56. Every behavioral experiment was conducted in an individual, dark chamber, devoid of unwanted external light and sound. During the experiment, the operator was blinded to the mice in the different treatment groups. Mice were moved there at least 30 min in advance before the experiment to adapt to the environment. After each mouse experiment, the behavioral device was cleaned with 75% ethanol. After each experiment, the mice rested for 24 h, waiting for the next experiment. A tracking system (TopScan 3.0) was used to record, measure and analyze all behavioral experiments.

#### 2.3.1 Righting reflex

The mice were placed on the surface of the table with gentle pressure applied to both sides of their heads and hind limbs. Once released, the time taken by the mice to roll over and make contact with the table using all four limbs was recorded.

#### 2.3.2 Negative geotaxis

The mice were positioned with their heads facing downward on a 45° incline. The time required for the mice to rotate and lift their heads was observed and recorded. If the mice did not complete the task within 60 s, the test was terminated.

#### 2.3.3 Tail suspension test (TST)

The mice were suspended by their tails for 6 min and monitored using a video tracking system to assess escape-related behavior. The duration of immobility during the 6-min suspension period was recorded.

#### 2.3.4 Forced swim test (FST)

This test aimed to assess depression-like behavior in the mice. The forced swim tests were conducted in a cylindrical glass tank measuring 30 cm in height and 18 cm in width, filled with fresh water at a temperature of 25°C ± 2°C and a depth of 20 cm. The total immobility duration was recorded over a 6-min period, with immobility defined as the time when the mouse made minimal movements to keep its head above the water surface.

#### 2.3.5 Elevated plus maze (EPM)

This experiment aimed to evaluate anxiety-like behavior in mice. The apparatus used in this experiment consisted of two open arms and two closed arms. The dimensions of the open arms were 40 × 5 cm, while the dimensions of the closed arms were the same. All arms were surrounded by walls measuring 19 cm in height. The apparatus was positioned 50 cm above the ground. The mice were placed on the central platform of the maze, which measured 5 × 5 cm, facing towards an open arm. They were given 6 min to explore the maze, with locomotion data being collected using a video-tracking system.

#### 2.3.6 Open field test (OFT)

The mice were placed in a 40 cm × 40 cm field and allowed to move freely for a duration of 10 min. They were initially situated in the center of the arena, with the center region defined as the 20 cm × 20 cm area located at the center. The activities of the mice were observed and recorded using an overhead video camera. The total distance moved and the time spent in the center were analyzed using an animal behavioral tracking system. After each mouse’s test, the open field apparatus was thoroughly cleaned with a 75% ethyl alcohol solution.

#### 2.3.7 Grip strength

Grip strength was measured using a computerized grip strength meter. The apparatus comprised a metal bar connected to a force transducer. To measure the grip strength of the mouse’s whole body and fore paws, the experimenter held the mice at the base of the tail, enabling them to grip the metal bar with all four paws or just the fore paws. The experimenter pulled the mice backwards by their tails until they lost their grip. The peak force of each measurement was automatically recorded in grams (g) by the device. Three measurements of grip strength were taken in each mouse.

#### 2.3.8 Rotating rod test

In the first 2 days of training, mice were placed on a rotating rod that underwent linear acceleration, reaching a minimum speed of 10 RPM and a maximum speed of 40 RPM at a rate of 9.9 RPM/s. They were then maintained at a constant speed of 40 RPM for an additional 5 min. On the third day, the mice were placed on a rotating rod at a fixed speed of 40 RPM to assess motor function and coordination based on the latency to fall.

### 2.4 Luxol fast blue (LFB) staining

Cryosections selected from each group were rinsed with distilled water for 5 min and dehydrated using an ascending ethanol series. The sections were then stained with preheated (55°C) luxol fast blue stain (LFB; 0.1 g LFB in 100 mL of 90% ethanol with 0.5 mL of 10% glacial acetic acid) for an extended period. Finally, the sections were sealed using neutral balsam and examined under a microscope.

### 2.5 Transmission electron microscope

The brain tissue of mice 21 days after birth was cut into 1 mm^3^ and fixed with 3% glutaraldehyde at 4°C for 2 h. The fixed tissue was washed 3 times with phosphate-buffered saline (PBS). The tissue was then fixed in 1% OsO4 at 4°C for 2 h. The tissues were dehydrated with ethanol gradient and infiltrated with 100% epoxy resin: acetone (1:3, 1:1, 3:1) for 1 h, 4 h, and 12 h, respectively. Finally, the tissues were embedded in fresh epoxy resin and polymerized at 37°C for 12 h, 45°C for 12 h, and 60°C for 1 h. The ultra-thin slices were supported on a grid (150 nm) and stained at room temperature for 20 min with uranyl acetate and 20 min with lead citrate. Images were captured using a Talos F200C transmission electron microscope (FEI Company).

### 2.6 Immunofluorescence

The mice were anesthetized and then perfused with a 4% paraformaldehyde solution. Subsequently, the brain tissue was removed and fixed for frozen sectioning. The brain tissue sections were washed three times with PBS. Next, they were blocked with a mixture of 10% goat serum and 0.3% Triton X-100 for 3 h, followed by incubation with the primary antibody overnight at 4°C. The primary antibodies used were anti-Iba1 (1:200, Abcam), anti-PDGFR-α (1:20, CST), and anti-APC (1:50, Beyotime Biotechnology). Then, the cells or sections were incubated with fluorescently conjugated secondary antibodies (AbBkine-488 and AbBkine-594) for 1 h. The nuclei were stained with DAPI (Sigma-Aldrich Corp). The images were acquired using an IX71 Olympus fluorescence microscope.

### 2.7 Immunohistochemical staining

The tissues were fixed using a 4% paraformaldehyde solution, dehydrated, embedded in paraffin, and then sectioned. Following dewaxing and hydration, the sections were immersed in a 3% hydrogen peroxide solution for 10 min. Subsequently, they were blocked with either 10% normal goat serum or 10% normal donkey serum, and incubated overnight at 4°C with the primary antibody (Anti-MBP antibody was used at a dilution of 1:600, CST). After washing with PBS, the samples were incubated with a horseradish peroxidase (HRP)-conjugated secondary antibody for 1 h. Following that, they were stained with 3,3′-diaminobenzidine (DAB) and photographed.

### 2.8 Primary microglia isolation and cell culture

Neonatal mice were sacrificed on postnatal day 3 (P3), and the brain tissue was removed, and the cerebellum, olfactory bulb, hippocampus and meninges were stripped. The rest of the brain tissue was then digested using 0.25% trypsin. The mixed glial single-cell suspension was further resuspended in Dulbecco’s Modified Eagle Medium (DMEM) supplemented with 10% fetal bovine serum (FBS) and 1% Penicillin/Streptomycin/Amphotericin B (P/S/A) (100X). The cells were seeded into poly-lysine coated culture flasks at a density of approximately 5 × 10^6 cells/mL. On day 7, floating microglia were collected by gently tapping the culture flask. The isolated primary microglia were then seeded in either 6-well plates or 96-well plates for subsequent experiments. The cells were cultured in a humidified chamber at 37°C with 5% CO2.

### 2.9 Cell counting kit-8 (CCK-8) assay

The Cell Counting Kit-8 (CCK-8) assay (K1018, APExBIO) was performed to assess the impact of ISL on cell viability. Microglia were cultured in 96-well plates and exposed to varying concentrations of ISL for 24 h. Prior to testing, 100 μL of CCK-8 solution was added to each well of the culture plate and incubated in a CO2 incubator at 37°C for 2 h. The optical density at a wavelength of 450 nm was measured using a Microplate Reader (BIO-RAD iMark).

### 2.10 RNA isolation and real-time quantitative PCR

Mouse brain tissues were used to extract total RNA with TRIZOL reagent (Invitrogen). The purity and concentration of the total RNA were determined using a spectrophotometer (Thermo NanoDrop 2000). Subsequently, the cDNA was synthesized using the RevertAidTM First Strand cDNA Synthesis Kit (Thermo Fisher Scientific). Real-time PCR was performed with the SYBR Green Real-Time PCR Master Mix (TOYOBO CO., Ltd., Japan). β-actin expression served as the normalized control, and changes in gene expression were calculated using the 2^−ΔΔCT method. The primer sequences are provided in Table 1.

**TABLE 1:** Primer sequences used for q-PCR experiments.

q-PCR primer sequence (mouse)		
Actin	Forward	CGT​TGA​CAT​CCG​TAA​AGA​CCT​C
	Reverse	CCA​CCG​ATC​CAC​ACA​GAG​TAC
IL-1β	Forward	GCA​ACT​GTT​CCT​GAA​CTC​AAC​T
	Reverse	ATC​TTT​TGG​GGT​CCG​TCA​ACT
TNF-α	Forward	GAC​GTG​GAA​CTG​GCA​GAA​GAG
	Reverse	TTG​GTG​GTT​TGT​GAG​TGT​GAG
HDAC1	Forward	CAG​ACT​CAG​GGC​ACC​AAG​AGG​AAA
	Reverse	GGG​TGC​CCT​TGT​CCA​TAA​TAG​TAG
HDAC2	Forward	AGA​CAA​ATC​CAA​GGA​CAA​TAG​TGG​T
	Reverse	CAA​ATT​CAA​GGG​TTG​CTG​AGT​TGT
HDAC3	Forward	CAA​TCT​CAG​CAT​TCG​AGG​ACA​TG
	Reverse	GCA​ACA​TTT​CGG​ACA​GTG​TAG​CC
HDAC8	Forward	CAA​TCC​GAA​GGC​AGT​GGT​T
	Reverse	GCCAGCTGCCACTGTAGG
Olig1	Forward	TCT​TCC​ACC​GCA​TCC​CTT​CT
	Reverse	CCG​AGT​AGG​GTA​GGA​TAA​CTT​CG
Olig2	Forward	TCC​CCA​GAA​CCC​GAT​GAT​CTT
	Reverse	CGT​GGA​CGA​GGA​CAC​AGT​C
TCF4	Forward	CGA​AAA​GTT​CCT​CCG​GGT​TTG
	Reverse	CGT​AGC​CGG​GCT​GAT​TCA​T
NKX2.2	Forward	AAG​CAT​TTC​AAA​ACC​GAC​GGA
	Reverse	CCT​CAA​ATC​CAC​AGA​TGA​CCA​GA
ID2	Forward	ATG​AAA​GCC​TTC​AGT​CCG​GTG
	Reverse	AGC​AGA​CTC​ATC​GGG​TCG​T
ID4	Forward	CAG​TGC​GAT​ATG​AAC​GAC​TGC
	Reverse	GAC​TTT​CTT​GTT​GGG​CGG​GAT
SOX17	Forward	GAT​GCG​GGA​TAC​GCC​AGT​G
	Reverse	CCA​CCA​CCT​CGC​CTT​TCA​C
SOX9	Forward	GAG​CCG​GAT​CTG​AAG​AGG​GA
	Reverse	GCT​TGA​CGT​GTG​GCT​TGT​TC
SOX10	Forward	AGC​CCA​GGT​GAA​GAC​AGA​GA
	Reverse	CCC​CTC​TAA​GGT​CGG​GAT​AG

### 2.11 Western blot

The corpus callosum tissue was lysed in RIPA buffer at 4°C for 30 min. Following centrifugation, the supernatant was collected, and the total protein concentration was measured using the BCA method. Equal amounts of protein from each sample were then separated using 8%–15% SDS-PAGE gel electrophoresis and transferred onto PVDF membranes with pore sizes of either 0.22 or 0.45 μm (Millipore). Subsequently, the PVDF membrane was blocked using TBST buffer containing 5% milk and incubated overnight at 4°C with the primary antibody. The secondary antibody, either goat anti-mouse IgG or rabbit IgG conjugated to HRP, was used. Finally, the bands were visualized using the enhanced chemiluminescence (ECL) detection kit (Merck Millipore), and the analysis was performed using ImageJ software (Version 1.46r). Primary antibodies were used at the following dilutions: rabbit anti-HDAC1 (1:500, Proteintech), rabbit anti-HDAC2 (1:500, Proteintech), rabbit anti-HDAC3 (1:500, Proteintech), rabbit anti-HDAC8 (1:500, Proteintech), rabbit anti-H3 (1:500, CST), rabbit anti-H3K18ac (1:500, CST), rabbit anti-H3K9/14ac (1:500, CST), rabbit anti-H3K27ac (1:500, CST), rabbit anti-AcH3 (1:1000, CST), mouse anti-β-actin (1:2000, CST).

### 2.12 Chromatin immunoprecipitation assay

Enzymatic chromatin immunoprecipitation (ChIP) was conducted using the SimpleChIP^®^ Enzymatic Chromatin IP Kit (CST). Formaldehyde fixation was performed on the tissue, followed by lysis and partial digestion of chromatin using Micrococcal Nuclease provided in the kit. This digestion process resulted in fragments ranging from 200–500 base pairs. The chromatin was immunoprecipitated overnight at 4°C using the specific antibody anti-HDAC3 or the control antibody (anti-IgG). The DNA was subsequently purified using a DNA purification centrifuge column, and after protein-DNA uncross-linking according to the manufacturer’s instructions, it was analyzed using real-time PCR. The primer sequences used for the PCR analysis can be found in Table 2.

**TABLE 2:** Primer sequences used for ChIP-qPCR experiments.

ChIP-qPCR primer sequences (mouse).		
Olig1-#1	Forward	GGT​GTT​CCA​AGG​AGC​GAT​GT
	Reverse	CCC​TGC​CAG​TGG​GTG​AGT​T
Olig1-#2	Forward	CCA​TCG​GTG​TTC​GGA​CTT​AC
	Reverse	GCC​CAA​CTC​CGC​TTA​CTT​TA
Olig2-#1	Forward	TGA​GGA​GGC​AGG​AGA​TTA​G
	Reverse	GGGTCATTGTTCCCATTT
Olig2-#2	Forward	ATGGGCTCCACTTCCTCA
	Reverse	TCCCTCTATTGGGTTTCG
NKX2.2-#1	Forward	TATCTGCCTTGGACTCGC
	Reverse	AAATTGCTTGGTCGCTAA
NKX2.2-#2	Forward	TCTTTCCCTCCCACTTCT
	Reverse	CAT​AAA​CAT​CTG​GCT​TCA​C
SOX17-#1	Forward	CAGGGCAGTTGTGAGGGT
	Reverse	GAAAGCCAGGCTGAAGAT
SOX17-#2	Forward	TCCAGGATTGAAAGGTGT
	Reverse	GAG​GGA​AGA​GTT​AGG​AAG​C

### 2.13 Access to potential targets for ISL and white matter injury

The chemical structure and specification of ISL smile was downloaded from PubChem (https://pubchem.ncbi.nlm.nih.gov/). Importing the information into the PharmMapper server (https://www.lilab-ecust.cn/pharmmapper/) and Swiss Target Prediction (http://www.swisstargetprediction.ch/) allowed identification of ISL related targets. Potential targets for white matter injury were identified using “white matter injury” as a search term in the Genetic Society database (https://www.genecards.org/). Subsequently, the intersection of ISL and WMI targets was identified by analyzing the two sets of targets using Venny software (https://bioinfogp.cnb.csic.es/tools/venny/). The STRING database (https://cn.string-db.org/cgi/input.pl) can provide network visualization. Based on the STRING database, the interaction genes between ISL and WMI targets were analyzed (confidence data >0.4).

### 2.14 Bioinformatics analysis

Kyoto Encyclopedia of Genes and Genomes (KEGG) and Gene Ontology (GO) pathway enrichment analyses were performed on an online bioinformatics platform (https://www.genedenovo.com/) to elucidate key signaling pathways and identify biological functions associated with potential therapeutic targets for ISL.

### 2.15 Statistical analysis

The data are presented as the mean ± standard error of the mean (SEM). Statistical analysis was performed using one-way analysis of variance (ANOVA) followed by Tukey’s test. Statistically significant differences were defined as *p* values <0.05.

## 3 Results

### 3.1 Network pharmacology predicts the targets of ISL effect on white matter injury

**FIGURE 1:**
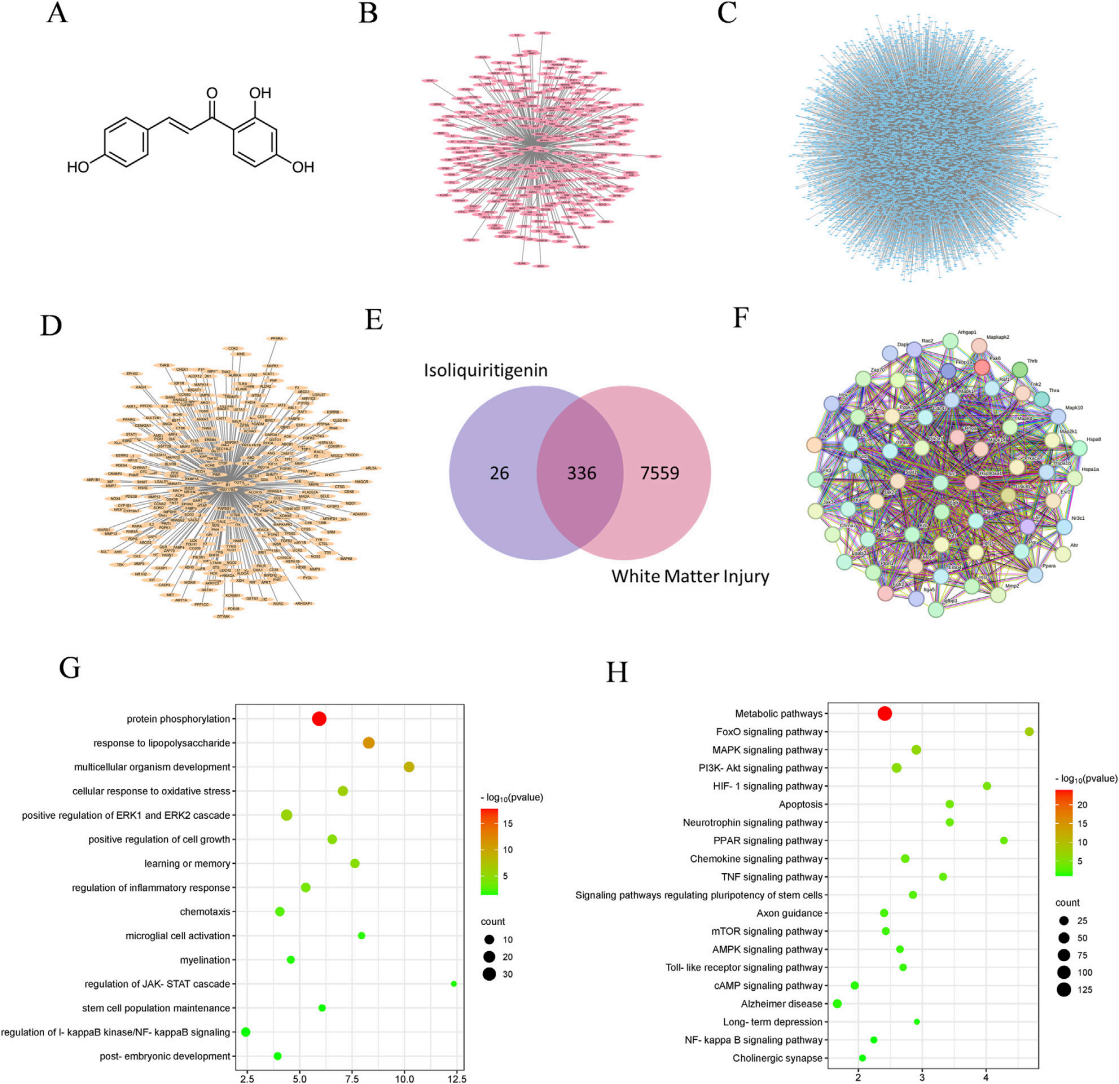
The analysis of network pharmacology. **(A)** Molecular formula of ISL. **(B)** The targets of ISL. **(C)** WMI targets. **(D)** Potential therapeutic targets of ISL for WMI. **(E)** Venn diagram. **(F)** Protein-protein interaction network. **(G)** GO enrichment analysis. **(H)** KEGG enrichment analysis.

A total of 362 potential targets for ISL were predicted using the Swiss Target Prediction and PharmMapper databases (Figures 1A, B; Supplementary Table 1). Of these, 336 therapeutic targets were identified by intersecting with 7,895 WMI-related targets (Figures 1C–E; Supplementary Tables 2, 3). Results from STRING analysis show that there are 321 targets in the PPI network, including 113 nodes and 3,445 edges, with an average node degree of 21.5 (Figure 1F). To analyze the role of these targets, GO enrichment analyses were performed to assess the biological functions of ISL targets (Figure 1G). The results indicated significant associations with inflammation, microglia cell activation, and myelination, which are important mechanisms of ISL treatment for WMI. Additionally, KEGG enrichment analysis suggested that ISL may affect WMI through multiple signaling pathways, including the MAPK signaling pathway, PI3K-Akt signaling pathway, Toll-like receptor signaling pathway, and NF-κB signaling pathway (Figure 1H).

### 3.2 ISL attenuates weight loss and motor coordination

**FIGURE 2:**
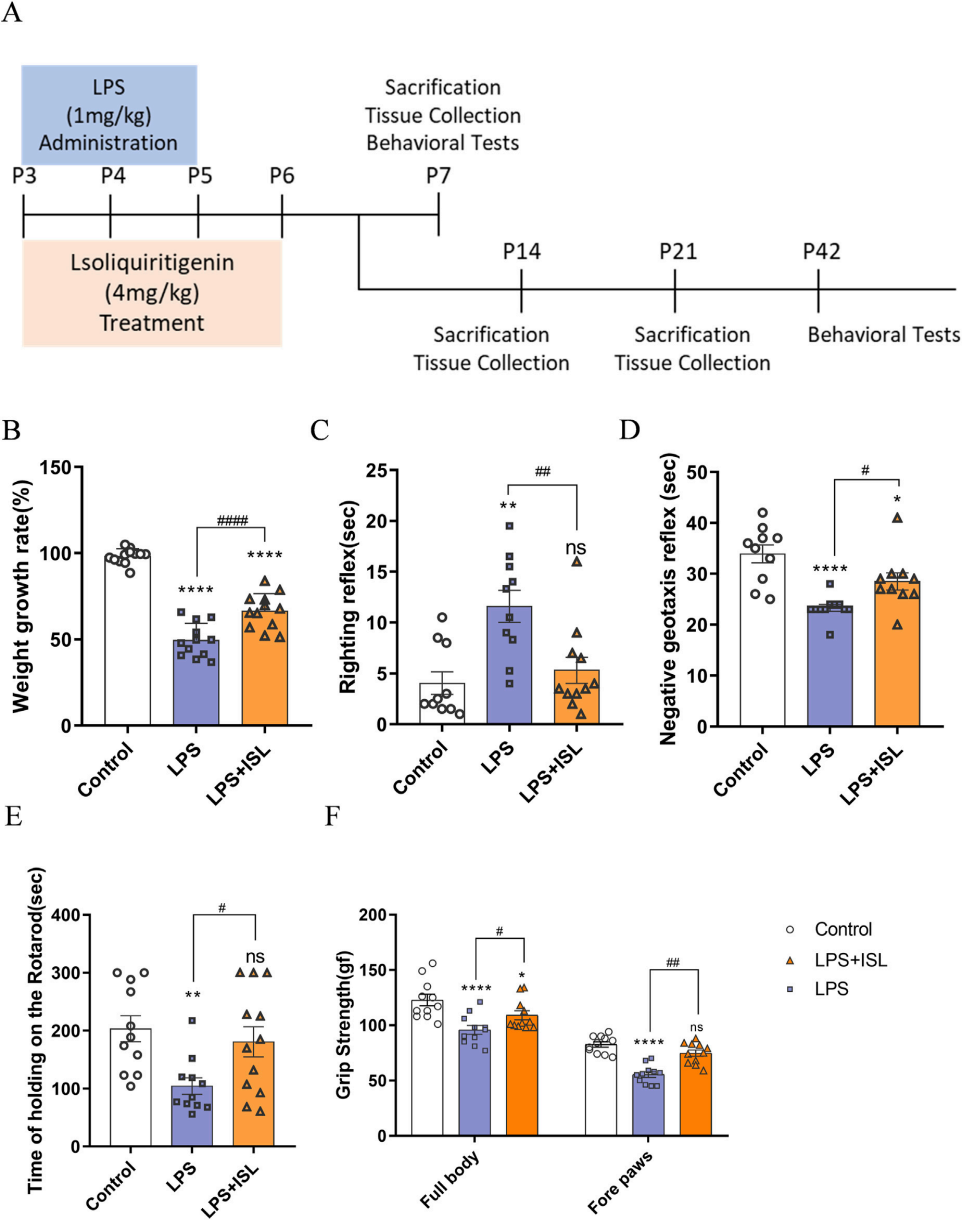
Isoliquiritigenin attenuated body weight loss and motor capacity in mice. **(A)** Schematic representation of the early induction of systemic inflammation. **(B)** The increase in body weight of the pups at day 7 compared with day 3 after birth (%) (n = 12). **(C,D)** Performance of righting reflex and negative geotaxis (n = 10–11). **(E)** Rotating rod test for the motor coordination evaluation of each group (n = 11). **(F)** Grip Strength test for the whole body and forelimb of each group (n = 11). The results are shown as the mean ± SEM, and analyzed by one-way ANOVA followed by *post hoc* Turkey test. *p < 0.05, **p < 0.01, ***p < 0.001, ****p < 0.0001 compared with the control group. #*p* < 0.05, ##*p* < 0.01, ###*p* < 0.001, ####*p* < 0.0001 compared with the LPS group. “ns” indicates not significant.

To investigate the potential of ISL in improving behavioral abnormalities in mice with white matter injury, we initially assessed body weight and early neural reflexes (Figure 2A). The righting reflex and negative geotaxis are commonly used neurobehavioral tests in rodent research, which we utilized to assess early motor coordination function in mice development. Compared to the control group, mice subjected to LPS-induction exhibited significant reductions in body weight and early neurobehavioral deficits. ISL treatment significantly improved the body weight loss (Figure 2B). The righting reflex and negative geotaxis experiments showed that ISL treatment shortened the latency (Figures 2C, D). Furthermore, we conducted the rotary rod and grip tests in mice at P42 to evaluate the impact of ISL treatment on mice motor function. The findings demonstrated that ISL treatment significantly enhanced motor coordination impaired by LPS induction (Figure 2E) and grip reduction in mice (Figure 2F).

### 3.3 ISL ameliorates depressive- and anxiety-like behaviors in adult mice

**FIGURE 3:**
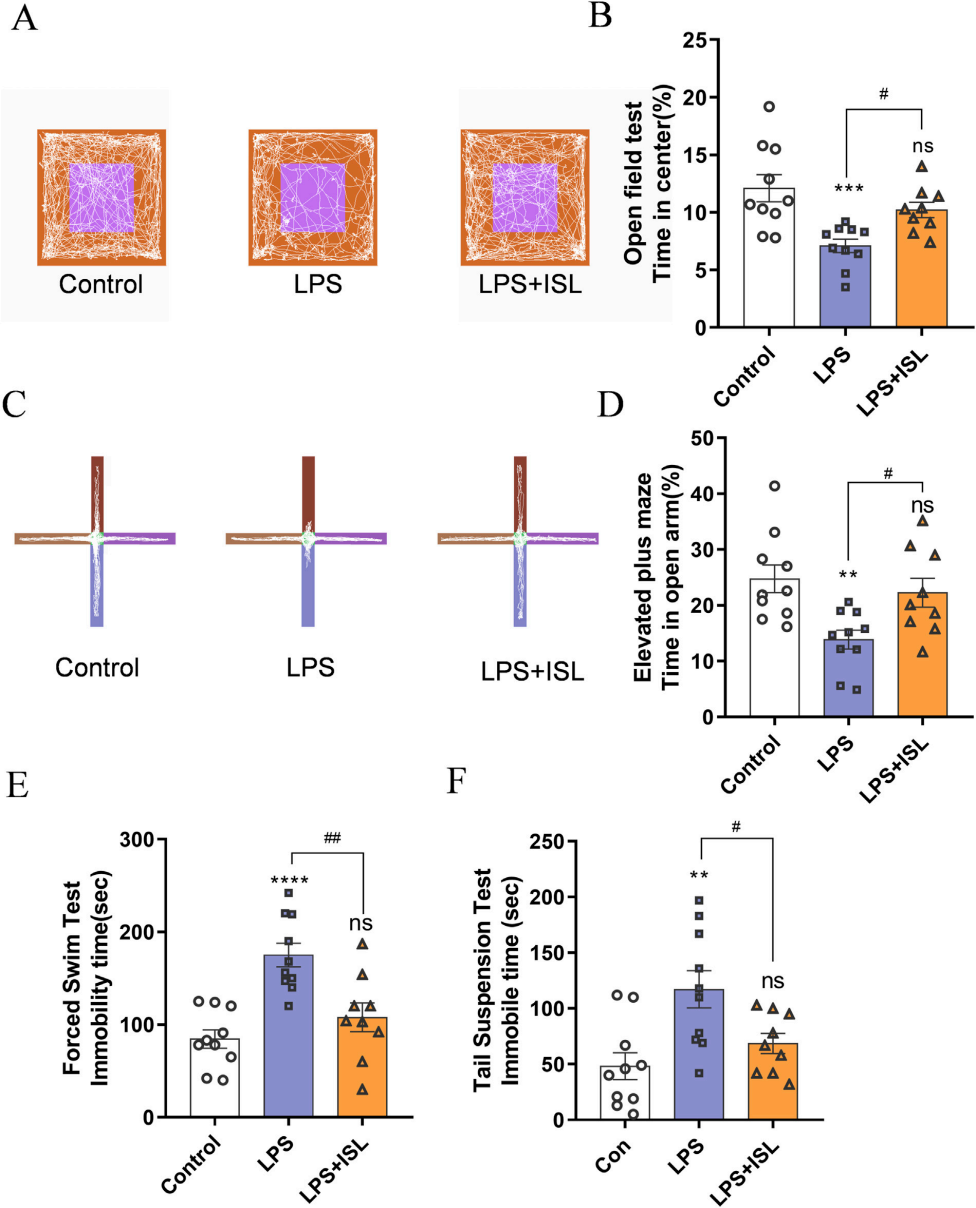
Isoliquiritigenin ameliorates anxiety-like behavior and depressive-like behavior. **(A)** Representative trajectory of mice in the open field test. Blue line square represents the center zone. **(B)** The percentage of time spent in the central region of the open field test (n = 9–10). **(C)** Representative trajectory of mice in the elevated plus maze test. **(D)** The duration (%) of stay in the open arm region in elevated plus maze test (n = 9–10). **(E)** The immobility time of different treatment groups in forced swimming test (n = 8). **(F)** The immobility time of different treatment groups in tail suspension test (n = 8). The results are shown as the mean ± SEM, and analyzed by one-way ANOVA followed by *post hoc* Turkey test. *p < 0.05, **p < 0.01, ***p < 0.001, ****p < 0.0001 compared with the control group. #*p* < 0.05, ##*p* < 0.01, ###*p* < 0.001, ####*p* < 0.0001 compared with the LPS group. “ns” indicates not significant.

To further explore the enduring impacts of ISL treatment, we analyzed the effect on emotional behavior in mice. We conducted the open field test (OFT) to assess anxiety-like behavior. Mice in the LPS group spent less time in the center, indicating increased anxiety. ISL treatment increased center time, suggesting reduced anxiety (Figures 3A, B). Additionally, the elevated plus maze (EPM) confirmed higher anxiety levels in the LPS group, with reduced time in open arms. ISL-treated mice spent more time in open arms, indicating decreased anxiety (Figures 3C, D). We evaluated depressive-like behavior using the forced swim test (FST) and tail suspension test (TST). LPS group mice showed longer immobility periods, indicative of increased depressive-like behavior. ISL treatment significantly reduced immobility time in both tests, suggesting an antidepressant effect (Figures 3E, F). These findings collectively demonstrate the potential of ISL treatment to mitigate emotional deficits in mice.

### 3.4 ISL improves LPS-induced white matter injury in mice

**FIGURE 4:**
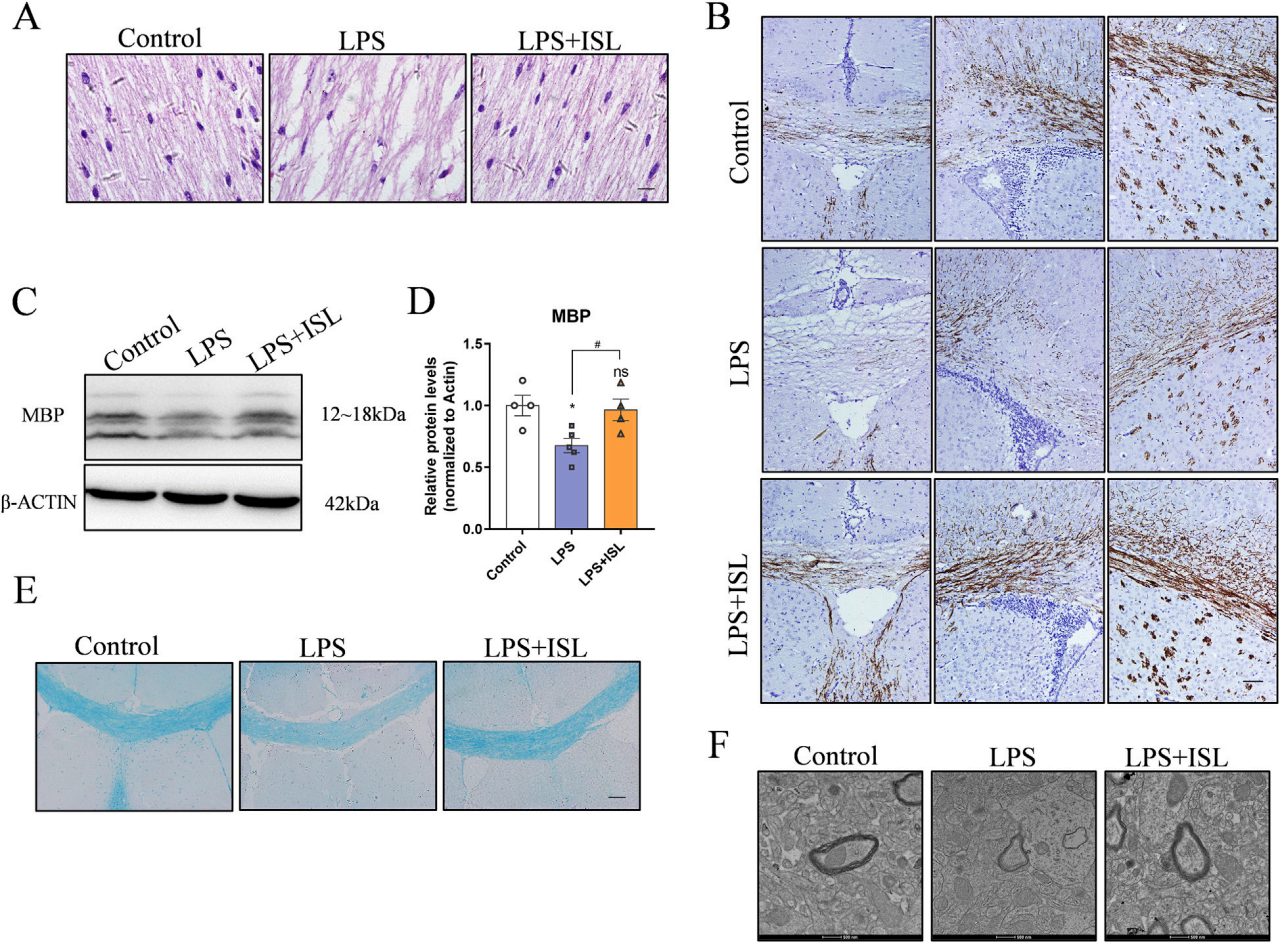
ISL alleviated white matte injury in the neonatal mouse brain. **(A)** Representative images of hematoxylin and eosin (HE) staining of corpus callosum and cingulate. scale bar = 10 μm. **(B)** Representative images of immunohistochemistry and relative quantification analysis of the expression of MBP in the corpus callosum, cingulate and corpus striatum. **(C,D)** Representative Western blot images and quantification of MBP expression of the callosum. **(E)** Representative Luxol Fast Blue staining of the corpus callosum. Scale bar = 100 μm. **(F)** Representative images of myelin ultrastructure. Scale bar = 100 nm. n = 3–4 for each group. The results are shown as the mean ± SEM, and analyzed by one-way ANOVA followed by *post hoc* Turkey test. *p < 0.05, **p < 0.01, ***p < 0.001, ****p < 0.0001 compared with the control group. #*p* < 0.05, ##*p* < 0.01, ###*p* < 0.001, ####*p* < 0.0001 compared with the LPS group. “ns” indicates not significant.

The abnormal structure of white matter may lead to impaired or disrupted neural signal transmission, thereby affecting emotional regulation and motor coordination. Therefore, we conducted a pathological examination of the brain tissue. HE staining revealed that the LPS group exhibited subcortical and periventricular white matter lesions compared to the control group. The ISL group showed a noticeable improvement in the periventricular white matter looseness and nerve fiber disorder, with some white matter structures returning to normal (Figure 4A). Neonatal white matter injury can result in impaired myelination. Myelin basic protein (MBP) is a critical component of the myelin sheath, playing an essential role in the process of myelination. MBP is crucial for maintaining the structural integrity and function of myelin, and its expression levels are often used as an indicator of myelination status in the central nervous system. Immunohistochemical staining of MBP revealed a decrease in MBP expression within the corpus callosum of the LPS group compared to the control group. The ISL group exhibited increased expression of MBP compared to the LPS group (Figure 4B). Furthermore, Western blot analysis indicated that ISL treatment was able to reverse the decreased expression of MBP induced by LPS (Figures 4C, D). The density of LFB-staining myelin in the callosum was significantly increased upon ISL treatment, as observed in Figure 4E. The results of myelin transmission electron microscopy scanning showed that there were fewer myelinated axons in the corpus callosum of the LPS group, with irregular myelin shape and loose structure, while the myelinated axons in ISL group were significantly improved (Figure 4F). Collectively, our data strongly suggest that ISL has the potential to ameliorate neonatal white matter injury and prevent myelination defects.

### 3.5 ISL inhibits microglial activation and pro-inflammatory gene expression

**FIGURE 5:**
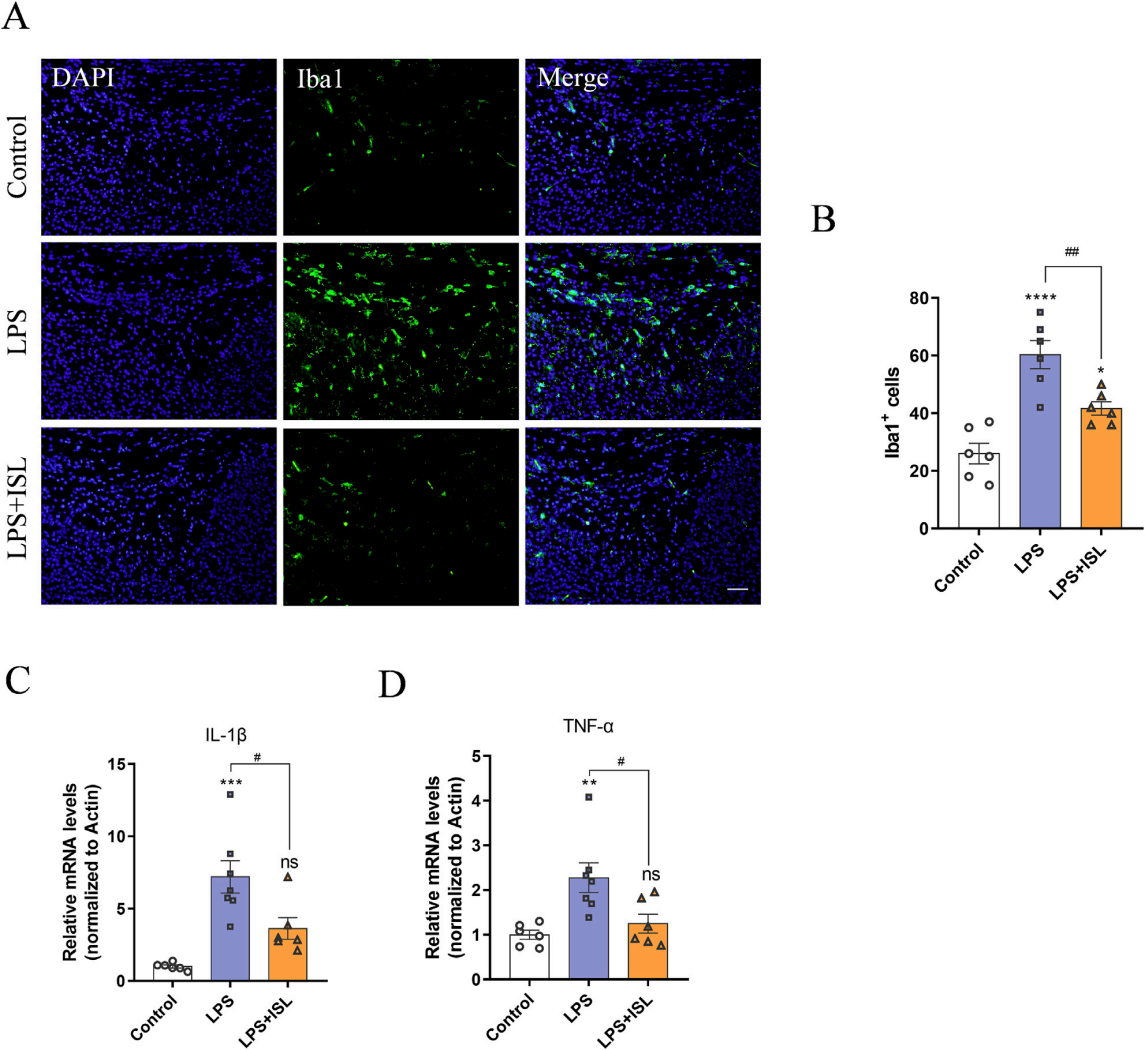
Isoliquiritigenin inhibits LPS-induce microglial activation and pro-inflammatory gene expression. **(A,B)** Representative images and quantification of immunofluorescence analysis of Iba1 (green) expression level from different groups (n = 6). **(C,D)** Quantification of the mRNA level of IL-1β and TNF-α in the brain (n = 6–7). Scale bar = 50 μm. The results are shown as the mean ± SEM, and analyzed by one-way ANOVA followed by *post hoc* Turkey test. *p < 0.05, **p < 0.01, ***p < 0.001, ****p < 0.0001 compared with the control group. #*p* < 0.05, ##*p* < 0.01, ###*p* < 0.001, ####*p* < 0.0001 compared with the LPS group. “ns” indicates not significant.

GO analysis showed that ISL treatment was associated with microglial activation and inflammatory response. Microglia-mediated neuroinflammation plays a crucial role in white matter injury. We aimed to assess the potential of ISL in regulating the inflammatory response in a model of LPS-induced white matter injury. We examined the quantity and morphology of microglia in the corpus callosum region, as well as the mRNA expression levels of classical inflammatory factors secreted by microglia. In Figures 5A, B, the number of Iba1+ microglia (green) in the LPS group was higher compared to the control group, indicating that systemic LPS exposure activated microglia. Conversely, the number of Iba1+ microglia (green) in LPS + ISL treated mice was significantly reduced (Figure 5B). The mRNA expression analysis also confirmed that ISL can reduce the upregulation of LPS-induced inflammatory cytokines IL-1β (Figure 5C) and TNF-α (Figure 5D). We additionally assessed the impact of ISL on LPS-induced microglia activation *in vitro*. When microglia were treated with different concentrations of ISL, it was found that 40 μM of ISL did not significantly affect microglia viability (Supplementary Figure 2A). Increasing the dose of ISL significantly decreased microglia viability. Based on the results of CCK8, a dose of 40 μM was chosen for subsequent experiments. Immunofluorescence staining of microglia revealed that ISL treatment significantly improved their activation state (Supplementary Figure 2B). Furthermore, qPCR results demonstrated that the expressions of IL-1β and TNF-α in microglia were significantly downregulated in the ISL treatment group compared to the LPS group (Supplementary Figures 2C, D). These findings suggest that ISL may inhibit microglia activation and suppresses proinflammatory gene expression.

### 3.6 ISL rescues oligodendrocyte development disorder

**FIGURE 6:**
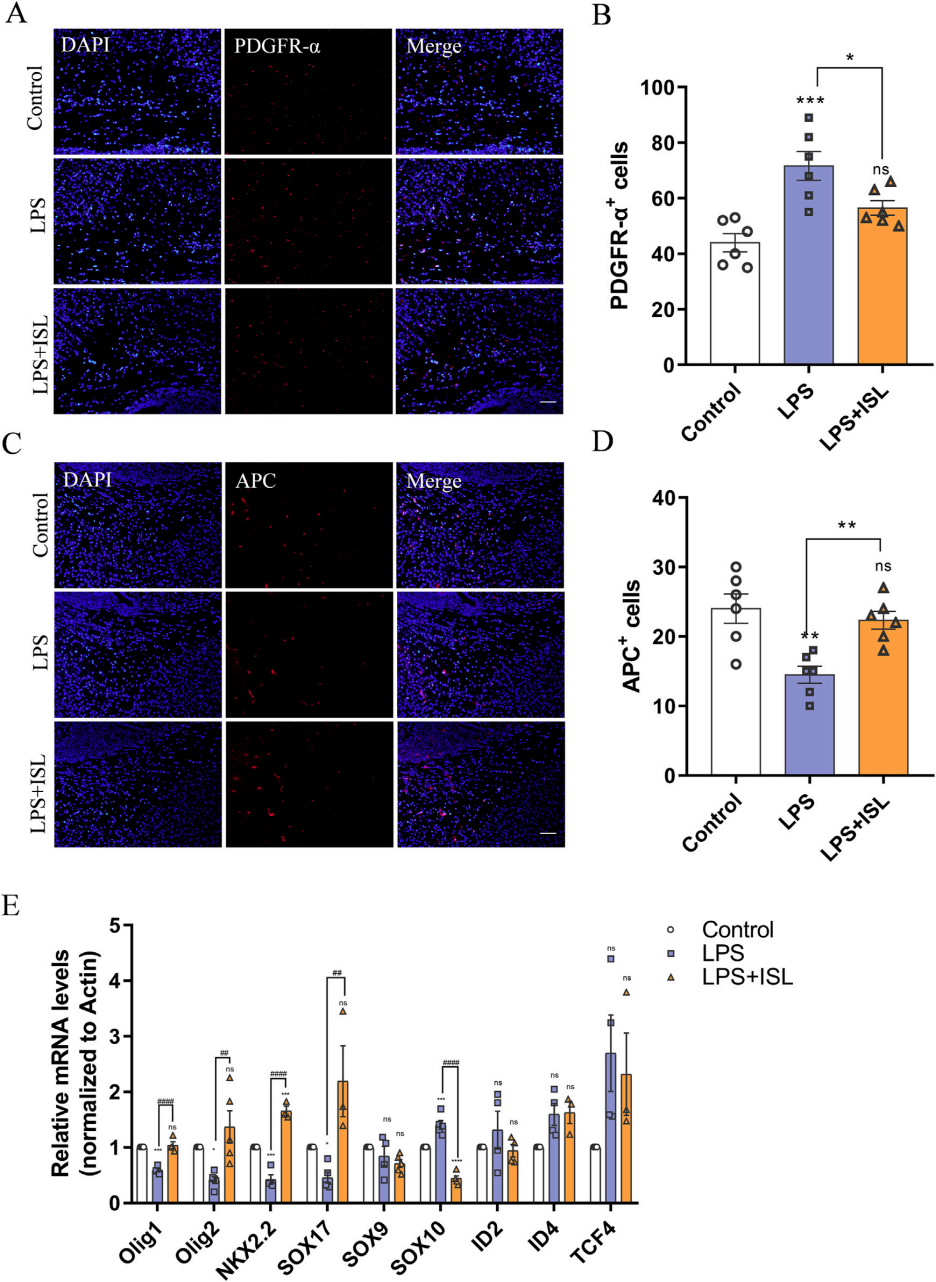
Isoliquiritigenin rescues oligodendrocyte dysplasia. **(A)** Representative PDGFR-α stained (red) brain sections of the callosum. Scale bar = 50 μm. **(B)** Quantitative analysis of the percentage of PDGFR-α^+^cells in the callosum (n = 6). **(C)** Representative APC stained (red) brain sections of the callosum. Scale bar = 50 μm. **(D)** Quantitative analysis of the percentage of APC^+^cells in the callosum (n = 6). **(E)** Statistical analysis of the expression of Olig1, Olig2, NKX2.2, SOX17, SOX9, SOX10, ID2, ID4 and TCF4 at mRNA level of each group (n = 3–6). The results are shown as the mean ± SEM, and analyzed by one-way ANOVA followed by *post hoc* Turkey test. *p < 0.05, **p < 0.01, ***p < 0.001, ****p < 0.0001 compared with the control group. #*p* < 0.05, ##*p* < 0.01, ###*p* < 0.001, ####*p* < 0.0001 compared with the LPS group. “ns” indicates not significant.

According to the biological process results of GO enrichment analysis, ISL treatment is related to myelination process. White matter injury primarily arises from a developmental disorder in oligodendrocyte precursor cells, resulting in defects in myelination and subsequent behavioral changes. In this study, we sought to investigate how ISL affects oligodendrocyte precursor cells. Immunofluorescence staining targeting PDGFR-α revealed an increase in the number of PDGFR-α^+^ cells in the LPS group. However, ISL treatment was found to restore the number of PDGFR-α+ cells reduced by LPS exposure (Figures 6A, B). Additionally, compared to the control group, the LPS group exhibited a decrease in the number of mature oligodendrocyte APC^+^ cells. However, in the LPS + ISL group, more APC^+^ cells were observed compared to the LPS group (Figures 6C, D). The development of oligodendrocytes involves numerous transcription factors and epigenetic regulation. To further investigate this, we conducted qPCR to examine changes in transcription factors known to play a role in oligodendrocyte maturation and differentiation. Our findings indicate an increase in the expression of SOX10 and a decrease in the expression of Olig1, Olig2, and NKX2.2 in the LPS group. However, treatment with ISL reversed these changes (Figure 6E). These results suggest that ISL may exert a beneficial effect on oligodendrocyte maturation by reducing the population of immature oligodendrocytes and increasing the population of mature oligodendrocytes. It is plausible that this effect is mediated through the influence of ISL on transcription factors involved in oligodendrocyte development.

### 3.7 ISL protects white matter by inhibiting HDAC3

**FIGURE 7:**
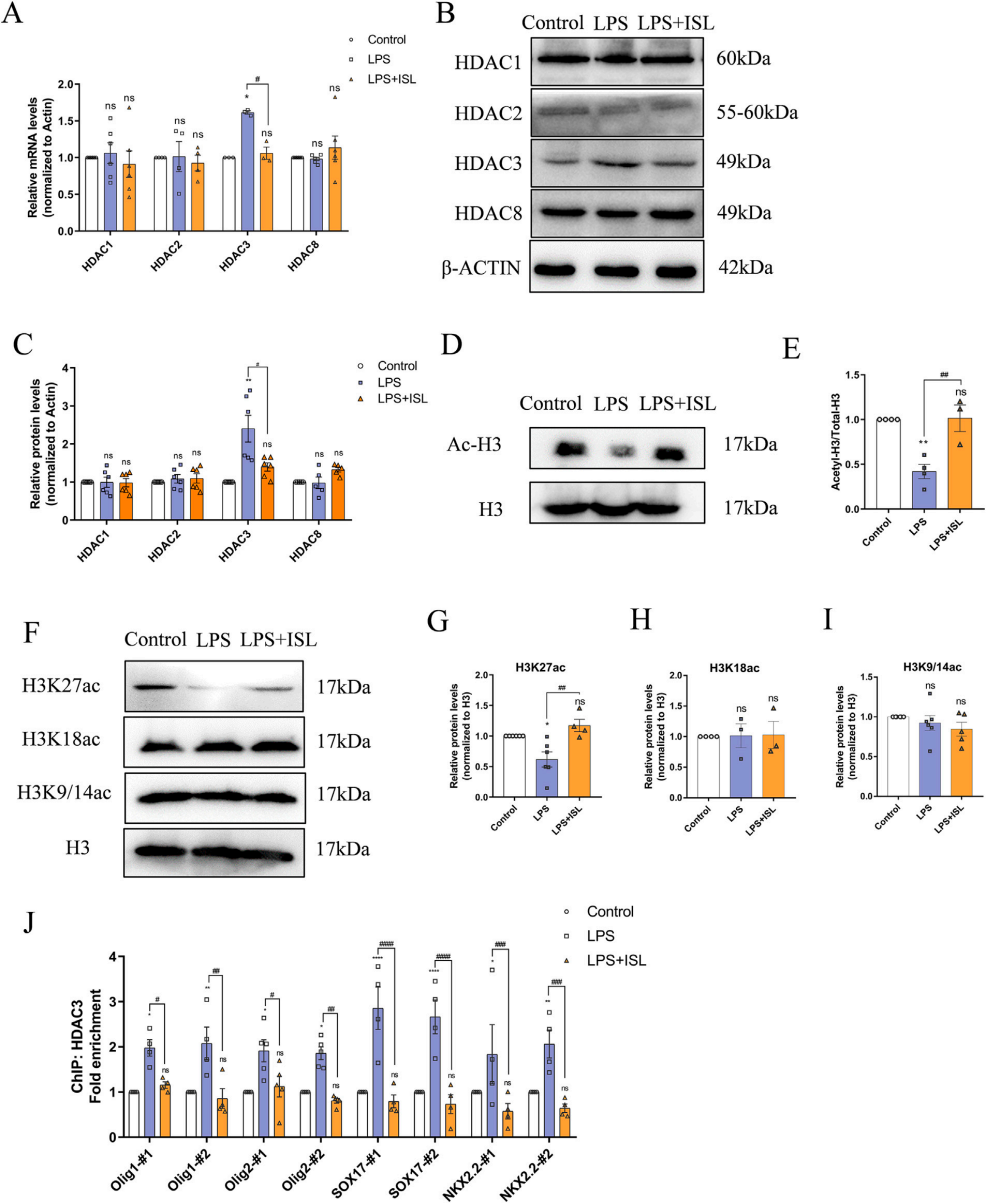
Isoliquiritigenin regulates histone acetylation and the expression of oligodendrocyte development related genes through HDAC3. **(A)** Statistical analysis of the expression of HDAC1, HDAC2, HDAC3 and HDAC8 at mRNA level of each group. **(B,C)** Representative Western blot images and relative quantification of HDAC1, HDAC2, HDAC3 and HDAC8. **(D,E)** Representative Western blot images and relative quantification of Ac-H3. **(G–I)** Representative Western blot images and quantification of histone acetylation expression in the brain. **(J)** ChIP-qPCR results showed the localization changes of HDAC3 at the promoters of transcription factors related to oligodendrocyte development. n = 3–6 for each group. The results are shown as the mean ± SEM, and analyzed by one-way ANOVA followed by *post hoc* Turkey test. *p < 0.05, **p < 0.01, ***p < 0.001, ****p < 0.0001 compared with the control group. #*p* < 0.05, ##*p* < 0.01, ###*p* < 0.001, ####*p* < 0.0001 compared with the LPS group. “ns” indicates not significant.

Class I HDACs play a crucial role in neuroinflammation and the development of oligodendrocytes. Several studies have reported that ISL is capable of inhibiting class I HDACs. Therefore, we investigated the potential of ISL to ameliorate white matter injury through the regulation of class I HDACs. Expression levels of HDAC1, HDAC2, HDAC3, and HDAC8 were measured, demonstrating that ISL could suppress the upregulation of HDAC3 mRNA and protein expression following LPS induction (Figures 7A–C). HDACs are known to regulate histone acetylation; therefore, we assessed changes in histone acetylation. Western blot analysis revealed a decrease in total acetylation of histone H3 following LPS treatment, whereas ISL treatment significantly increased its acetylation (Figures 7D, E). Additionally, we assessed histone acetylation at key sites. Our findings demonstrated a significant decrease in H3K27ac levels in the LPS group, while no significant changes were observed in H3K9/14ac and H3K18ac. Treatment with ISL significantly increased H3K27ac levels (Figures 7F–I). Furthermore, chromatin immunoprecipitation (ChIP) results demonstrated that ISL effectively inhibited the LPS-induced upregulation of HDAC3 binding to the promoter regions of essential transcription factors involved in oligodendrocyte pro-differentiation, such as Olig1, Olig2, SOX17, and NKX2.2 (Figure 7J). Our findings indicate that ISL is capable of modulating histone acetylation levels and the expression of transcription factors involved in oligodendrocyte development through its interaction with HDAC3.

## 4 Discussion

White matter consists of myelin nerve fibers surrounded by oligodendrocytes and plays a crucial role in various brain functions such as signal transmission, interregional communication, learning and memory, emotional regulation, and motor coordination (Filley and Fields, 2016; Chen et al., 2021). Among survivors of preterm birth, white matter injury stands as the primary form of brain damage (Volpe, 2009). The period of highest risk for white matter injury falls between approximately 23–32 weeks after conception (Back, 2017). During this critical window period, oligodendrocyte progenitors are particularly vulnerable to infection and inflammation, which impede their maturation and myelination processes, which in turn affect the structure and function of white matter (Segovia et al., 2008). Early intervention significantly mitigates oligodendrocyte development impairment, thereby preserving myelination and reducing associated sequelae (McClendon et al., 2014). LPS is a Gram-negative bacterial endotoxin that triggers an inflammatory response (Wang et al., 2006). Studies have found that in rodents, early exposure to LPS increases the risk of behavioral changes in adulthood (Dinel et al., 2014). Previous studies have shown that LPS stimulation can also induce oligodendrocyte apoptosis and abnormal differentiation in premature mice (Favrais et al., 2011). Therefore, we intraperitoneally injected mice aged 3–5 days with LPS to induce systemic and brain tissue inflammation, simulating perinatal infection-induced white matter injury in preterm infants. Building upon this, we explore the therapeutic potential of ISL in mitigating white matter damage and associated behavioral deficits in mice. In this study, a drug-target network of 336 targets of ISL for WMI was constructed using network pharmacological analysis. These targets were analyzed and corresponding to GO and KEGG enrichment items, and then verified by experiments. The results suggest that ISL treatment can reduce weight loss and early neuroreflex disorders in mice with LPS-induced white matter injury. It can inhibit the inflammatory response of microglia, improve the brain microenvironment of newborn mice, prevent oligodendrocyte dysplasia and myelin formation defects, and improve the motor coordination ability and anxiety- and depression-like behaviors of adult mice with white matter injury. From the perspective of potential mechanisms, we demonstrated that isoliquiritin can downregulate the expression of HDAC3 and regulate oligodendrocyte pro-differentiation factors through epigenetic modification, thus influencing the development of oligodendrocytes.

ISL has a wide range of pharmacological effects, including anti-inflammatory, antioxidant and anti-tumor properties (Peng et al., 2015; Liu et al., 2020; Liu et al., 2022; Bai et al., 2023). In the nervous system, studies have shown that it can inhibit the release of mitochondrial apoptotic factors Bcl-2 and Bax into the cytoplasm, suppress the production of glutamate-induced reactive oxygen species (ROS), alleviate glutamate-induced mitochondrial damage, and prevent hippocampal neuron death (Yang et al., 2012). However, the impact of ISL on oligodendrocytes has remained unknown. The maturation process of oligodendrocytes is complex, involving differentiation from neural stem cells into oligodendrocyte progenitor cells (OPCs), which proliferate and differentiate into pre-oligodendrocytes before becoming mature oligodendrocytes (Zou et al., 2023). During this process, oligodendrocytes gradually contact axons and generate myelin sheaths (Takebayashi and Ikenaka, 2015). In the GO_BP analysis, the target is related to the myelination process. Consistent with the GO results, our research suggests that the protective effect of ISL on oligodendrocyte development and white matter structure restoration may be an important mechanism for treating white matter injuries. Through specific analysis of the corpus callosum region in mouse brains, we demonstrate that ISL treatment can promote oligodendrocyte maturation and increase myelination. Transcription factors Nkx2.2, Olig1/2, Sox10, and MYRF promote the expression of genes related to oligodendrocyte differentiation and myelination (van Tilborg et al., 2016). Our research findings indicate that perinatal infection inhibits oligodendrocyte differentiation and leads to abnormal expression of oligodendrocyte differentiation-related transcription factors. Treatment with ISL can rescue abnormal differentiation of OPCs and increase the expression of pro-differentiation factors (Nkx2.2, Olig1/2, and Sox10), while reducing the expression of differentiation inhibitors (ID2 and ID4).

Microglia are inherent immune cells of the central nervous system and play a crucial role in initiating and maintaining the neuroinflammatory response (Wolf et al., 2017). When exposed to environmental cues, microglia proliferate and transition into either a pro-inflammatory or anti-inflammatory state, thereby adjusting the secretion of factors released into the brain environment. Under physiological conditions, microglia can support oligodendrocyte lineage cell development and myelination (Rahimian et al., 2022). Histological analysis of the brain tissue of preterm infants with diffuse white matter injury revealed the presence of active and pro-inflammatory microglia (Van Steenwinckel et al., 2024). High levels of pro-inflammatory cytokines, such as TNF-α and IL-1β, have also been observed in the brains of preterm infants with white matter injury, negatively affecting the proliferation, differentiation, and survival of immature oligodendrocytes *in vivo* and *in vitro* (Xie et al., 2016; Bonora et al., 2014). Previous studies have attributed the neuroprotective effect of ISL to its immunomodulatory effects. For example, studies have found that ISL regulates microglia activation by activating the Nrf2-HO-1 antioxidant axis (Foresti et al., 2013). Similarly, in the GO and KEGG analyses, changes in signaling pathways related to microglia activation and inflammation regulation were revealed. Our study also found that ISL effectively reduced microglia activation and the release of inflammatory factors in the corpus callosum region of mice with white matter injury. These results suggest that ISL may reduce disorders of oligodendrocyte differentiation and myelination by improving the microenvironment of the brain by improving the microglial inflammatory response.

Finally, we have demonstrated for the first time that ISL exerts neuroprotective effects in neonatal mice with white matter injury through epigenetic regulation. Previous studies have shown that ISL inhibits the activity of HDAC3 (Orlikova et al., 2012). Chromatin remodeling is a critical epigenetic process that plays a crucial role in oligodendrocyte development. Chromatin remodeling enzymes regulate gene expression by altering the accessibility of DNA to transcription factors (Yuan et al., 2020). HDACs are histone deacetylases. Among them, HDAC3 is upregulated during oligodendrocyte lineage development, and its expression is associated with oligodendrocyte differentiation (Zhang et al., 2016). Our results support these findings, suggesting that LPS treatment upregulates HDAC3 expression, while ISL significantly inhibits LPS-induced HDAC3 expression. Further molecular experiments demonstrate that ISL reduces the binding of HDAC3 to the promoter regions of pro-differentiation transcription factors in oligodendrocytes. Therefore, we suggest that ISL may regulate oligodendrocyte development by regulating HDAC3 activity. In epigenetic studies, histone modification is an important component of epigenetic regulation (Berson et al., 2018). Thus, we analyzed changes in relevant histone modifications. The experiments show that ISL increases the overall acetylation levels of histone H3, with the H3K27ac3 modification showing the most significant changes, but its specific regulatory targets need to be further studied.

While our findings emphasize the neuroprotective role of ISL in white matter injury, it is crucial to consider other drugs that modulate immune responses and protect white matter, particularly dimethyl fumarate (DMF). DMF has been extensively studied in multiple sclerosis (MS) and other neurodegenerative conditions, demonstrating its ability to activate the Nrf2 pathway, which enhances antioxidant responses and inhibits the production of pro-inflammatory cytokines (Linker and Haghikia, 2016; Yan et al., 2021). Furthermore, recent studies indicate that DMF modifies the expression of histone deacetylases (HDACs) in astrocytes, crucial for regulating inflammatory responses (Kalinin et al., 2013). DMF also reduces the density of microglia associated with white matter dysfunction, supporting its potential clinical value (Fowler et al., 2018). In contrast, our study reveals that ISL downregulates HDAC3 expression, enhances histone acetylation, and promotes oligodendrocyte differentiation, highlighting its unique mechanisms. Notably, ISL’s ability to modulate microglial activity and reduce inflammation presents a distinct advantage in treating white matter injury, especially in preterm infants, where inflammation plays a critical role in pathogenesis. This suggests that ISL may not only share epigenetic regulatory pathways with DMF but may also offer additional therapeutic benefits specifically tailored to the needs of vulnerable populations. Given the similarities and differences between ISL and DMF, further exploration of these mechanisms is warranted to better understand their respective therapeutic potentials. Future research could investigate whether the combined use of ISL and DMF might provide synergistic benefits, leading to the development of more effective treatment strategies for white matter injury in preterm infants and other at-risk populations.

In conclusion, our study demonstrates that ISL effectively ameliorates white matter injury and restores neurobehavioral function in preterm infants by inhibiting neuroinflammation and regulating oligodendrocyte development. These findings provide insights into the potential clinical application of ISL for treating white matter injury and related neurological disorders in preterm infants. Future research should focus on elucidating the precise molecular pathways mediating the effects of ISL and optimizing its therapeutic potential in clinical settings.

## Data Availability

The original contributions presented in the study are included in the supplementary material; further inquiries can be directed to the corresponding authors.

## Supplementary material

SUPPLEMENTARY FIGURE 1 Effect of different concentrations of ISL on LPS-induced systemic inflammation **(A,B)** Quantification of the mRNA level of IL-1β and TNF-α in the brain. n = 3–5 for each group. The results are shown as the mean ± SEM, and analyzed by one-way ANOVA followed by post-hoc Turkey test. **p* < 0.05, ***p* < 0.01, ****p* < 0.001, *****p* < 0.0001 compared with the control group. #*p* < 0.05, ##*p* < 0.01, ###*p* < 0.001, ####*p* < 0.0001 compared with the LPS group. “ns” indicates not significant.

SUPPLEMENTARY FIGURE 2 ISL inhibits microglial activation and pro-inflammatory gene expression in vitro **(A)** Effects of different concentrations of ISL on microglial cell viability (n = 6). **(B)** Primary microglia were treated with LPS and ISL for 24 h, and Iba1 were stained (green). **(C,D)** Quantification of the mRNA level of IL-1β and TNF-α (n = 3–4). Scale bar = 20 μm. The results are shown as the mean ± SEM, and analyzed by one-way ANOVA followed by post-hoc Turkey test. **p* < 0.05, ***p* < 0.01, ****p* < 0.001, *****p* < 0.0001 compared with the control group. #*p* < 0.05, ##*p* < 0.01, ###*p* < 0.001, ####*p* < 0.0001 compared with the LPS group. “ns” indicates not significant.
