# Supplementary material for: Isoliquiritigenin ameliorates abnormal oligodendrocyte development and behavior disorders induced by white matter injury
Source: Front Pharmacol. 2024 Sep 11;15:1473019. doi: 10.3389/fphar.2024.1473019 (PMC11423201; doi:10.3389/fphar.2024.1473019)
Supplement: Supplementary file 1 [file Table1.DOCX]

Supplementary Table1. ISL Target

| **Number** | **Target** |
| --- | --- |
| 1 | AKR1B1 |
| 2 | CHRNA7 |
| 3 | EGFR |
| 4 | TERT |
| 5 | ABCG2 |
| 6 | APP |
| 7 | CYP19A1 |
| 8 | MAOA |
| 9 | F3 |
| 10 | NOS2 |
| 11 | SNCA |
| 12 | ALDH2 |
| 13 | KCNA3 |
| 14 | BACE1 |
| 15 | ALOX5 |
| 16 | PTGS2 |
| 17 | MAOB |
| 18 | PDPK1 |
| 19 | ABCB1 |
| 20 | ESR1 |
| 21 | ESR2 |
| 22 | ACHE |
| 23 | TLR9 |
| 24 | HSD17B2 |
| 25 | HSD17B1 |
| 26 | PDE4D |
| 27 | TUBB1 |
| 28 | KCNMA1 |
| 29 | PTPRS |
| 30 | AMY1A |
| 31 | GRK6 |
| 32 | CTSL |
| 33 | IGF1R |
| 34 | PTPN1 |
| 35 | NOX4 |
| 36 | XDH |
| 37 | FLT3 |
| 38 | CCNB3 |
| 39 | CDK6 |
| 40 | ABCC1 |
| 41 | CSNK2A1 |
| 42 | CFTR |
| 43 | CYP1B1 |
| 44 | TNKS2 |
| 45 | TNKS |
| 46 | ALOX12 |
| 47 | STS |
| 48 | DHODH |
| 49 | FNTA FNTB |
| 50 | TYR |
| 51 | AHR |
| 52 | IGFBP3 |
| 53 | PIM1 |
| 54 | CBR1 |
| 55 | AKR1C3 |
| 56 | KDM4E |
| 57 | ALOX15 |
| 58 | CDK1 |
| 59 | GLO1 |
| 60 | MMP12 |
| 61 | CD38 |
| 62 | TOP1 |
| 63 | ARG1 |
| 64 | GSTA1 |
| 65 | SLC22A12 |
| 66 | CDK2 |
| 67 | MGAM |
| 68 | ESRRB |
| 69 | MAPK14 |
| 70 | BCHE |
| 71 | ESR1 |
| 72 | CDK5R1 |
| 73 | GSTP1 |
| 74 | HCK |
| 75 | CES1 |
| 76 | PIM1 |
| 77 | PDE5A |
| 78 | CA2 |
| 79 | CDK2 |
| 80 | ESR2 |
| 81 | LTA4H |
| 82 | MAPK10 |
| 83 | CHEK1 |
| 84 | TTR |
| 85 | AURKA |
| 86 | MAOB |
| 87 | AR |
| 88 | PDPK1 |
| 89 | HSPA8 |
| 90 | EGFR |
| 91 | FGFR1 |
| 92 | BCAT2 |
| 93 | FNTA |
| 94 | CFB |
| 95 | PGR |
| 96 | AMY1A |
| 97 | AMY1B |
| 98 | AMY1C |
| 99 | BACE1 |
| 100 | ANG |
| 101 | NQO1 |
| 102 | CDK6 |
| 103 | PTPN1 |
| 104 | GSK3B |
| 105 | MIF |
| 106 | DAPK1 |
| 107 | F2 |
| 108 | MAPK8 |
| 109 | AKR1B1 |
| 110 | DHFR |
| 111 | HSP90AA1 |
| 112 | CCNA2 |
| 113 | MTHFD1 |
| 114 | CHIT1 |
| 115 | PNP |
| 116 | IMPA1 |
| 117 | HSD17B1 |
| 118 | PDE4B |
| 119 | MMP8 |
| 120 | PRKACA |
| 121 | KDR |
| 122 | KIF11 |
| 123 | MTAP |
| 124 | AMD1 |
| 125 | SHBG |
| 126 | SRC |
| 127 | PYGL |
| 128 | PLK1 |
| 129 | NOS3 |
| 130 | PDE4D |
| 131 | CSNK1G2 |
| 132 | UCK2 |
| 133 | PCK1 |
| 134 | HSD11B1 |
| 135 | PDE3B |
| 136 | ANXA5 |
| 137 | ALDH2 |
| 138 | PPARG |
| 139 | SOD2 |
| 140 | ALB |
| 141 | ESRRG |
| 142 | AHCY |
| 143 | YARS1 |
| 144 | ISG20 |
| 145 | ADAM17 |
| 146 | F7 |
| 147 | PARP1 |
| 148 | EPHX2 |
| 149 | DPP4 |
| 150 | CBR1 |
| 151 | IMPDH2 |
| 152 | LGALS7 |
| 153 | LGALS7B |
| 154 | CDA |
| 155 | AMY2A |
| 156 | NQO2 |
| 157 | HK1 |
| 158 | F10 |
| 159 | DCK |
| 160 | REG1A |
| 161 | PNMT |
| 162 | PDHB |
| 163 | FGFR2 |
| 164 | TNK2 |
| 165 | AKR1C3 |
| 166 | GSR |
| 167 | PAH |
| 168 | CASP3 |
| 169 | RAC2 |
| 170 | ADH5 |
| 171 | SORD |
| 172 | ABO |
| 173 | SULT2A1 |
| 174 | SYK |
| 175 | AKR1C1 |
| 176 | REN |
| 177 | RHOA |
| 178 | FHIT |
| 179 | IGF1 |
| 180 | IGF1R |
| 181 | TGFBR1 |
| 182 | HDAC8 |
| 183 | HMGCR |
| 184 | MMP7 |
| 185 | BIRC7 |
| 186 | NOS2 |
| 187 | PADI4 |
| 188 | DHODH |
| 189 | THRB |
| 190 | LCK |
| 191 | ACP3 |
| 192 | ITK |
| 193 | MMP3 |
| 194 | PLAU |
| 195 | RNASE4 |
| 196 | THRB |
| 197 | RNASE3 |
| 198 | MMP12 |
| 199 | TYMS |
| 200 | CCL5 |
| 201 | CYP2C9 |
| 202 | SSE1 |
| 203 | ELANE |
| 204 | EPHB4 |
| 205 | ALDOA |
| 206 | NR1H2 |
| 207 | TPI1 |
| 208 | ABO |
| 209 | MET |
| 210 | UMPS |
| 211 | NR3C2 |
| 212 | GSTA1 |
| 213 | ARSA |
| 214 | NR1H3 |
| 215 | ERBB4 |
| 216 | CTSS |
| 217 | B3GAT1 |
| 218 | BST1 |
| 219 | PDK2 |
| 220 | SELP |
| 221 | JAK3 |
| 222 | F11 |
| 223 | BLVRB |
| 224 | FECH |
| 225 | PAK6 |
| 226 | LGALS2 |
| 227 | ARHGAP1 |
| 228 | CD1A |
| 229 | MMP13 |
| 230 | PPARA |
| 231 | IL2 |
| 232 | DTYMK |
| 233 | THRA |
| 234 | NR1H4 |
| 235 | SULT2B1 |
| 236 | RXRA |
| 237 | FABP6 |
| 238 | TPH1 |
| 239 | XIAP |
| 240 | ADK |
| 241 | MAPKAPK2 |
| 242 | MAN1B1 |
| 243 | MMP9 |
| 244 | TK1 |
| 245 | CLK1 |
| 246 | PAPSS1 |
| 247 | SERPINA1 |
| 248 | FKBP1A |
| 249 | GPI |
| 250 | CTSK |
| 251 | JAK2 |
| 252 | CBS |
| 253 | LGALS3 |
| 254 | CTSF |
| 255 | ABL1 |
| 256 | NR1I2 |
| 257 | RAB11A |
| 258 | S100A9 |
| 259 | RAB5A |
| 260 | SULT1E1 |
| 261 | SDS |
| 262 | PLA2G2A |
| 263 | ACE |
| 264 | GSTT2B |
| 265 | ARG1 |
| 266 | PPCDC |
| 267 | APRT |
| 268 | MMP2 |
| 269 | PITPNA |
| 270 | TEK |
| 271 | OTC |
| 272 | ACADM |
| 273 | KIT |
| 274 | FABP4 |
| 275 | LYZ |
| 276 | CRABP2 |
| 277 | TGM3 |
| 278 | SETD7 |
| 279 | CTSG |
| 280 | HSPA1A |
| 281 | HSPA1B |
| 282 | IMPDH1 |
| 283 | NMNAT1 |
| 284 | HAGH |
| 285 | HRAS |
| 286 | HADH |
| 287 | SRM |
| 288 | VDR |
| 289 | NR3C1 |
| 290 | OAT |
| 291 | HEXB |
| 292 | CLEC4M |
| 293 | GART |
| 294 | HNF4G |
| 295 | EPHA2 |
| 296 | AKR1C2 |
| 297 | ZAP70 |
| 298 | TPSB2 |
| 299 | ARF4 |
| 300 | BHMT |
| 301 | MME |
| 302 | MAPK1 |
| 303 | STAT1 |
| 304 | DPEP1 |
| 305 | CDC42 |
| 306 | FDPS |
| 307 | FOLH1 |
| 308 | RARA |
| 309 | GNPDA1 |
| 310 | GALE |
| 311 | HMOX1 |
| 312 | CD209 |
| 313 | EIF4E |
| 314 | LCN2 |
| 315 | CANT1 |
| 316 | ACAT1 |
| 317 | PIK3R1 |
| 318 | HINT1 |
| 319 | SHMT1 |
| 320 | MMP16 |
| 321 | NNT |
| 322 | HNMT |
| 323 | MMP1 |
| 324 | GSTM2 |
| 325 | CMA1 |
| 326 | INSR |
| 327 | GCK |
| 328 | RARG |
| 329 | ARL5A |
| 330 | ITPKA |
| 331 | SELE |
| 332 | GSTO1 |
| 333 | Rheb |
| 334 | KAT2B |
| 335 | GP1BA |
| 336 | PPP1CC |
| 337 | TGFB2 |
| 338 | MAP2K1 |
| 339 | FKBP1B |
| 340 | GSTM1 |
| 341 | GSTA3 |
| 342 | HPRT1 |
| 343 | NT5M |
| 344 | CTSB |
| 345 | ATIC |
| 346 | ADAM33 |
| 347 | RAF1 |
| 348 | GLO1 |
| 349 | RNASE2 |
| 350 | NMNAT3 |
| 351 | G6PD |
| 352 | PKLR |
| 353 | GMPR |
| 354 | DDX39B |
| 355 | UAP1 |
| 356 | FABP3 |
| 357 | ERI1 |
| 358 | DOT1L |
| 359 | CASP1 |
| 360 | SEC14L2 |
| 361 | TRDMT1 |
| 362 | WARS1 |
