# Supplementary figures and images for: Isoliquiritigenin ameliorates abnormal oligodendrocyte development and behavior disorders induced by white matter injury
Source: Front Pharmacol. 2024 Sep 11;15:1473019. doi: 10.3389/fphar.2024.1473019 (PMC11423201; doi:10.3389/fphar.2024.1473019)

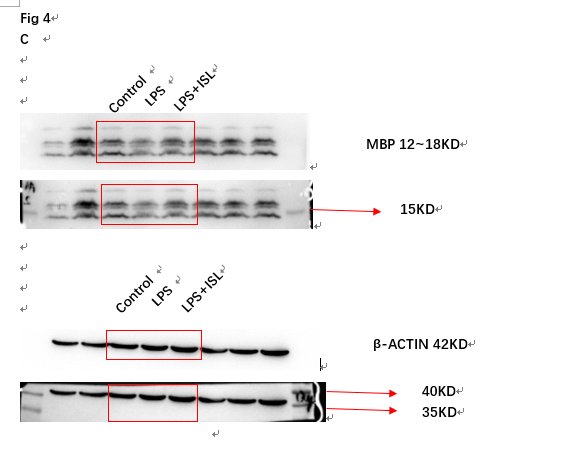

Supplement: Supplementary file 2 [file DataSheet1.zip › Image 1.PNG]

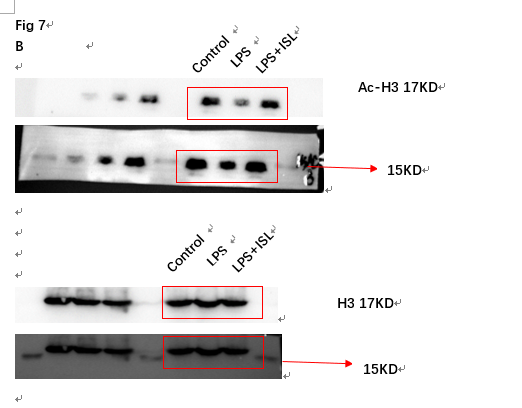

Supplement: Supplementary file 2 [file DataSheet1.zip › Image 2.PNG]

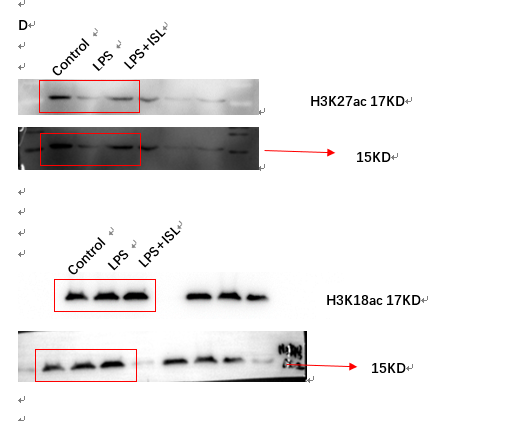

Supplement: Supplementary file 2 [file DataSheet1.zip › Image 3.PNG]

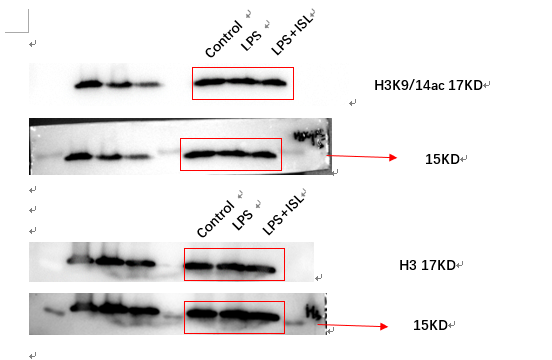

Supplement: Supplementary file 2 [file DataSheet1.zip › Image 4.PNG]

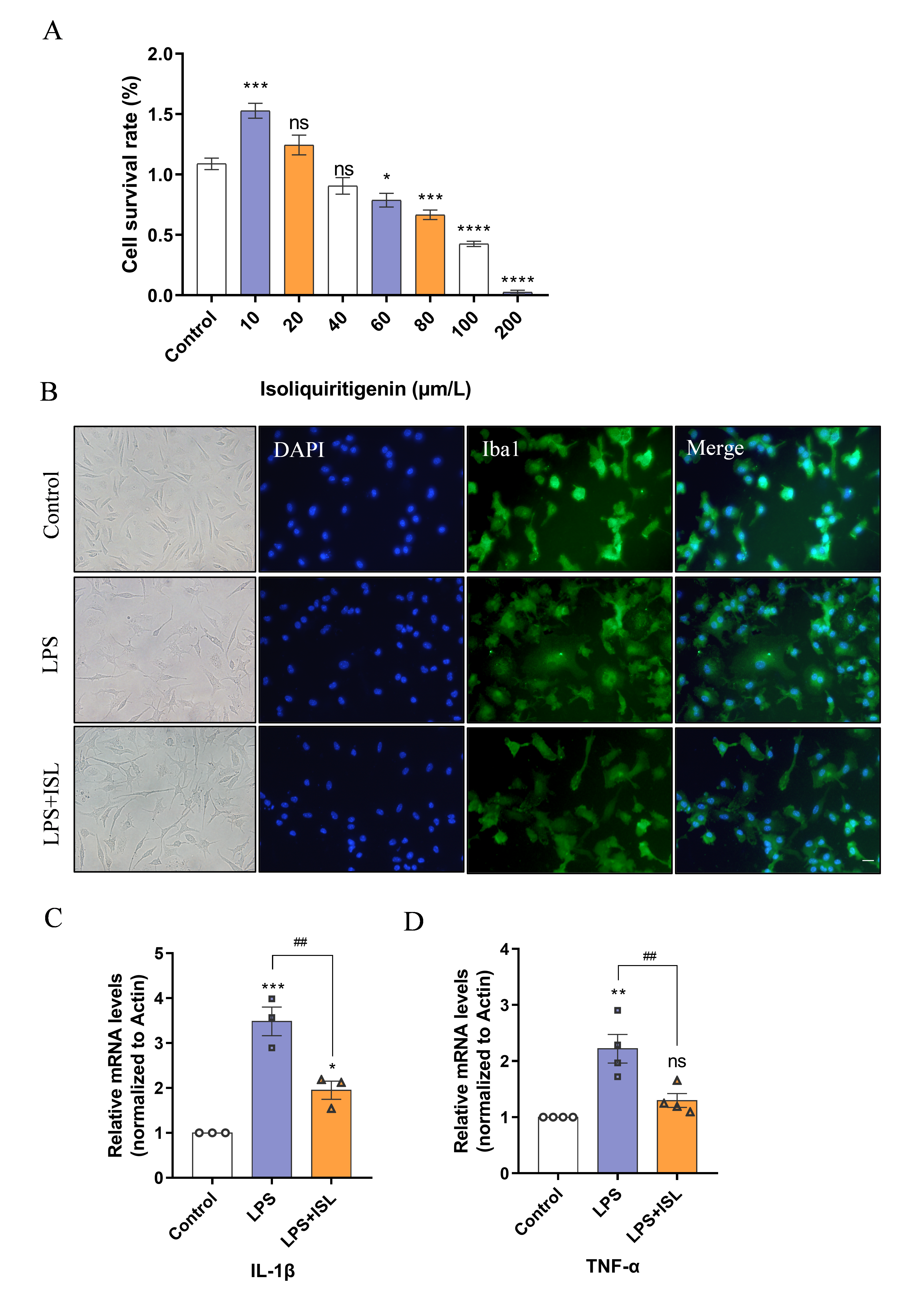

Supplement: Supplementary file 3 [file Image2.tif]

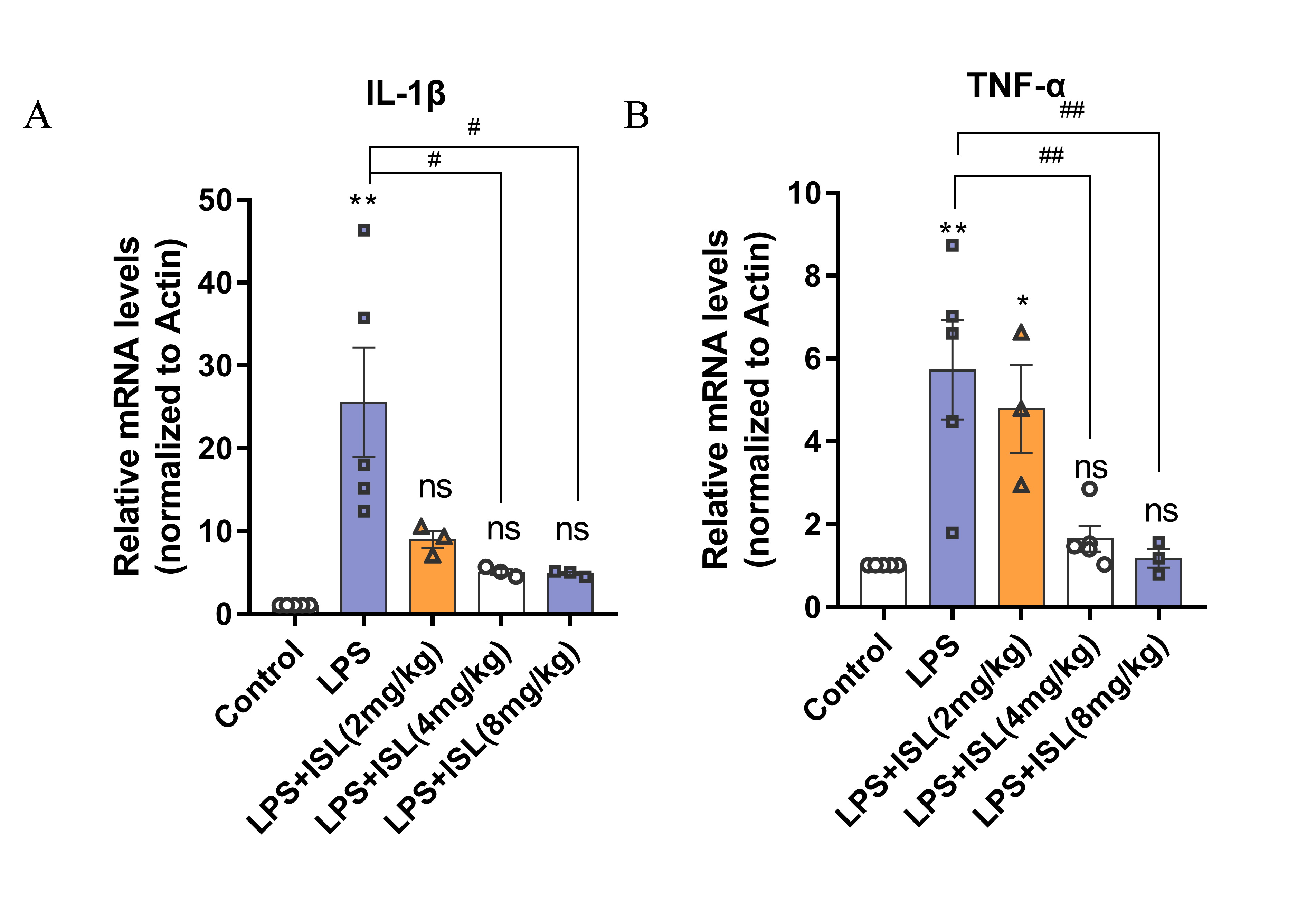

Supplement: Supplementary file 4 [file Image1.tif]
