# Supplementary material for: Isoliquiritigenin ameliorates abnormal oligodendrocyte development and behavior disorders induced by white matter injury
Source: Front Pharmacol. 2024 Sep 11;15:1473019. doi: 10.3389/fphar.2024.1473019 (PMC11423201; doi:10.3389/fphar.2024.1473019)
Supplement: Supplementary file 5 [file Table2.DOCX]

| Supplementary Table2. WMI Target | |
| --- | --- |
| **Number** | **Target** |
| 1 | EIF2B5 |
| 2 | EIF2B4 |
| 3 | EIF2B2 |
| 4 | EIF2B3 |
| 5 | EIF2B1 |
| 6 | GTF3C2-AS2 |
| 7 | PDGFRB |
| 8 | CLCN2 |
| 9 | GAD1 |
| 10 | TP53 |
| 11 | DDB1 |
| 12 | ATM |
| 13 | PRKAG2 |
| 14 | DDB2 |
| 15 | BRCA1 |
| 16 | ANKLE2 |
| 17 | ERCC2 |
| 18 | AARS2 |
| 19 | XPC |
| 20 | APOE |
| 21 | GFAP |
| 22 | HPDL |
| 23 | NOTCH3 |
| 24 | XPA |
| 25 | MDC1 |
| 26 | TNF |
| 27 | PARP1 |
| 28 | ANXA11 |
| 29 | IL6 |
| 30 | RAB11B |
| 31 | ARSA |
| 32 | POGZ |
| 33 | KRT4 |
| 34 | GALC |
| 35 | CHEK2 |
| 36 | GADD45A |
| 37 | BDNF-AS |
| 38 | BRCA2 |
| 39 | RAD51 |
| 40 | KARS1 |
| 41 | CSF1R |
| 42 | NBN |
| 43 | POLR3A |
| 44 | TREX1 |
| 45 | POLG |
| 46 | PLP1 |
| 47 | MAPT |
| 48 | ASPA |
| 49 | ATR |
| 50 | COL4A1 |
| 51 | CDKN1A |
| 52 | ATRIP |
| 53 | AARS1 |
| 54 | CHEK1 |
| 55 | PTEN |
| 56 | MTHFR |
| 57 | TGFB1 |
| 58 | XRCC1 |
| 59 | PSAP |
| 60 | DDIT3 |
| 61 | FANCD2 |
| 62 | HEPACAM |
| 63 | MTOR |
| 64 | ABCC9 |
| 65 | DARS2 |
| 66 | PNKP |
| 67 | LAMA2 |
| 68 | OGG1 |
| 69 | LMNA |
| 70 | HLA-DRB1 |
| 71 | PPARG |
| 72 | PCNA |
| 73 | PIK3CA |
| 74 | H19 |
| 75 | AIFM1 |
| 76 | IL1B |
| 77 | H2AX |
| 78 | HTRA1 |
| 79 | CBS |
| 80 | ERCC4 |
| 81 | SOD1 |
| 82 | ABCD1 |
| 83 | IFNG |
| 84 | GJC2 |
| 85 | IL10 |
| 86 | MT-TL1 |
| 87 | SOD2-OT1 |
| 88 | TP53BP1 |
| 89 | ERCC6 |
| 90 | CDKN2A |
| 91 | CNP |
| 92 | PRKDC |
| 93 | TTN |
| 94 | SPG11 |
| 95 | CREBBP |
| 96 | MDM2 |
| 97 | MBP |
| 98 | CAT |
| 99 | SAMHD1 |
| 100 | RPA1 |
| 101 | INS |
| 102 | LINC01672 |
| 103 | ERCC1 |
| 104 | CRP |
| 105 | MEG3 |
| 106 | KCNQ1 |
| 107 | GCDH |
| 108 | RRM2B |
| 109 | IFIH1 |
| 110 | ACE |
| 111 | MLH1 |
| 112 | FANCI |
| 113 | ERCC3 |
| 114 | TMX2-CTNND1 |
| 115 | EGFR |
| 116 | BCL2 |
| 117 | ERCC5 |
| 118 | SIRT1 |
| 119 | PSEN1 |
| 120 | VCP |
| 121 | TERT |
| 122 | GBA1 |
| 123 | APEX1 |
| 124 | POLR1C |
| 125 | BDNF |
| 126 | EIF2AK2 |
| 127 | GSTM1 |
| 128 | ABL1 |
| 129 | KRAS |
| 130 | MYH7 |
| 131 | BRAF |
| 132 | SCN5A |
| 133 | MC1R |
| 134 | RNASEH2C |
| 135 | POLR3B |
| 136 | CCND1 |
| 137 | EP300 |
| 138 | MIR21 |
| 139 | RPA2 |
| 140 | STAT3 |
| 141 | RNASEH2B |
| 142 | FMR1 |
| 143 | JUP |
| 144 | RFWD3 |
| 145 | ADAR |
| 146 | WRN |
| 147 | CTNNB1 |
| 148 | TYR |
| 149 | LEP |
| 150 | HRAS |
| 151 | SMAD5-AS1 |
| 152 | HFE |
| 153 | CASP3 |
| 154 | CERNA3 |
| 155 | ALB |
| 156 | EARS2 |
| 157 | KIT |
| 158 | SLC1A2 |
| 159 | GSTP1 |
| 160 | TTN-AS1 |
| 161 | HEXA |
| 162 | MIR155 |
| 163 | HYCC1 |
| 164 | LPL |
| 165 | MPO |
| 166 | RBBP8 |
| 167 | ABCG2 |
| 168 | RAD17 |
| 169 | TNNT2 |
| 170 | TMEM63A |
| 171 | HLA-DQB1 |
| 172 | MAP1B |
| 173 | CXCL8 |
| 174 | AKT1 |
| 175 | FANCA |
| 176 | APP |
| 177 | BAX |
| 178 | DEGS1 |
| 179 | ADIPOQ |
| 180 | RANBP2 |
| 181 | SOX10 |
| 182 | RNASEH2A |
| 183 | CCL2 |
| 184 | MYBPC3 |
| 185 | BRIP1 |
| 186 | NRAS |
| 187 | XRCC5 |
| 188 | TP73 |
| 189 | POLGARF |
| 190 | MCPH1 |
| 191 | HIKESHI |
| 192 | COL4A2 |
| 193 | POLH |
| 194 | MAG |
| 195 | MALAT1 |
| 196 | GJB1 |
| 197 | LMNB1 |
| 198 | MSH2 |
| 199 | SPG7 |
| 200 | IGF1 |
| 201 | PAFAH1B1 |
| 202 | DRAM1 |
| 203 | MRE11 |
| 204 | CFTR |
| 205 | GRN |
| 206 | CDK1 |
| 207 | LDLR |
| 208 | MYC |
| 209 | FA2H |
| 210 | FBN1 |
| 211 | PTPN11 |
| 212 | ALMS1 |
| 213 | SLC26A4 |
| 214 | MIR17 |
| 215 | ACTC1 |
| 216 | PAH |
| 217 | NOS2 |
| 218 | PML |
| 219 | CDKN2B-AS1 |
| 220 | COMT |
| 221 | GSTT1 |
| 222 | TSC1 |
| 223 | PMS2 |
| 224 | MLC1 |
| 225 | RAD50 |
| 226 | ATRIP-TREX1 |
| 227 | SOD2 |
| 228 | TSEN54 |
| 229 | IL1A |
| 230 | DCX |
| 231 | VDR |
| 232 | CFH |
| 233 | PEX6 |
| 234 | COL1A1 |
| 235 | RAD23B |
| 236 | APOB |
| 237 | HLA-B |
| 238 | NPM1 |
| 239 | MGMT |
| 240 | FAS |
| 241 | TYMP |
| 242 | CTLA4 |
| 243 | NLRP3 |
| 244 | PON1 |
| 245 | NOS3 |
| 246 | MT-CYB |
| 247 | DRAM2 |
| 248 | ACOX1 |
| 249 | MIR125A |
| 250 | RNF216 |
| 251 | NDUFA2 |
| 252 | USP7 |
| 253 | BARD1 |
| 254 | MFN2 |
| 255 | RAD9A |
| 256 | MIR34A |
| 257 | TLR4 |
| 258 | PARP2 |
| 259 | PRDM16 |
| 260 | SPG21 |
| 261 | LIG3 |
| 262 | RB1 |
| 263 | XRCC2 |
| 264 | CDK2 |
| 265 | PALB2 |
| 266 | CYP1A1 |
| 267 | MLH3 |
| 268 | GLA |
| 269 | PPP1R15A |
| 270 | TSC2 |
| 271 | RNF168 |
| 272 | VEGFA |
| 273 | EDNRB |
| 274 | HMOX1 |
| 275 | AGT |
| 276 | TOPBP1 |
| 277 | HBB |
| 278 | MT-ND5 |
| 279 | MUTYH |
| 280 | PDGFRA |
| 281 | CASP9 |
| 282 | FANCC |
| 283 | GAS5 |
| 284 | RAC1 |
| 285 | NODAL |
| 286 | RAD51C |
| 287 | RPS27A |
| 288 | TNNI3 |
| 289 | ALDH3A2 |
| 290 | MIR223 |
| 291 | GLI2 |
| 292 | ESR1 |
| 293 | CACNA1A |
| 294 | RNF8 |
| 295 | ICAM1 |
| 296 | ERCC8 |
| 297 | SQSTM1 |
| 298 | OPA1 |
| 299 | XRCC6 |
| 300 | MEFV |
| 301 | SMARCA4 |
| 302 | TM4SF20 |
| 303 | FANCG |
| 304 | MSH6 |
| 305 | U2AF2 |
| 306 | FLNA |
| 307 | PRF1 |
| 308 | XRCC4 |
| 309 | RAB33A |
| 310 | PTPN22 |
| 311 | ALDH18A1 |
| 312 | CYP7B1 |
| 313 | YWHAE |
| 314 | NARS2 |
| 315 | CYCS |
| 316 | E2F1 |
| 317 | MCOLN1 |
| 318 | PEX16 |
| 319 | PPARGC1A |
| 320 | CEBPA |
| 321 | POLR3K |
| 322 | SETX |
| 323 | FEN1 |
| 324 | GJB2 |
| 325 | PRNP |
| 326 | LSM11 |
| 327 | TWNK |
| 328 | LIG4 |
| 329 | POMGNT1 |
| 330 | TERC |
| 331 | CD36 |
| 332 | NFE2L2 |
| 333 | PLK1 |
| 334 | AR |
| 335 | SNCA |
| 336 | FASLG |
| 337 | XIST |
| 338 | TUBB4A |
| 339 | MAPK1 |
| 340 | MIR126 |
| 341 | DNMT3A |
| 342 | ARID1A |
| 343 | ERBB2 |
| 344 | HSD17B4 |
| 345 | MITF |
| 346 | LOC126862402 |
| 347 | ISCA2 |
| 348 | MAPK14 |
| 349 | NORAD |
| 350 | MTFMT |
| 351 | AQP4 |
| 352 | RLBP1 |
| 353 | ARSB |
| 354 | NOTCH1 |
| 355 | GLUL |
| 356 | NFKB1 |
| 357 | PEX10 |
| 358 | MBL2 |
| 359 | PRKN |
| 360 | PVT1 |
| 361 | CYP2E1 |
| 362 | AIMP1 |
| 363 | FKTN |
| 364 | CYP1B1 |
| 365 | CDKN1B |
| 366 | HMGB1 |
| 367 | STN1 |
| 368 | HOTAIR |
| 369 | PYCR2 |
| 370 | FBXW7 |
| 371 | NDUFAF2 |
| 372 | SERPINA1 |
| 373 | MIR20A |
| 374 | CREB1 |
| 375 | EMSLR |
| 376 | C9orf72 |
| 377 | CSF3 |
| 378 | SMARCAL1 |
| 379 | EPHX1 |
| 380 | MOG |
| 381 | GPT |
| 382 | BMP2 |
| 383 | MIR27A |
| 384 | TF |
| 385 | SLX4 |
| 386 | TUBA1A |
| 387 | PEX1 |
| 388 | HP |
| 389 | WT1 |
| 390 | SELENOI |
| 391 | VPS11 |
| 392 | POLD1 |
| 393 | PI4KA |
| 394 | ADA |
| 395 | LRP5 |
| 396 | IKBKG |
| 397 | MMP9 |
| 398 | RNF220 |
| 399 | CD4 |
| 400 | YARS1 |
| 401 | TARDBP |
| 402 | VWF |
| 403 | JAK2 |
| 404 | APOA1 |
| 405 | CTSD |
| 406 | TUG1 |
| 407 | NEFL |
| 408 | MT-CO2 |
| 409 | SCN1A |
| 410 | GRIK2 |
| 411 | RECQL4 |
| 412 | DARS1 |
| 413 | IL17A |
| 414 | STAT1 |
| 415 | TREM2 |
| 416 | DINOL |
| 417 | MIR146A |
| 418 | MT-ND1 |
| 419 | AKT3 |
| 420 | LSM7 |
| 421 | CPT2 |
| 422 | ZFYVE26 |
| 423 | SLC6A4 |
| 424 | DNM1L |
| 425 | AP5Z1 |
| 426 | TOP1 |
| 427 | OCA2 |
| 428 | SLC13A5 |
| 429 | FKRP |
| 430 | DDHD2 |
| 431 | SNORD118 |
| 432 | MPZ |
| 433 | CDC42 |
| 434 | IBA57 |
| 435 | MIR142 |
| 436 | CTC1 |
| 437 | VCAN |
| 438 | IL4 |
| 439 | MIR145 |
| 440 | DNMT1 |
| 441 | TUBB |
| 442 | ABCA4 |
| 443 | IDH1 |
| 444 | RELN |
| 445 | TYMS |
| 446 | IL1RN |
| 447 | CACNA1C |
| 448 | TOP2A |
| 449 | ACKR1 |
| 450 | FANCM |
| 451 | S100B |
| 452 | FANCB |
| 453 | PTCH1 |
| 454 | RUNX1 |
| 455 | ABCB1 |
| 456 | B2M |
| 457 | CDK4 |
| 458 | MHRT |
| 459 | IL18 |
| 460 | FLT3 |
| 461 | GAA |
| 462 | BMP4 |
| 463 | KAT5 |
| 464 | CLN5 |
| 465 | CUL4B |
| 466 | NAGLU |
| 467 | HTT |
| 468 | PAX6 |
| 469 | TTR |
| 470 | ADRB2 |
| 471 | COL4A4 |
| 472 | CASP8 |
| 473 | RNASET2 |
| 474 | BLM |
| 475 | TNFRSF1A |
| 476 | ATXN2 |
| 477 | HNRNPK |
| 478 | FGFR3 |
| 479 | PIK3R1 |
| 480 | MAPK8 |
| 481 | SDHB |
| 482 | SLC17A5 |
| 483 | ODC1 |
| 484 | SP1 |
| 485 | EGF |
| 486 | ABCG8 |
| 487 | SHANK3 |
| 488 | MIRLET7C |
| 489 | NQO1 |
| 490 | PAX3 |
| 491 | ELANE |
| 492 | POT1 |
| 493 | PRKCD |
| 494 | LAMB1 |
| 495 | JAG1 |
| 496 | NEAT1 |
| 497 | MIR221 |
| 498 | FOXG1 |
| 499 | DISC1 |
| 500 | CTNS |
| 501 | RAD52 |
| 502 | RNU7-1 |
| 503 | SPRTN |
| 504 | ALDH2 |
| 505 | SNAI2 |
| 506 | MBTPS2 |
| 507 | SFSWAP |
| 508 | HLA-DQA1 |
| 509 | G6PD |
| 510 | SCO2 |
| 511 | JUN |
| 512 | GSK3B |
| 513 | PGR-AS1 |
| 514 | CP |
| 515 | F2 |
| 516 | IL2 |
| 517 | LINC02605 |
| 518 | FOXP3 |
| 519 | SMC1A |
| 520 | SLC2A1 |
| 521 | CUL4A |
| 522 | MECP2 |
| 523 | GGT1 |
| 524 | CDH1 |
| 525 | MEN1 |
| 526 | SZT2 |
| 527 | DMD |
| 528 | ATRX |
| 529 | GFM2 |
| 530 | MIR210 |
| 531 | SHH |
| 532 | FGFR2 |
| 533 | TUBB2A |
| 534 | NPC1 |
| 535 | ABCG5 |
| 536 | HSPD1 |
| 537 | UGT1A1 |
| 538 | SERPINE1 |
| 539 | AGTR1 |
| 540 | NF1 |
| 541 | MMP1 |
| 542 | CLN3 |
| 543 | PTGS2 |
| 544 | CDK5 |
| 545 | FANCL |
| 546 | POLI |
| 547 | STING1 |
| 548 | CASQ2 |
| 549 | AMACR |
| 550 | MAD2L2 |
| 551 | GTF2H5 |
| 552 | TMCO1 |
| 553 | PRPH2 |
| 554 | SPP1 |
| 555 | MTRR |
| 556 | CHMP1A |
| 557 | EIF2AK3 |
| 558 | MIR140 |
| 559 | ADORA2A |
| 560 | BCAP31 |
| 561 | MYD88 |
| 562 | CKB |
| 563 | COL4A5 |
| 564 | NOD2 |
| 565 | POLR2A |
| 566 | DLEU2 |
| 567 | CDK6 |
| 568 | GJA1 |
| 569 | NOTCH2NLC |
| 570 | MMACHC |
| 571 | IKZF1 |
| 572 | STAT2 |
| 573 | TPP1 |
| 574 | IDH2 |
| 575 | COL1A2 |
| 576 | ABHD16A |
| 577 | PEX12 |
| 578 | ADGRG1 |
| 579 | KAT6B |
| 580 | FTO |
| 581 | MORC2 |
| 582 | FZR1 |
| 583 | ATP7B |
| 584 | CEP290 |
| 585 | IL13 |
| 586 | NTRK2 |
| 587 | TUBB3 |
| 588 | APC |
| 589 | MIR22 |
| 590 | CYP27A1 |
| 591 | DAG1 |
| 592 | CYP3A4 |
| 593 | LARGE1 |
| 594 | WWOX |
| 595 | THBD |
| 596 | FBXL4 |
| 597 | FANCE |
| 598 | DYNC1H1 |
| 599 | TBCK |
| 600 | LTA |
| 601 | MIR23A |
| 602 | PIK3C2A |
| 603 | HDAC1 |
| 604 | HSPB1 |
| 605 | ACTA2 |
| 606 | DPM3 |
| 607 | EXO1 |
| 608 | PRPS1 |
| 609 | XDH |
| 610 | DNAH8 |
| 611 | IDS |
| 612 | GSR |
| 613 | NEIL1 |
| 614 | SETD2 |
| 615 | PEX26 |
| 616 | CD44 |
| 617 | GAPDH |
| 618 | CLU |
| 619 | MIRLET7B |
| 620 | ENG |
| 621 | TRA-TGC7-1 |
| 622 | SBDS |
| 623 | FH |
| 624 | LAMP2 |
| 625 | RHOA |
| 626 | NR3C1 |
| 627 | UBA1 |
| 628 | RERE |
| 629 | LIG1 |
| 630 | INSR |
| 631 | CAPN3 |
| 632 | CEBPB |
| 633 | GLB1 |
| 634 | SRC |
| 635 | UBE2T |
| 636 | NOS1 |
| 637 | SLC1A3 |
| 638 | GATA2 |
| 639 | DPYD |
| 640 | FANCF |
| 641 | POMT2 |
| 642 | NDE1 |
| 643 | NAT2 |
| 644 | PINK1 |
| 645 | MIR146B |
| 646 | ENPP1 |
| 647 | MIR29A |
| 648 | PLA2G6 |
| 649 | CSNK2A1 |
| 650 | SURF1 |
| 651 | SETBP1 |
| 652 | DCDC2 |
| 653 | TBK1 |
| 654 | RYR1 |
| 655 | SPAST |
| 656 | HMGCR |
| 657 | SF3B1 |
| 658 | RARA |
| 659 | MIAT |
| 660 | NDUFS1 |
| 661 | FUS |
| 662 | RETN |
| 663 | PEX13 |
| 664 | ARFGEF2 |
| 665 | MIR30A |
| 666 | MT-ND6 |
| 667 | OLIG2 |
| 668 | PRORP |
| 669 | MT-CO3 |
| 670 | SNORD15A |
| 671 | ENTPD1 |
| 672 | TRAPPC11 |
| 673 | SEPSECS |
| 674 | DHCR24 |
| 675 | CYP2D6 |
| 676 | PNPT1 |
| 677 | L1CAM |
| 678 | GPX4 |
| 679 | MIR34C |
| 680 | PPP1R21 |
| 681 | EZH2 |
| 682 | LOC106627981 |
| 683 | TUBB2B |
| 684 | TP63 |
| 685 | ATP1A2 |
| 686 | MIR132 |
| 687 | HGF |
| 688 | HMGCL |
| 689 | TNFRSF1B |
| 690 | CTBP1 |
| 691 | SELE |
| 692 | C11orf65 |
| 693 | PEX19 |
| 694 | HERC2 |
| 695 | DMXL2 |
| 696 | MTR |
| 697 | MT-ND4 |
| 698 | MMP2 |
| 699 | VCAM1 |
| 700 | RYR2 |
| 701 | BCL2L1 |
| 702 | HLA-DPB1 |
| 703 | UBE2A |
| 704 | YY1 |
| 705 | APTX |
| 706 | EFEMP1 |
| 707 | SMARCA2 |
| 708 | SLC25A4 |
| 709 | VIM |
| 710 | JAK1 |
| 711 | NKX6-2 |
| 712 | DEAF1 |
| 713 | CDK7 |
| 714 | C4A |
| 715 | SELP |
| 716 | ADSL |
| 717 | ZEB2 |
| 718 | MIR222 |
| 719 | PIK3CD |
| 720 | IRF4 |
| 721 | SLC35B2 |
| 722 | WNT1 |
| 723 | REV3L |
| 724 | STXBP1 |
| 725 | PSMD3 |
| 726 | TMEM106B |
| 727 | TLR2 |
| 728 | MIR451A |
| 729 | MIF |
| 730 | DNASE1 |
| 731 | RNF2 |
| 732 | CYP2C9 |
| 733 | FGFR1 |
| 734 | BRAT1 |
| 735 | PEX2 |
| 736 | HNF1A |
| 737 | MIR133B |
| 738 | UBE2N |
| 739 | TINF2 |
| 740 | LIPA |
| 741 | NHLRC2 |
| 742 | RTEL1 |
| 743 | RHO |
| 744 | SLC12A2 |
| 745 | PEX11B |
| 746 | GNAS |
| 747 | SLC2A4 |
| 748 | PC |
| 749 | LRRK2 |
| 750 | CETP |
| 751 | RBPJ |
| 752 | MT-CO1 |
| 753 | PLAT |
| 754 | KAT8 |
| 755 | PPT1 |
| 756 | FAR1 |
| 757 | ATF4 |
| 758 | MIR7-3HG |
| 759 | MT-ATP6 |
| 760 | SDHAF1 |
| 761 | EDN3 |
| 762 | MIR203A |
| 763 | CD55 |
| 764 | HLA-A |
| 765 | PURA |
| 766 | MIR320A |
| 767 | SUOX |
| 768 | CCNB1 |
| 769 | COL5A2 |
| 770 | MMP3 |
| 771 | SPTAN1 |
| 772 | HUWE1 |
| 773 | ANXA5 |
| 774 | CHKB-CPT1B |
| 775 | NDUFS4 |
| 776 | MIR16-1 |
| 777 | TCF7L2 |
| 778 | WRAP53 |
| 779 | PPARA |
| 780 | GRIN1 |
| 781 | HPRT1 |
| 782 | KDM1A |
| 783 | SLC6A3 |
| 784 | NUP188 |
| 785 | NPPB |
| 786 | SUMF1 |
| 787 | HLA-C |
| 788 | FOXO3 |
| 789 | CRPPA |
| 790 | TNFAIP3 |
| 791 | F5 |
| 792 | IGFBP3 |
| 793 | CD40LG |
| 794 | MIR27B |
| 795 | SPATA22 |
| 796 | SLC25A20 |
| 797 | FN1 |
| 798 | CHD7 |
| 799 | CXCL12 |
| 800 | TRAPPC9 |
| 801 | MYH9 |
| 802 | KMT2A |
| 803 | IGF2 |
| 804 | PEX5 |
| 805 | TET2 |
| 806 | NOTCH2 |
| 807 | PPP1R15B |
| 808 | PPP3CA |
| 809 | YWHAG |
| 810 | STK11 |
| 811 | FCGR2A |
| 812 | EML1 |
| 813 | MIR143 |
| 814 | ATF2 |
| 815 | ALS2 |
| 816 | MAPK3 |
| 817 | RFC1 |
| 818 | TYRP1 |
| 819 | EPRS1 |
| 820 | ITGAM |
| 821 | AURKA |
| 822 | EDAR |
| 823 | MPL |
| 824 | CST3 |
| 825 | HDAC9 |
| 826 | VCL |
| 827 | MIR494 |
| 828 | IRS1 |
| 829 | MCL1 |
| 830 | PARS2 |
| 831 | LRRC56 |
| 832 | JAK3 |
| 833 | MCM7 |
| 834 | CLN8 |
| 835 | DOCK7 |
| 836 | CCR5 |
| 837 | SF3B2 |
| 838 | WDR26 |
| 839 | CFI |
| 840 | GMPPB |
| 841 | PTPRC |
| 842 | VRK1 |
| 843 | BIN1 |
| 844 | MIR23B |
| 845 | PLAA |
| 846 | MT-TK |
| 847 | CDKN2B |
| 848 | PLG |
| 849 | WARS2 |
| 850 | MAP2K1 |
| 851 | ACP5 |
| 852 | LIPT2 |
| 853 | KMT2D |
| 854 | ATP1A3 |
| 855 | GJB6 |
| 856 | CHD4 |
| 857 | ABCB4 |
| 858 | ABCB11 |
| 859 | RNF113A |
| 860 | MIRLET7D |
| 861 | GBE1 |
| 862 | POMT1 |
| 863 | PLA2G7 |
| 864 | ITGB1 |
| 865 | HNF4A |
| 866 | SMPD1 |
| 867 | HSPA8 |
| 868 | BCL6 |
| 869 | MIR29C |
| 870 | C3 |
| 871 | ATP13A2 |
| 872 | MIRLET7E |
| 873 | SNAP29 |
| 874 | KRT14 |
| 875 | PISD |
| 876 | GNB1 |
| 877 | POLG2 |
| 878 | GATAD1 |
| 879 | REN |
| 880 | ARMS2 |
| 881 | FOXO1 |
| 882 | LIPC |
| 883 | VARS1 |
| 884 | SMAD4 |
| 885 | CDH23 |
| 886 | ATAD3A |
| 887 | SCN2A |
| 888 | DNASE2 |
| 889 | PLEKHG2 |
| 890 | HELLS |
| 891 | TGFBR2 |
| 892 | LOC107133510 |
| 893 | ELOVL1 |
| 894 | PDCD1 |
| 895 | HSPA4 |
| 896 | RXYLT1 |
| 897 | CLN6 |
| 898 | RMRP |
| 899 | ACER3 |
| 900 | MFSD2A |
| 901 | SUPT16H |
| 902 | TUFM |
| 903 | GUSB |
| 904 | GALNT2 |
| 905 | EDN1 |
| 906 | TIMM50 |
| 907 | H3C1 |
| 908 | LINC-ROR |
| 909 | HSP90AA1 |
| 910 | BIRC5 |
| 911 | CXCL10 |
| 912 | ADA2 |
| 913 | MIR93 |
| 914 | RBP4 |
| 915 | DKC1 |
| 916 | ZNF423 |
| 917 | CLCN3 |
| 918 | ACTG1 |
| 919 | VHL |
| 920 | TK2 |
| 921 | CCND2 |
| 922 | ACTB |
| 923 | KIF5A |
| 924 | SMAD3 |
| 925 | SPARC |
| 926 | BABAM2 |
| 927 | PIK3CG |
| 928 | CSF2 |
| 929 | EIF2AK4 |
| 930 | IGF1R |
| 931 | UBQLN4 |
| 932 | EIF2S3 |
| 933 | ABCC8 |
| 934 | SLC25A24 |
| 935 | CBL |
| 936 | DRD2 |
| 937 | KANSL1 |
| 938 | UCP2 |
| 939 | PPFIBP1 |
| 940 | HDAC2 |
| 941 | RAB9B |
| 942 | MEF2C |
| 943 | COL6A2 |
| 944 | MPLKIP |
| 945 | AMPD2 |
| 946 | AIMP2 |
| 947 | MIR15B |
| 948 | NPY |
| 949 | DTL |
| 950 | CXCR4 |
| 951 | CSF1 |
| 952 | BPTF |
| 953 | GM2A |
| 954 | TLR3 |
| 955 | HDAC4 |
| 956 | EIF2S2 |
| 957 | CDKL5 |
| 958 | NDUFV1 |
| 959 | FXN |
| 960 | CRYAB |
| 961 | MDM4 |
| 962 | MYH14 |
| 963 | PHGDH |
| 964 | MIR130A |
| 965 | RUNX2 |
| 966 | ASXL1 |
| 967 | SACS |
| 968 | ANK2 |
| 969 | EPO |
| 970 | RMND1 |
| 971 | GPX1 |
| 972 | MT-TF |
| 973 | TNFRSF11B |
| 974 | REV1 |
| 975 | WARS1 |
| 976 | HACE1 |
| 977 | ITGB2 |
| 978 | HIF1A |
| 979 | SOCS1 |
| 980 | PIK3R2 |
| 981 | USP18 |
| 982 | MIR10B |
| 983 | ASPM |
| 984 | NPPA |
| 985 | ZEB1 |
| 986 | SFTA3 |
| 987 | GCK |
| 988 | CGAS |
| 989 | LOC106099062 |
| 990 | CYP3A5 |
| 991 | SIRT6 |
| 992 | L2HGDH |
| 993 | ENO2 |
| 994 | ACADM |
| 995 | AP4B1 |
| 996 | UVSSA |
| 997 | RARS1 |
| 998 | ZNF335 |
| 999 | TPMT |
| 1000 | GBA2 |
| 1001 | BMP6 |
| 1002 | ABCA1 |
| 1003 | SLC11A1 |
| 1004 | WDR45B |
| 1005 | ELN |
| 1006 | BTD |
| 1007 | KIF2A |
| 1008 | KCNB1 |
| 1009 | CBX3 |
| 1010 | NTHL1 |
| 1011 | CARS1 |
| 1012 | MT-RNR1 |
| 1013 | RTN4R |
| 1014 | TMEM67 |
| 1015 | MIR122 |
| 1016 | TNFRSF10B |
| 1017 | NRG1 |
| 1018 | UGT1A6 |
| 1019 | AP4M1 |
| 1020 | DYRK1A |
| 1021 | MT-TS1 |
| 1022 | GATAD2B |
| 1023 | ERBB3 |
| 1024 | IRF5 |
| 1025 | POMC |
| 1026 | MIR199A1 |
| 1027 | RAF1 |
| 1028 | AUH |
| 1029 | LOC110806262 |
| 1030 | MIR195 |
| 1031 | KITLG |
| 1032 | POLE |
| 1033 | APC2 |
| 1034 | CX3CR1 |
| 1035 | GFM1 |
| 1036 | PRTN3 |
| 1037 | FGF2 |
| 1038 | LYRM7 |
| 1039 | IFNA1 |
| 1040 | PAK1 |
| 1041 | LOC117038795 |
| 1042 | NLRP1 |
| 1043 | C4B |
| 1044 | FDXR |
| 1045 | HSPG2 |
| 1046 | XIAP |
| 1047 | BCR |
| 1048 | NR2F1 |
| 1049 | NHEJ1 |
| 1050 | MET |
| 1051 | ACO2 |
| 1052 | HADHA |
| 1053 | ABCC1 |
| 1054 | NSD2 |
| 1055 | TRPV4 |
| 1056 | FABP4 |
| 1057 | LOC126859784 |
| 1058 | MYCN |
| 1059 | IL1R1 |
| 1060 | TIMP3 |
| 1061 | FBP2 |
| 1062 | KCNH2 |
| 1063 | BEST1 |
| 1064 | WDR62 |
| 1065 | SON |
| 1066 | LRP2 |
| 1067 | PEX14 |
| 1068 | IL5 |
| 1069 | NABP1 |
| 1070 | DES |
| 1071 | SAMD9L |
| 1072 | IKBKB |
| 1073 | ZNF469 |
| 1074 | HTR1A |
| 1075 | MT-ND2 |
| 1076 | IDUA |
| 1077 | MIR204 |
| 1078 | KATNB1 |
| 1079 | KCNJ10 |
| 1080 | ACTL6B |
| 1081 | UNC13D |
| 1082 | CD46 |
| 1083 | RIF1 |
| 1084 | MT-TS2 |
| 1085 | FCGR3B |
| 1086 | RNU4ATAC |
| 1087 | FGB |
| 1088 | MIR342 |
| 1089 | SSBP1 |
| 1090 | COL2A1 |
| 1091 | MOBP |
| 1092 | TBP |
| 1093 | KCNJ11 |
| 1094 | EDNRA |
| 1095 | GRIN2B |
| 1096 | PRIMPOL |
| 1097 | MIR31 |
| 1098 | NPHS1 |
| 1099 | SMARCB1 |
| 1100 | RELA |
| 1101 | TFAM |
| 1102 | CARS2 |
| 1103 | PLOD1 |
| 1104 | PMM2 |
| 1105 | FRAXA |
| 1106 | SDHA |
| 1107 | MIR141 |
| 1108 | BGLAP |
| 1109 | GNA12 |
| 1110 | MT-TH |
| 1111 | TARS1 |
| 1112 | BID |
| 1113 | SYK |
| 1114 | NGF |
| 1115 | SERPINF1 |
| 1116 | SMC3 |
| 1117 | LMBRD2 |
| 1118 | ATN1 |
| 1119 | PPP2R1A |
| 1120 | PDHA1 |
| 1121 | NAE1 |
| 1122 | CASR |
| 1123 | FIG4 |
| 1124 | EEF1A2 |
| 1125 | CD28 |
| 1126 | MT-ND3 |
| 1127 | IKBKE |
| 1128 | BUB1 |
| 1129 | DNAAF3 |
| 1130 | EIF4G1 |
| 1131 | MT-ATP8 |
| 1132 | IL7R |
| 1133 | ANGPT2 |
| 1134 | SMN2 |
| 1135 | MIR24-1 |
| 1136 | PDGFB |
| 1137 | CALR |
| 1138 | TRIM28 |
| 1139 | MPV17 |
| 1140 | CEP164 |
| 1141 | TUBG1 |
| 1142 | DNM1 |
| 1143 | MIR26B |
| 1144 | SELL |
| 1145 | UCA1 |
| 1146 | ADD1 |
| 1147 | MUS81 |
| 1148 | MKI67 |
| 1149 | TRIP12 |
| 1150 | PRKAR1A |
| 1151 | LIF |
| 1152 | CNTN2 |
| 1153 | CTNNA1 |
| 1154 | MIR373 |
| 1155 | PLEC |
| 1156 | CASP1 |
| 1157 | GALNS |
| 1158 | AHR |
| 1159 | LYST |
| 1160 | ACE2 |
| 1161 | SLC12A6 |
| 1162 | HNRNPU |
| 1163 | MIR200A |
| 1164 | CYP1A2 |
| 1165 | MT-TP |
| 1166 | MB |
| 1167 | APOH |
| 1168 | MIR128-1 |
| 1169 | BAP1 |
| 1170 | FADD |
| 1171 | SIX3 |
| 1172 | DNAJC3 |
| 1173 | COL5A1 |
| 1174 | MUC1 |
| 1175 | RBX1 |
| 1176 | TH |
| 1177 | SFN |
| 1178 | SREBF1 |
| 1179 | CRTAP |
| 1180 | NAXE |
| 1181 | PIGA |
| 1182 | IL4R |
| 1183 | CYP19A1 |
| 1184 | CASP10 |
| 1185 | LBR |
| 1186 | UGT1A7 |
| 1187 | SCN8A |
| 1188 | DYSF |
| 1189 | WFS1 |
| 1190 | GTF2E2 |
| 1191 | MYO7A |
| 1192 | AP3B1 |
| 1193 | CNTNAP2 |
| 1194 | FOXC1 |
| 1195 | THPO |
| 1196 | WAS |
| 1197 | CUBN |
| 1198 | AP4S1 |
| 1199 | MIR193A |
| 1200 | LOC129994126 |
| 1201 | SRSF2 |
| 1202 | PPIB |
| 1203 | TMX2 |
| 1204 | HNF1B |
| 1205 | FCSK |
| 1206 | PARK7 |
| 1207 | CCNE1 |
| 1208 | FOS |
| 1209 | APOC3 |
| 1210 | LEPR |
| 1211 | PMP22 |
| 1212 | TCF4 |
| 1213 | UNG |
| 1214 | SCARNA5 |
| 1215 | ADAT3 |
| 1216 | DCT |
| 1217 | DHX9 |
| 1218 | NDUFA6 |
| 1219 | FIP1L1 |
| 1220 | PSMA7 |
| 1221 | COL3A1 |
| 1222 | MME |
| 1223 | SLC25A1 |
| 1224 | ETFDH |
| 1225 | MOCS2 |
| 1226 | SP7 |
| 1227 | H2AC20 |
| 1228 | MIR335 |
| 1229 | ITPR1 |
| 1230 | CEBPD |
| 1231 | MIR186 |
| 1232 | IER3IP1 |
| 1233 | DNA2 |
| 1234 | KCNA2 |
| 1235 | HNRNPA1 |
| 1236 | FOLR1 |
| 1237 | PKD1 |
| 1238 | MECOM |
| 1239 | BCS1L |
| 1240 | KIF1A |
| 1241 | EIF2S1 |
| 1242 | TFRC |
| 1243 | PCAT1 |
| 1244 | PADI4 |
| 1245 | VPS53 |
| 1246 | BAK1 |
| 1247 | P2RY12 |
| 1248 | VKORC1 |
| 1249 | TDP1 |
| 1250 | MIR424 |
| 1251 | TERF1 |
| 1252 | DNMT3B |
| 1253 | CD40 |
| 1254 | FBXW11 |
| 1255 | LTF |
| 1256 | RPS6KC1 |
| 1257 | SPTA1 |
| 1258 | MT-TQ |
| 1259 | RPL36A-HNRNPH2 |
| 1260 | RPL5 |
| 1261 | MAOA |
| 1262 | ATP6AP2 |
| 1263 | LONP1 |
| 1264 | GOT2 |
| 1265 | CLEC7A |
| 1266 | GSS |
| 1267 | CA2 |
| 1268 | MIR199B |
| 1269 | MIR18A |
| 1270 | CNBP |
| 1271 | PCSK9 |
| 1272 | PEX3 |
| 1273 | PARG |
| 1274 | IFT56 |
| 1275 | CREB3L1 |
| 1276 | DNAJC5 |
| 1277 | OTC |
| 1278 | NSD1 |
| 1279 | DAXX |
| 1280 | NR1H3 |
| 1281 | EMX2 |
| 1282 | NRROS |
| 1283 | CCL5 |
| 1284 | IRF3 |
| 1285 | CCL11 |
| 1286 | HK1 |
| 1287 | NDUFS3 |
| 1288 | MIR181C |
| 1289 | KCNQ1OT1 |
| 1290 | TSEN34 |
| 1291 | CYP27B1 |
| 1292 | ARX |
| 1293 | SERPINC1 |
| 1294 | VARS2 |
| 1295 | UBE2D3 |
| 1296 | SYNE1 |
| 1297 | GRB2 |
| 1298 | STX11 |
| 1299 | CCL3 |
| 1300 | VTN |
| 1301 | GATA3 |
| 1302 | APLF |
| 1303 | FHIT |
| 1304 | PPP2CA |
| 1305 | GTPBP2 |
| 1306 | AHCY |
| 1307 | SERPINH1 |
| 1308 | NIPBL |
| 1309 | U2AF1 |
| 1310 | ISCA1 |
| 1311 | MIR19A |
| 1312 | ADH1B |
| 1313 | PYCR1 |
| 1314 | F9 |
| 1315 | FGF10 |
| 1316 | CETN2 |
| 1317 | NFIA |
| 1318 | ADNP |
| 1319 | ALK |
| 1320 | CLPB |
| 1321 | KMT2C |
| 1322 | C1QTNF3-AMACR |
| 1323 | HOTAIRM1 |
| 1324 | PARN |
| 1325 | EGR1 |
| 1326 | AHSG |
| 1327 | EIF2AK1 |
| 1328 | MIR100 |
| 1329 | SLCO1B1 |
| 1330 | MIR182 |
| 1331 | RIPK1 |
| 1332 | BIVM-ERCC5 |
| 1333 | PSMB8 |
| 1334 | RECQL5 |
| 1335 | CDKN3 |
| 1336 | NDUFAF3 |
| 1337 | CACNA1B |
| 1338 | CHAT |
| 1339 | MIR128-2 |
| 1340 | ATP6V1A |
| 1341 | CD34 |
| 1342 | FARSB |
| 1343 | ADH1C |
| 1344 | PIGP |
| 1345 | HCN1 |
| 1346 | ADARB1 |
| 1347 | CD8A |
| 1348 | ITGB3 |
| 1349 | MCM2 |
| 1350 | TTI2 |
| 1351 | TIMP1 |
| 1352 | FAT4 |
| 1353 | SPART |
| 1354 | PRL |
| 1355 | NUMA1 |
| 1356 | TNFSF11 |
| 1357 | OPTN |
| 1358 | CIITA |
| 1359 | FGA |
| 1360 | HNRNPA2B1 |
| 1361 | LYN |
| 1362 | NES |
| 1363 | COG5 |
| 1364 | MIR151A |
| 1365 | NAGA |
| 1366 | CHMP2B |
| 1367 | ACADS |
| 1368 | BMP7 |
| 1369 | TLR9 |
| 1370 | TKT |
| 1371 | F8 |
| 1372 | SMN1 |
| 1373 | DYNC1I2 |
| 1374 | TCAP |
| 1375 | MYRF |
| 1376 | HULC |
| 1377 | AKT2 |
| 1378 | DDX41 |
| 1379 | ATL1 |
| 1380 | BSCL2 |
| 1381 | ATP5F1A |
| 1382 | UHRF1 |
| 1383 | SLC25A13 |
| 1384 | BCL2L11 |
| 1385 | RNASE3 |
| 1386 | IFNB1 |
| 1387 | RAG1 |
| 1388 | GABRG2 |
| 1389 | NKX2-1 |
| 1390 | CKM |
| 1391 | NEK1 |
| 1392 | CD79A |
| 1393 | CEP85L |
| 1394 | CD68 |
| 1395 | LAMA1 |
| 1396 | SLC19A1 |
| 1397 | BOLA3 |
| 1398 | CLTC |
| 1399 | RAD23A |
| 1400 | MIR149 |
| 1401 | RTN4 |
| 1402 | SORL1 |
| 1403 | NFKBIA |
| 1404 | TXNRD2 |
| 1405 | MYO6 |
| 1406 | TBC1D24 |
| 1407 | COA8 |
| 1408 | LMX1B |
| 1409 | ALPL |
| 1410 | RAD21 |
| 1411 | GTF2H2 |
| 1412 | MINPP1 |
| 1413 | DPM2 |
| 1414 | LAMP1 |
| 1415 | AIF1 |
| 1416 | ABCD3 |
| 1417 | CTSA |
| 1418 | FARS2 |
| 1419 | RDH5 |
| 1420 | SLC25A12 |
| 1421 | USP28 |
| 1422 | NCOR2 |
| 1423 | EFTUD2 |
| 1424 | CAV1 |
| 1425 | STXBP2 |
| 1426 | SELENON |
| 1427 | BACE1 |
| 1428 | GEMIN4 |
| 1429 | GLS |
| 1430 | ALOX5 |
| 1431 | APEX2 |
| 1432 | AKAP9 |
| 1433 | CD14 |
| 1434 | RET |
| 1435 | ABCA12 |
| 1436 | CTSK |
| 1437 | EYS |
| 1438 | ATXN3 |
| 1439 | PLCG2 |
| 1440 | SLPI |
| 1441 | PRDX1 |
| 1442 | SMARCA5 |
| 1443 | DONSON |
| 1444 | IQSEC2 |
| 1445 | CACNA1E |
| 1446 | HADHB |
| 1447 | MSH3 |
| 1448 | PSEN2 |
| 1449 | CARD11 |
| 1450 | FLG2 |
| 1451 | TOP2B |
| 1452 | AGER |
| 1453 | STAT5A |
| 1454 | HDAC6 |
| 1455 | SH3TC2 |
| 1456 | CDKN2C |
| 1457 | HDAC3 |
| 1458 | CLCN4 |
| 1459 | UBE3A |
| 1460 | SOX9 |
| 1461 | ANKRD11 |
| 1462 | EME1 |
| 1463 | EMD |
| 1464 | CCNG1 |
| 1465 | CCND3 |
| 1466 | POLD2 |
| 1467 | TRP-AGG2-5 |
| 1468 | TNFSF10 |
| 1469 | IGFBP2 |
| 1470 | UCHL1 |
| 1471 | CCR2 |
| 1472 | ADAMTS13 |
| 1473 | FLT1 |
| 1474 | NDUFS7 |
| 1475 | KCNQ2 |
| 1476 | SST |
| 1477 | MIR107 |
| 1478 | P3H1 |
| 1479 | MIR33A |
| 1480 | NTN1 |
| 1481 | ATP5PO |
| 1482 | NDUFA1 |
| 1483 | LOC129998796 |
| 1484 | UBTF |
| 1485 | SCN3A |
| 1486 | TXN |
| 1487 | MAPK9 |
| 1488 | LOC130056217 |
| 1489 | TJP1 |
| 1490 | ACTL6A |
| 1491 | LRMDA |
| 1492 | CASK |
| 1493 | PNPLA3 |
| 1494 | ACACA |
| 1495 | GDNF |
| 1496 | UGT1A4 |
| 1497 | LGALS3 |
| 1498 | GLRX5 |
| 1499 | YAP1 |
| 1500 | LOC126862571 |
| 1501 | SLC39A14 |
| 1502 | MLLT10 |
| 1503 | CD59 |
| 1504 | F3 |
| 1505 | CYBB |
| 1506 | CNKSR2 |
| 1507 | MIR137 |
| 1508 | IL2RA |
| 1509 | ADRB3 |
| 1510 | STUB1 |
| 1511 | FGF13 |
| 1512 | ATP11A |
| 1513 | CPT1A |
| 1514 | SLC9A6 |
| 1515 | CHKA |
| 1516 | TRAF6 |
| 1517 | IRF1 |
| 1518 | ALDH1L1 |
| 1519 | NRCAM |
| 1520 | D2HGDH |
| 1521 | AP4E1 |
| 1522 | CHRNA4 |
| 1523 | H3-3A |
| 1524 | CNR1 |
| 1525 | HLA-G |
| 1526 | SPTLC1 |
| 1527 | GABBR2 |
| 1528 | COL4A3 |
| 1529 | TJP2 |
| 1530 | TAOK1 |
| 1531 | TOPORS |
| 1532 | CLDN11 |
| 1533 | PTPN23 |
| 1534 | VPS13B |
| 1535 | HECW2 |
| 1536 | CALCA |
| 1537 | ATG5 |
| 1538 | FOXRED1 |
| 1539 | TMEM70 |
| 1540 | TCN2 |
| 1541 | HAPLN1 |
| 1542 | DMPK |
| 1543 | MAPK8IP3 |
| 1544 | CYP2U1 |
| 1545 | ESR2 |
| 1546 | USH2A |
| 1547 | UGT1A9 |
| 1548 | SLC2A2 |
| 1549 | GABRA5 |
| 1550 | IL6R |
| 1551 | TGM1 |
| 1552 | SH2B3 |
| 1553 | HBA1 |
| 1554 | MIR25 |
| 1555 | BUB1B |
| 1556 | PACS2 |
| 1557 | PRMT7 |
| 1558 | BTK |
| 1559 | GH1 |
| 1560 | VPS51 |
| 1561 | C12orf57 |
| 1562 | DNASE1L3 |
| 1563 | H2AC17 |
| 1564 | S100A8 |
| 1565 | GABRB2 |
| 1566 | FGF23 |
| 1567 | METTL3 |
| 1568 | GRIN2D |
| 1569 | ISG15 |
| 1570 | CYP11B2 |
| 1571 | MAP2K2 |
| 1572 | HERC1 |
| 1573 | RORA |
| 1574 | MIR328 |
| 1575 | MFSD8 |
| 1576 | MIR148A |
| 1577 | ADAM17 |
| 1578 | KRT8 |
| 1579 | HPF1 |
| 1580 | MIR183 |
| 1581 | SLC24A5 |
| 1582 | RUVBL1 |
| 1583 | BRSK1 |
| 1584 | ACOT13 |
| 1585 | DCHS1 |
| 1586 | UGT1A10 |
| 1587 | POLL |
| 1588 | FBXO28 |
| 1589 | ALDH3A1 |
| 1590 | NARS1 |
| 1591 | ASCC3 |
| 1592 | EHMT1 |
| 1593 | RXRA |
| 1594 | ACD |
| 1595 | TYROBP |
| 1596 | MVK |
| 1597 | GDF15 |
| 1598 | ERBB4 |
| 1599 | AFG3L2 |
| 1600 | ABCC2 |
| 1601 | APOA2 |
| 1602 | CDC5L |
| 1603 | FKBP10 |
| 1604 | SLC45A2 |
| 1605 | VPS13D |
| 1606 | ATG7 |
| 1607 | H1-4 |
| 1608 | EHHADH |
| 1609 | PNPLA6 |
| 1610 | CHI3L1 |
| 1611 | SREBF2 |
| 1612 | PSMC5 |
| 1613 | CDK10 |
| 1614 | UGT1A8 |
| 1615 | IL3 |
| 1616 | INO80 |
| 1617 | PITX2 |
| 1618 | CYP2C19 |
| 1619 | CCNA2 |
| 1620 | COG7 |
| 1621 | ITGAL |
| 1622 | GAD2 |
| 1623 | IL12RB1 |
| 1624 | LMNB2 |
| 1625 | CTDP1 |
| 1626 | P2RX7 |
| 1627 | H3-4 |
| 1628 | INPP5E |
| 1629 | MT-TW |
| 1630 | ADPRS |
| 1631 | AP4B1-AS1 |
| 1632 | MDH2 |
| 1633 | YARS2 |
| 1634 | NTRK1 |
| 1635 | NT5C2 |
| 1636 | TPI1 |
| 1637 | DCC |
| 1638 | PSMD1 |
| 1639 | ALOX12B |
| 1640 | MIR326 |
| 1641 | MAPK10 |
| 1642 | TRE-TTC3-1 |
| 1643 | BCYRN1 |
| 1644 | DHFR |
| 1645 | GC |
| 1646 | MIR214 |
| 1647 | MIR338 |
| 1648 | GTF2H4 |
| 1649 | NDUFA9 |
| 1650 | PTGS1 |
| 1651 | UBA5 |
| 1652 | COL17A1 |
| 1653 | HBEGF |
| 1654 | ACOT7 |
| 1655 | PHB1 |
| 1656 | JAM3 |
| 1657 | CD274 |
| 1658 | BAZ1B |
| 1659 | MIR200C |
| 1660 | NDUFS2 |
| 1661 | PCLO |
| 1662 | NAMPT |
| 1663 | DGUOK |
| 1664 | HJV |
| 1665 | WDR48 |
| 1666 | TRIM8 |
| 1667 | PPP5C |
| 1668 | LPP |
| 1669 | DLL1 |
| 1670 | BCHE |
| 1671 | AIM2 |
| 1672 | TSPO |
| 1673 | SAG |
| 1674 | NUP214 |
| 1675 | RHEB |
| 1676 | CDC25B |
| 1677 | CYFIP2 |
| 1678 | OSTM1 |
| 1679 | GLI3 |
| 1680 | MIR197 |
| 1681 | TIMELESS |
| 1682 | NDUFC2 |
| 1683 | STIL |
| 1684 | RUVBL2 |
| 1685 | MIR144 |
| 1686 | ELOVL4 |
| 1687 | PICALM |
| 1688 | GPHN |
| 1689 | EIF5 |
| 1690 | PSMA3 |
| 1691 | GATA1 |
| 1692 | DTNBP1 |
| 1693 | GJA3 |
| 1694 | GHRL |
| 1695 | TLR7 |
| 1696 | MICB |
| 1697 | HSPA5 |
| 1698 | GRIN2A |
| 1699 | POLR2F |
| 1700 | KIF1C |
| 1701 | GABRA2 |
| 1702 | PDSS1 |
| 1703 | MIR191 |
| 1704 | IFNGR1 |
| 1705 | TTC5 |
| 1706 | MICA |
| 1707 | COQ8A |
| 1708 | AMPD3 |
| 1709 | DDX5 |
| 1710 | TRNT1 |
| 1711 | LOC126805688 |
| 1712 | MSTO1 |
| 1713 | SUGCT |
| 1714 | OAS1 |
| 1715 | FCGR3A |
| 1716 | FLG |
| 1717 | CEP63 |
| 1718 | SCD |
| 1719 | MIR9-1 |
| 1720 | WNT3 |
| 1721 | FGF8 |
| 1722 | COQ8B |
| 1723 | DCN |
| 1724 | IRS2 |
| 1725 | MIR181A2 |
| 1726 | BCORL1 |
| 1727 | CBX5 |
| 1728 | LPA |
| 1729 | NDP |
| 1730 | RNF213 |
| 1731 | H2AC18 |
| 1732 | NDUFS8 |
| 1733 | DPP4 |
| 1734 | YME1L1 |
| 1735 | KL |
| 1736 | MMP12 |
| 1737 | PWAR1 |
| 1738 | GNB2 |
| 1739 | CELF2 |
| 1740 | GTF2H1 |
| 1741 | G6PC1 |
| 1742 | MATR3 |
| 1743 | NR1H4 |
| 1744 | TGFB2 |
| 1745 | ASXL2 |
| 1746 | NCL |
| 1747 | NDUFAF5 |
| 1748 | PIGL |
| 1749 | TOE1 |
| 1750 | MTRFR |
| 1751 | PYGM |
| 1752 | TAF1 |
| 1753 | GDAP1 |
| 1754 | NUP37 |
| 1755 | ARF1 |
| 1756 | RPGR |
| 1757 | STAG2 |
| 1758 | OFD1 |
| 1759 | INTS3 |
| 1760 | BCOR |
| 1761 | UMOD |
| 1762 | SPTBN1 |
| 1763 | NUDT1 |
| 1764 | SYP |
| 1765 | CDH2 |
| 1766 | CCN2 |
| 1767 | ASAH1 |
| 1768 | SGSH |
| 1769 | PSMC3 |
| 1770 | COX5A |
| 1771 | TGM2 |
| 1772 | QARS1 |
| 1773 | SLC16A2 |
| 1774 | CTNNA2 |
| 1775 | RARS2 |
| 1776 | TOMM40 |
| 1777 | MKS1 |
| 1778 | ABCB6 |
| 1779 | DDX3X |
| 1780 | POLR2B |
| 1781 | RAB27A |
| 1782 | CSNK2B |
| 1783 | PSMA6 |
| 1784 | IGF2R |
| 1785 | CENPF |
| 1786 | HCFC1 |
| 1787 | IARS2 |
| 1788 | PROM1 |
| 1789 | LPAR1 |
| 1790 | AGXT |
| 1791 | H4C1 |
| 1792 | CYP46A1 |
| 1793 | PRKAA2 |
| 1794 | AFG2B |
| 1795 | RRM1 |
| 1796 | RBL2 |
| 1797 | MYO5A |
| 1798 | GALT |
| 1799 | OCRL |
| 1800 | IL12B |
| 1801 | MAOB |
| 1802 | CLCN7 |
| 1803 | PVALB |
| 1804 | BBC3 |
| 1805 | RPE65 |
| 1806 | SOST |
| 1807 | IFI27 |
| 1808 | SUMO1 |
| 1809 | FAN1 |
| 1810 | HMGA1 |
| 1811 | DHDDS |
| 1812 | CCNH |
| 1813 | ACTN4 |
| 1814 | B3GALNT2 |
| 1815 | PHC1 |
| 1816 | ITGA4 |
| 1817 | CACNA2D1 |
| 1818 | SI |
| 1819 | MIR152 |
| 1820 | SRA1 |
| 1821 | STX1A |
| 1822 | MIR330 |
| 1823 | ABAT |
| 1824 | MIR324 |
| 1825 | PSAT1 |
| 1826 | PFAS |
| 1827 | RPL10 |
| 1828 | IREB2 |
| 1829 | UBE2L3 |
| 1830 | LOC126862264 |
| 1831 | LCN2 |
| 1832 | CYP2A6 |
| 1833 | CD19 |
| 1834 | CARD9 |
| 1835 | POMK |
| 1836 | DPAGT1 |
| 1837 | WNT7A |
| 1838 | ABCD4 |
| 1839 | CDK9 |
| 1840 | PIN1 |
| 1841 | STAT4 |
| 1842 | SGK1 |
| 1843 | FOXC2 |
| 1844 | HSPA1A |
| 1845 | CCDC88C |
| 1846 | BCL11B |
| 1847 | PTH |
| 1848 | KCNT1 |
| 1849 | SUCLA2 |
| 1850 | BANF1 |
| 1851 | MACROH2A1 |
| 1852 | POLRMT |
| 1853 | FTH1 |
| 1854 | MIRLET7I |
| 1855 | MIR130B |
| 1856 | COG2 |
| 1857 | NUBPL |
| 1858 | NHP2 |
| 1859 | TACO1 |
| 1860 | ALG12 |
| 1861 | AP3B2 |
| 1862 | NCAPD3 |
| 1863 | GHR |
| 1864 | ATP8B1 |
| 1865 | NOTCH2NLA |
| 1866 | ACHE |
| 1867 | MIR125B1 |
| 1868 | TEN1 |
| 1869 | SNAP25 |
| 1870 | AKR1B1 |
| 1871 | PIGQ |
| 1872 | NDRG1 |
| 1873 | BUB3 |
| 1874 | PDHX |
| 1875 | SMARCE1 |
| 1876 | NPHS2 |
| 1877 | SKIC2 |
| 1878 | REEP1 |
| 1879 | PSMB4 |
| 1880 | NDUFB11 |
| 1881 | SMO |
| 1882 | SLC35A2 |
| 1883 | MIR96 |
| 1884 | PGAP1 |
| 1885 | TET3 |
| 1886 | H2AC4 |
| 1887 | TMEM126B |
| 1888 | KLK3 |
| 1889 | LRRC37A2 |
| 1890 | IFNAR2 |
| 1891 | UGT1A3 |
| 1892 | IGHMBP2 |
| 1893 | GAMT |
| 1894 | ATP1A1 |
| 1895 | HIC1 |
| 1896 | SERPINA3 |
| 1897 | MAP3K1 |
| 1898 | HSD17B10 |
| 1899 | SPIRE2 |
| 1900 | TUBA8 |
| 1901 | HBS1L |
| 1902 | TCF3 |
| 1903 | BAD |
| 1904 | NPHP1 |
| 1905 | CCDC134 |
| 1906 | SPINK1 |
| 1907 | SYNJ1 |
| 1908 | COL18A1 |
| 1909 | CFLAR |
| 1910 | CLCN1 |
| 1911 | YWHAQ |
| 1912 | OLIG1 |
| 1913 | KRT16 |
| 1914 | CD63 |
| 1915 | DHX16 |
| 1916 | RPL11 |
| 1917 | ZBTB20 |
| 1918 | RALGAPA1 |
| 1919 | NDUFAF1 |
| 1920 | CDK5RAP2 |
| 1921 | PECAM1 |
| 1922 | CRBN |
| 1923 | ADD3 |
| 1924 | HAMP |
| 1925 | ZNF276 |
| 1926 | FASN |
| 1927 | LEMD2 |
| 1928 | IGHE |
| 1929 | IL18R1 |
| 1930 | SIRT2 |
| 1931 | GNAO1 |
| 1932 | CRB1 |
| 1933 | GLDC |
| 1934 | SSR4 |
| 1935 | MIR154 |
| 1936 | COL11A1 |
| 1937 | WASHC5 |
| 1938 | HPS4 |
| 1939 | ARID1B |
| 1940 | RNASEL |
| 1941 | PAX5 |
| 1942 | RBFOX1 |
| 1943 | LCK |
| 1944 | MAF |
| 1945 | NR1H2 |
| 1946 | MYB |
| 1947 | S100A9 |
| 1948 | MOGS |
| 1949 | CTBP2 |
| 1950 | GPR17 |
| 1951 | DHX15 |
| 1952 | FBXO31 |
| 1953 | TRPV1 |
| 1954 | PSMD4 |
| 1955 | USB1 |
| 1956 | PON2 |
| 1957 | PDE6B |
| 1958 | CFHR5 |
| 1959 | FZD4 |
| 1960 | MIR211 |
| 1961 | MSH5 |
| 1962 | ANGPTL3 |
| 1963 | SLC13A3 |
| 1964 | SLC9A3 |
| 1965 | NKX2-5 |
| 1966 | DTX3L |
| 1967 | HSPA9 |
| 1968 | KRT17 |
| 1969 | CFTR-AS1 |
| 1970 | MIR106A |
| 1971 | AMBP |
| 1972 | PLAUR |
| 1973 | HOTTIP |
| 1974 | HIP1 |
| 1975 | MIR29B1 |
| 1976 | IL15 |
| 1977 | BCL10 |
| 1978 | LOC102724058 |
| 1979 | LOC130059394 |
| 1980 | ALDH9A1 |
| 1981 | MIR26A1 |
| 1982 | BAG6 |
| 1983 | NEDD4L |
| 1984 | NUS1 |
| 1985 | CDKN1C |
| 1986 | CDK19 |
| 1987 | CCAT1 |
| 1988 | MOCS1 |
| 1989 | HROB |
| 1990 | PKD2 |
| 1991 | MTREX |
| 1992 | HPS1 |
| 1993 | MAP2 |
| 1994 | TAFAZZIN |
| 1995 | LOC129992330 |
| 1996 | TMEM38B |
| 1997 | H4C16 |
| 1998 | NONO |
| 1999 | F13A1 |
| 2000 | INF2 |
| 2001 | LAMA5 |
| 2002 | LIPE |
| 2003 | BLTP1 |
| 2004 | ATXN7 |
| 2005 | NPC2 |
| 2006 | KCNA1 |
| 2007 | LAMB2 |
| 2008 | GUCY2D |
| 2009 | ALX4 |
| 2010 | MICU1 |
| 2011 | CUL5 |
| 2012 | LARS2 |
| 2013 | AOC3 |
| 2014 | CERKL |
| 2015 | KRT1 |
| 2016 | ERLIN2 |
| 2017 | SCAF4 |
| 2018 | ZFR |
| 2019 | LOC130002059 |
| 2020 | WIPF1 |
| 2021 | CCK |
| 2022 | KRT19 |
| 2023 | KIDINS220 |
| 2024 | POLDIP2 |
| 2025 | ITPA |
| 2026 | PNP |
| 2027 | CTSB |
| 2028 | MIR98 |
| 2029 | MIRLET7G |
| 2030 | SLC25A22 |
| 2031 | COPS5 |
| 2032 | DNAJC21 |
| 2033 | HNRNPH1 |
| 2034 | PRSS1 |
| 2035 | PLOD2 |
| 2036 | MIR486-1 |
| 2037 | TNFRSF11A |
| 2038 | HSF1 |
| 2039 | EXOSC9 |
| 2040 | SFTPC |
| 2041 | SLC6A8 |
| 2042 | BMP1 |
| 2043 | CKMT2 |
| 2044 | IFI16 |
| 2045 | CPSF3 |
| 2046 | NGLY1 |
| 2047 | BOD1L1 |
| 2048 | PIK3CB |
| 2049 | HMBS |
| 2050 | SYNGR2 |
| 2051 | GCLC |
| 2052 | FNDC5 |
| 2053 | IL22 |
| 2054 | RAI1 |
| 2055 | MTM1 |
| 2056 | AP3D1 |
| 2057 | EZR |
| 2058 | AFG2A |
| 2059 | P4HB |
| 2060 | NPHP4 |
| 2061 | H2AC14 |
| 2062 | ADM |
| 2063 | USP8 |
| 2064 | CBFB |
| 2065 | SCP2 |
| 2066 | PTPN1 |
| 2067 | GAN |
| 2068 | MPDZ |
| 2069 | NCOA1 |
| 2070 | SHBG |
| 2071 | LOC107303340 |
| 2072 | IL6ST |
| 2073 | TARS2 |
| 2074 | IQCB1 |
| 2075 | DACT1 |
| 2076 | STRADA |
| 2077 | DKK1 |
| 2078 | UGT1A5 |
| 2079 | G3BP1 |
| 2080 | GCG |
| 2081 | VLDLR |
| 2082 | CKMT1B |
| 2083 | NRXN1 |
| 2084 | TGIF1 |
| 2085 | MT-TV |
| 2086 | ATF3 |
| 2087 | PAX2 |
| 2088 | ROBO1 |
| 2089 | MGP |
| 2090 | UGT1A |
| 2091 | DLEU1 |
| 2092 | RAB3GAP1 |
| 2093 | LAMC1 |
| 2094 | NMNAT1 |
| 2095 | PRDX5 |
| 2096 | PUS3 |
| 2097 | H4C6 |
| 2098 | SLC34A1 |
| 2099 | PLAU |
| 2100 | COL6A3 |
| 2101 | CIC |
| 2102 | HPD |
| 2103 | FLNB |
| 2104 | H3-3B |
| 2105 | ANO5 |
| 2106 | COQ2 |
| 2107 | CHIT1 |
| 2108 | FUCA1 |
| 2109 | AHDC1 |
| 2110 | AURKB |
| 2111 | PDHB |
| 2112 | GNB3 |
| 2113 | CNTNAP1 |
| 2114 | RCBTB1 |
| 2115 | EYA1 |
| 2116 | H4C3 |
| 2117 | TIMMDC1 |
| 2118 | CHGA |
| 2119 | MIR192 |
| 2120 | GNRH1 |
| 2121 | CEP152 |
| 2122 | RNASEH1 |
| 2123 | PPIG |
| 2124 | SEC23B |
| 2125 | KCNN2 |
| 2126 | H2AC13 |
| 2127 | H2AC16 |
| 2128 | TRC-GCA24-1 |
| 2129 | KDM4B |
| 2130 | MIR331 |
| 2131 | GNA11 |
| 2132 | SIN3A |
| 2133 | TNFSF13B |
| 2134 | DOCK8 |
| 2135 | PIKFYVE |
| 2136 | EXOSC3 |
| 2137 | RRM2 |
| 2138 | HAVCR2 |
| 2139 | FECH |
| 2140 | DVL1 |
| 2141 | RTN4IP1 |
| 2142 | DHCR7 |
| 2143 | DDX39B |
| 2144 | ZSWIM6 |
| 2145 | RIGI |
| 2146 | PROS1 |
| 2147 | TMEM107 |
| 2148 | NFU1 |
| 2149 | MED12 |
| 2150 | PIK3C3 |
| 2151 | UBE2D2 |
| 2152 | CR1 |
| 2153 | YBX1 |
| 2154 | NOX4 |
| 2155 | GNPTAB |
| 2156 | NFKBIL1 |
| 2157 | HPS5 |
| 2158 | CENPE |
| 2159 | UGT8 |
| 2160 | FBLN5 |
| 2161 | SMARCC2 |
| 2162 | NR1I2 |
| 2163 | MAN2B1 |
| 2164 | COMP |
| 2165 | HLA-DRB5 |
| 2166 | GSTM3 |
| 2167 | NCOR1 |
| 2168 | LRP12 |
| 2169 | SIRT3 |
| 2170 | PCYT2 |
| 2171 | TONSL |
| 2172 | RCC2 |
| 2173 | SOX2 |
| 2174 | MPZL2 |
| 2175 | HNRNPR |
| 2176 | CRX |
| 2177 | LRPPRC |
| 2178 | SYNE2 |
| 2179 | LITAF |
| 2180 | UFM1 |
| 2181 | ACADVL |
| 2182 | DSG1 |
| 2183 | NCAM1 |
| 2184 | HBG2 |
| 2185 | MMP7 |
| 2186 | ETS1 |
| 2187 | SLX1A-SULT1A3 |
| 2188 | HARS2 |
| 2189 | C5 |
| 2190 | GPX3 |
| 2191 | PUF60 |
| 2192 | PMS1 |
| 2193 | MIR134 |
| 2194 | H3C12 |
| 2195 | NDUFAF8 |
| 2196 | COQ4 |
| 2197 | TSFM |
| 2198 | MIR378A |
| 2199 | RABIF |
| 2200 | CXCL2 |
| 2201 | ANGPTL4 |
| 2202 | BRD2 |
| 2203 | TBX18 |
| 2204 | HMGA2 |
| 2205 | APOC1 |
| 2206 | MIR296 |
| 2207 | TBCE |
| 2208 | DHX36 |
| 2209 | MIR185 |
| 2210 | GPT2 |
| 2211 | IL12A |
| 2212 | GFER |
| 2213 | FKBP5 |
| 2214 | ARHGAP31 |
| 2215 | ASXL3 |
| 2216 | CDON |
| 2217 | ABCD2 |
| 2218 | ANK3 |
| 2219 | TRIM65 |
| 2220 | ACOT11 |
| 2221 | RPL26 |
| 2222 | GPSM2 |
| 2223 | CCNF |
| 2224 | SERPINB8 |
| 2225 | FGF12 |
| 2226 | TRMT10A |
| 2227 | CHKB |
| 2228 | YWHAZ |
| 2229 | ETFA |
| 2230 | CELF1 |
| 2231 | ITPR3 |
| 2232 | MTTP |
| 2233 | EGR2 |
| 2234 | MCM9 |
| 2235 | PDP1 |
| 2236 | GRIA2 |
| 2237 | CTNS-AS1 |
| 2238 | PRKCH |
| 2239 | COL6A1 |
| 2240 | NECAP1 |
| 2241 | ATP7A |
| 2242 | ZMPSTE24 |
| 2243 | E2F7 |
| 2244 | MIR206 |
| 2245 | HPS6 |
| 2246 | SLC2A3 |
| 2247 | PPP1CA |
| 2248 | RASSF1 |
| 2249 | BLOC1S6 |
| 2250 | NTF3 |
| 2251 | SBF2 |
| 2252 | MLKL |
| 2253 | PGK1 |
| 2254 | LINC02210 |
| 2255 | ASNS |
| 2256 | ARV1 |
| 2257 | MGAM |
| 2258 | CYP17A1 |
| 2259 | SLC25A19 |
| 2260 | NEUROD2 |
| 2261 | CLASRP |
| 2262 | SUPT5H |
| 2263 | IGF2-AS |
| 2264 | TNKS |
| 2265 | NFKB2 |
| 2266 | PRORP-PSMA6 |
| 2267 | IL2RB |
| 2268 | SH3PXD2A |
| 2269 | PCCB |
| 2270 | PTPN3 |
| 2271 | HSPA12A |
| 2272 | OLR1 |
| 2273 | TSPAN12 |
| 2274 | PNPLA1 |
| 2275 | LINC01554 |
| 2276 | FLCN |
| 2277 | TRRAP |
| 2278 | SCN1B |
| 2279 | IFNA2 |
| 2280 | CDKN2AIP |
| 2281 | PCNT |
| 2282 | CHD3 |
| 2283 | SUPT3H |
| 2284 | STAT6 |
| 2285 | ATXN1 |
| 2286 | SATB2 |
| 2287 | MAL |
| 2288 | FGF21 |
| 2289 | MC4R |
| 2290 | IL7 |
| 2291 | FARSA |
| 2292 | CPLX1 |
| 2293 | HCRT |
| 2294 | ANGPT1 |
| 2295 | TYK2 |
| 2296 | SLC39A8 |
| 2297 | IRF8 |
| 2298 | NAXD |
| 2299 | FAAH |
| 2300 | TECPR2 |
| 2301 | B4GAT1 |
| 2302 | H2BC15 |
| 2303 | CDK13 |
| 2304 | ALG3 |
| 2305 | SFTPB |
| 2306 | LDB3 |
| 2307 | GNA13 |
| 2308 | LRP1 |
| 2309 | ALG11 |
| 2310 | POU5F1 |
| 2311 | PLEKHM1 |
| 2312 | BGLT3 |
| 2313 | MIR532 |
| 2314 | GAS1 |
| 2315 | RAB3GAP2 |
| 2316 | CRH |
| 2317 | SOX11 |
| 2318 | CNTF |
| 2319 | CALB1 |
| 2320 | TSHR |
| 2321 | CD82 |
| 2322 | ALOXE3 |
| 2323 | APOL1 |
| 2324 | ARHGEF6 |
| 2325 | ALPP |
| 2326 | LOC126860971 |
| 2327 | LIPT1 |
| 2328 | SDHD |
| 2329 | CYP11A1 |
| 2330 | GNAQ |
| 2331 | FKBP14 |
| 2332 | ZFAS1 |
| 2333 | NFATC1 |
| 2334 | IFITM5 |
| 2335 | DPYSL5 |
| 2336 | TENT5A |
| 2337 | IFNAR1 |
| 2338 | MED25 |
| 2339 | MMP13 |
| 2340 | BECN1 |
| 2341 | PTPA |
| 2342 | VANGL2 |
| 2343 | DPH1 |
| 2344 | ABCC6 |
| 2345 | B4GALNT1 |
| 2346 | TGFA |
| 2347 | ZIC2 |
| 2348 | HK3 |
| 2349 | TFAP2A |
| 2350 | CXCR2 |
| 2351 | CYP21A2 |
| 2352 | COPS8 |
| 2353 | TIMP2 |
| 2354 | PKHD1 |
| 2355 | KRTAP9-9 |
| 2356 | C16orf95 |
| 2357 | FDFT1 |
| 2358 | SLC12A5 |
| 2359 | MAP3K7 |
| 2360 | H4C5 |
| 2361 | ACP1 |
| 2362 | HMCES |
| 2363 | RFC2 |
| 2364 | CLOCK |
| 2365 | PRKCB |
| 2366 | PEX7 |
| 2367 | DPP6 |
| 2368 | RS1 |
| 2369 | PRPF8 |
| 2370 | OPRM1 |
| 2371 | PYY |
| 2372 | SALL1 |
| 2373 | DIAPH1 |
| 2374 | MX1 |
| 2375 | GTF2H3 |
| 2376 | YWHAB |
| 2377 | P2RX5-TAX1BP3 |
| 2378 | CXCR3 |
| 2379 | LOC126860970 |
| 2380 | COPA |
| 2381 | NEFH |
| 2382 | LOX |
| 2383 | MARS1 |
| 2384 | PCTP |
| 2385 | RARB |
| 2386 | MARS2 |
| 2387 | TBX5 |
| 2388 | EPHB4 |
| 2389 | NDUFV2 |
| 2390 | NSMCE2 |
| 2391 | CYSLTR1 |
| 2392 | AP2M1 |
| 2393 | CAD |
| 2394 | ECHS1 |
| 2395 | COASY |
| 2396 | ACAN |
| 2397 | MSR1 |
| 2398 | CYP4F22 |
| 2399 | KDR |
| 2400 | PCCA |
| 2401 | TMEM63C |
| 2402 | ERF |
| 2403 | SUZ12 |
| 2404 | UBQLN2 |
| 2405 | EIF1 |
| 2406 | THOC1 |
| 2407 | IKZF3 |
| 2408 | CYLD |
| 2409 | HSPB8 |
| 2410 | RPGRIP1 |
| 2411 | GPX5 |
| 2412 | NDUFAF6 |
| 2413 | EPG5 |
| 2414 | MYOD1 |
| 2415 | KIF5C |
| 2416 | COX15 |
| 2417 | NCAPH2 |
| 2418 | WNT9B |
| 2419 | ALG2 |
| 2420 | SNRPN |
| 2421 | SLC25A46 |
| 2422 | KMT2E |
| 2423 | UPF1 |
| 2424 | MFRP |
| 2425 | PDX1 |
| 2426 | TMEM119 |
| 2427 | SLC5A6 |
| 2428 | SMCHD1 |
| 2429 | PPP4R3A |
| 2430 | SARS1 |
| 2431 | TERF2IP |
| 2432 | IL33 |
| 2433 | BANK1 |
| 2434 | SMC2 |
| 2435 | MT-TI |
| 2436 | CEP135 |
| 2437 | H4C11 |
| 2438 | CXCL9 |
| 2439 | TRPM3 |
| 2440 | APBB1 |
| 2441 | ARMC9 |
| 2442 | CDK8 |
| 2443 | DDX1 |
| 2444 | HSPB2 |
| 2445 | CDC27 |
| 2446 | ADAMTS4 |
| 2447 | CSNK2A2 |
| 2448 | LOC111674472 |
| 2449 | SUFU |
| 2450 | IK |
| 2451 | SLC1A4 |
| 2452 | AIRE |
| 2453 | NOP10 |
| 2454 | DST |
| 2455 | MIR212 |
| 2456 | REL |
| 2457 | SGPL1 |
| 2458 | WDR73 |
| 2459 | AGPAT2 |
| 2460 | IL11 |
| 2461 | GGT2P |
| 2462 | OTX2 |
| 2463 | GAL3ST1 |
| 2464 | VPS4A |
| 2465 | CLCN5 |
| 2466 | PGM1 |
| 2467 | GRM5 |
| 2468 | NOP56 |
| 2469 | PPP1R12A |
| 2470 | KIAA0586 |
| 2471 | DNTT |
| 2472 | PTGDS |
| 2473 | OPA3 |
| 2474 | IFNG-AS1 |
| 2475 | GOSR2 |
| 2476 | PDE4D |
| 2477 | COG4 |
| 2478 | H4C15 |
| 2479 | CRLF1 |
| 2480 | ASL |
| 2481 | POMGNT2 |
| 2482 | MRPS22 |
| 2483 | WDR12 |
| 2484 | SH2D1A |
| 2485 | CFB |
| 2486 | DPM1 |
| 2487 | EGFR-AS1 |
| 2488 | MYO15A |
| 2489 | NUAK1 |
| 2490 | APOBEC3G |
| 2491 | ELP1 |
| 2492 | SHC1 |
| 2493 | PLPBP |
| 2494 | CRB2 |
| 2495 | SLC22A5 |
| 2496 | TRAK1 |
| 2497 | UBE2D1 |
| 2498 | PWAR4 |
| 2499 | TGFBR1 |
| 2500 | KCNJ2 |
| 2501 | NIPAL4 |
| 2502 | KNG1 |
| 2503 | KDM5C |
| 2504 | IRF7 |
| 2505 | SCN4A |
| 2506 | SHMT2 |
| 2507 | MIR215 |
| 2508 | ADCY5 |
| 2509 | PLXNB3-AS1 |
| 2510 | MIR503 |
| 2511 | SERPINB1 |
| 2512 | FMN2 |
| 2513 | NCOA3 |
| 2514 | SLC44A1 |
| 2515 | DOCK6 |
| 2516 | POLR2L |
| 2517 | PTK2 |
| 2518 | KIFBP |
| 2519 | TRPS1 |
| 2520 | DNAAF4 |
| 2521 | PPP2R2B |
| 2522 | FLVCR1 |
| 2523 | CYP4V2 |
| 2524 | HPS3 |
| 2525 | SFPQ |
| 2526 | COQ6 |
| 2527 | AHI1 |
| 2528 | PRDM5 |
| 2529 | TULP1 |
| 2530 | SLC25A15 |
| 2531 | CCR7 |
| 2532 | TOR1A |
| 2533 | TLX1NB |
| 2534 | CHD8 |
| 2535 | TAOK3 |
| 2536 | C5AR1 |
| 2537 | OCLN |
| 2538 | LIFR |
| 2539 | ABCA7 |
| 2540 | CDC14A |
| 2541 | PF4 |
| 2542 | EFL1 |
| 2543 | PLA2G5 |
| 2544 | H2AC25 |
| 2545 | NDUFAF4 |
| 2546 | ZIC1 |
| 2547 | DDC |
| 2548 | APRT |
| 2549 | H4C14 |
| 2550 | GRM7 |
| 2551 | NCF1 |
| 2552 | KMT5B |
| 2553 | VDAC1 |
| 2554 | USP9X |
| 2555 | DLG4 |
| 2556 | RTTN |
| 2557 | CYP2B6 |
| 2558 | MIR155HG |
| 2559 | TPT1 |
| 2560 | FLI1 |
| 2561 | DLL4 |
| 2562 | CACNB2 |
| 2563 | H4C9 |
| 2564 | MIR339 |
| 2565 | TFEB |
| 2566 | TGFBI |
| 2567 | DNM2 |
| 2568 | TMTC3 |
| 2569 | SYNGAP1 |
| 2570 | KRT2 |
| 2571 | NR3C2 |
| 2572 | RAB7A |
| 2573 | NEBL |
| 2574 | TFR2 |
| 2575 | MIR181B1 |
| 2576 | CLMP |
| 2577 | HAX1 |
| 2578 | CD163 |
| 2579 | GNS |
| 2580 | PTPN6 |
| 2581 | H4C8 |
| 2582 | RPN1 |
| 2583 | GZMB |
| 2584 | GNE |
| 2585 | FLII |
| 2586 | KLF1 |
| 2587 | DALRD3 |
| 2588 | TRAIP |
| 2589 | EPHA2 |
| 2590 | LCAT |
| 2591 | IL36RN |
| 2592 | ITGAV |
| 2593 | PRKCZ |
| 2594 | HLCS |
| 2595 | HGSNAT |
| 2596 | MSH4 |
| 2597 | SMCR8 |
| 2598 | RBMX |
| 2599 | MT-ND4L |
| 2600 | TBXAS1 |
| 2601 | SFTPD |
| 2602 | DDX6 |
| 2603 | H2AC6 |
| 2604 | CRADD |
| 2605 | BMPR2 |
| 2606 | SCN9A |
| 2607 | PEPD |
| 2608 | HSP90B1 |
| 2609 | SETMAR |
| 2610 | CDC73 |
| 2611 | MMP14 |
| 2612 | C2 |
| 2613 | CCDC88A |
| 2614 | SESN2 |
| 2615 | FAH |
| 2616 | CAPN1 |
| 2617 | TNFRSF10A |
| 2618 | LTBP1 |
| 2619 | AKR1A1 |
| 2620 | CNOT1 |
| 2621 | GPR143 |
| 2622 | TCIRG1 |
| 2623 | PSMC2 |
| 2624 | CDK12 |
| 2625 | LDHA |
| 2626 | PCDH15 |
| 2627 | IMMT |
| 2628 | NDUFA8 |
| 2629 | UGCG |
| 2630 | SRGAP2 |
| 2631 | ITGB4 |
| 2632 | TIA1 |
| 2633 | FASTKD2 |
| 2634 | H2BC13 |
| 2635 | PORCN |
| 2636 | COX6B1 |
| 2637 | CD80 |
| 2638 | PIGB |
| 2639 | SNHG1 |
| 2640 | KDM6A |
| 2641 | DBT |
| 2642 | H4C4 |
| 2643 | H4C12 |
| 2644 | H4C13 |
| 2645 | PSMA1 |
| 2646 | ALG8 |
| 2647 | ZNF408 |
| 2648 | KCNJ5 |
| 2649 | RPGRIP1L |
| 2650 | OAT |
| 2651 | CABIN1 |
| 2652 | KCNMA1 |
| 2653 | ATF6 |
| 2654 | MMUT |
| 2655 | MED1 |
| 2656 | NACC1 |
| 2657 | MIR196A1 |
| 2658 | KCNN4 |
| 2659 | TSLP |
| 2660 | LOC130056971 |
| 2661 | KCNE2 |
| 2662 | UFSP2 |
| 2663 | PLA2G2A |
| 2664 | MVP |
| 2665 | LETM1 |
| 2666 | OTUD5 |
| 2667 | MIR361 |
| 2668 | MAP3K14 |
| 2669 | RBBP4 |
| 2670 | DIABLO |
| 2671 | CTR9 |
| 2672 | PGM3 |
| 2673 | FOSL1 |
| 2674 | UFC1 |
| 2675 | OTUD6B |
| 2676 | SLC38A3 |
| 2677 | NSD3 |
| 2678 | MIR491 |
| 2679 | SUCLG1 |
| 2680 | TG |
| 2681 | H2AC19 |
| 2682 | CASP7 |
| 2683 | NR2F2 |
| 2684 | ANPEP |
| 2685 | WNT3A |
| 2686 | NDUFS6 |
| 2687 | SCARB1 |
| 2688 | DRD1 |
| 2689 | ELOA |
| 2690 | PANK2 |
| 2691 | TNXB |
| 2692 | PLCD1 |
| 2693 | TMEM147 |
| 2694 | LEF1 |
| 2695 | PRRX1 |
| 2696 | BLZF1 |
| 2697 | HADH |
| 2698 | FOXP2 |
| 2699 | ERC1 |
| 2700 | INTS7 |
| 2701 | MUSK |
| 2702 | CCL4 |
| 2703 | PTHLH |
| 2704 | LOC100287944 |
| 2705 | HCCAT5 |
| 2706 | MAN2C1 |
| 2707 | MORF4L1 |
| 2708 | MLPH |
| 2709 | B3GLCT |
| 2710 | STK4 |
| 2711 | FHL1 |
| 2712 | DSPP |
| 2713 | EHMT2 |
| 2714 | IVD |
| 2715 | COPB2 |
| 2716 | SNIP1 |
| 2717 | HSP90AB1 |
| 2718 | DHX30 |
| 2719 | TMEM165 |
| 2720 | PSMA4 |
| 2721 | PSME3 |
| 2722 | MALT1 |
| 2723 | RPS19 |
| 2724 | ANTXR2 |
| 2725 | KCNQ4 |
| 2726 | ARID2 |
| 2727 | HNRNPH2 |
| 2728 | PSMD7 |
| 2729 | POR |
| 2730 | RDH12 |
| 2731 | SCT |
| 2732 | S1PR1 |
| 2733 | FLVCR2 |
| 2734 | H4C2 |
| 2735 | KHDRBS1 |
| 2736 | BRPF1 |
| 2737 | SLC27A4 |
| 2738 | SPTLC3 |
| 2739 | SELENOP |
| 2740 | SIN3B |
| 2741 | IVL |
| 2742 | CD244 |
| 2743 | C1QBP |
| 2744 | DMAP1 |
| 2745 | TNC |
| 2746 | ANKLE1 |
| 2747 | KIF21A |
| 2748 | NUP85 |
| 2749 | ENO1 |
| 2750 | ABCF1 |
| 2751 | MRI1 |
| 2752 | ETHE1 |
| 2753 | MIR139 |
| 2754 | SLC18A3 |
| 2755 | COG6 |
| 2756 | PPARGC1B |
| 2757 | SOX2-OT |
| 2758 | SMARCC1 |
| 2759 | TRMT1 |
| 2760 | CTSL |
| 2761 | SKP1 |
| 2762 | UNC80 |
| 2763 | SPTB |
| 2764 | AXIN1 |
| 2765 | DSTYK |
| 2766 | ALDH7A1 |
| 2767 | PGR |
| 2768 | ALAS2 |
| 2769 | CD27 |
| 2770 | MFF-DT |
| 2771 | OXTR |
| 2772 | AGL |
| 2773 | KDELR2 |
| 2774 | MOV10 |
| 2775 | XAB2 |
| 2776 | ANXA1 |
| 2777 | ABCA2 |
| 2778 | PHEX |
| 2779 | NUP133 |
| 2780 | F7 |
| 2781 | LURAP1L-AS1 |
| 2782 | CRYGD |
| 2783 | ADGRV1 |
| 2784 | KATNIP |
| 2785 | RFC5 |
| 2786 | CWF19L1 |
| 2787 | TMC1 |
| 2788 | SUMO2 |
| 2789 | NMT1 |
| 2790 | NIPA1 |
| 2791 | TRAPPC4 |
| 2792 | KIF11 |
| 2793 | ALAD |
| 2794 | SAA1 |
| 2795 | RFC3 |
| 2796 | TRMU |
| 2797 | PIGG |
| 2798 | CANX |
| 2799 | MAT1A |
| 2800 | PRDX2 |
| 2801 | ALDH1A1 |
| 2802 | SOX4 |
| 2803 | IGFBP1 |
| 2804 | PLK4 |
| 2805 | PHLDB1 |
| 2806 | TBCD |
| 2807 | PRICKLE2 |
| 2808 | PCBD1 |
| 2809 | PIGN |
| 2810 | TPO |
| 2811 | GRIA4 |
| 2812 | PFN1 |
| 2813 | YWHAH |
| 2814 | DPYS |
| 2815 | MCTS1 |
| 2816 | KRT10 |
| 2817 | NDUFA10 |
| 2818 | LOC129930561 |
| 2819 | WAC |
| 2820 | SPEN |
| 2821 | SNAP23 |
| 2822 | SMAD7 |
| 2823 | RXRB |
| 2824 | ALG13 |
| 2825 | GP1BB |
| 2826 | EEF2 |
| 2827 | LOC130056973 |
| 2828 | NAA10 |
| 2829 | PDSS2 |
| 2830 | RNY5 |
| 2831 | THOC2 |
| 2832 | BLOC1S5 |
| 2833 | COQ9 |
| 2834 | ARSH |
| 2835 | LYVE1 |
| 2836 | KIF14 |
| 2837 | H1-0 |
| 2838 | RHOB |
| 2839 | DCAF1 |
| 2840 | SOS1 |
| 2841 | HAVCR1 |
| 2842 | SRSF1 |
| 2843 | NT5E |
| 2844 | MT-TG |
| 2845 | THBS1 |
| 2846 | SCGB1A1 |
| 2847 | GTF2I |
| 2848 | C1QA |
| 2849 | LGI1 |
| 2850 | SULT1A3 |
| 2851 | TMEM231 |
| 2852 | HECTD4 |
| 2853 | EOMES |
| 2854 | RPS20 |
| 2855 | RASA1 |
| 2856 | ADAMTS9 |
| 2857 | C1QTNF5 |
| 2858 | FGF7 |
| 2859 | MCM3 |
| 2860 | NEPRO |
| 2861 | EXOC2 |
| 2862 | CASP6 |
| 2863 | PRKCA |
| 2864 | CTNNA3 |
| 2865 | PKM |
| 2866 | CAMK2D |
| 2867 | ITGA2B |
| 2868 | CCT5 |
| 2869 | ABCC5 |
| 2870 | DLL3 |
| 2871 | NELFA |
| 2872 | TRIM21 |
| 2873 | SRP54 |
| 2874 | TUBGCP2 |
| 2875 | PBX1 |
| 2876 | COPS6 |
| 2877 | MTHFS |
| 2878 | ACSF3 |
| 2879 | CRHR1 |
| 2880 | NDUFB9 |
| 2881 | OGT |
| 2882 | COX4I1 |
| 2883 | PRSS2 |
| 2884 | SPTBN4 |
| 2885 | SLC26A9 |
| 2886 | PRRC2A |
| 2887 | CSNK1A1 |
| 2888 | MIR224 |
| 2889 | GAB1 |
| 2890 | HCP5 |
| 2891 | IL1RAPL2 |
| 2892 | EWSR1 |
| 2893 | TLR8 |
| 2894 | MUC5AC |
| 2895 | NSUN3 |
| 2896 | CEACAM3 |
| 2897 | SYCE2 |
| 2898 | WNT5A |
| 2899 | NANS |
| 2900 | IMPDH2 |
| 2901 | IL9 |
| 2902 | NDUFB3 |
| 2903 | PIEZO2 |
| 2904 | TUBB4B |
| 2905 | BCKDHA |
| 2906 | CSPG4 |
| 2907 | CIT |
| 2908 | TMEM98 |
| 2909 | CAV3 |
| 2910 | ACAD9 |
| 2911 | ACBD5 |
| 2912 | DICER1 |
| 2913 | C19orf12 |
| 2914 | NUP107 |
| 2915 | RANBP9 |
| 2916 | APOD |
| 2917 | MIR340 |
| 2918 | THOC6 |
| 2919 | LOC126862363 |
| 2920 | SLC52A2 |
| 2921 | VIP |
| 2922 | ATXN8OS |
| 2923 | TGM6 |
| 2924 | AVP |
| 2925 | HNRNPC |
| 2926 | ADRA2A |
| 2927 | CHRNA5 |
| 2928 | CPLANE1 |
| 2929 | RPL13 |
| 2930 | ABCE1 |
| 2931 | LZTR1 |
| 2932 | RBM10 |
| 2933 | ERCC8-AS1 |
| 2934 | PLCB1 |
| 2935 | PLAAT3 |
| 2936 | SOCS3 |
| 2937 | NRP1 |
| 2938 | H1-5 |
| 2939 | EPHA4 |
| 2940 | PGBD3 |
| 2941 | GJA5 |
| 2942 | KIF7 |
| 2943 | GMNN |
| 2944 | FOLH1 |
| 2945 | CGA |
| 2946 | PRX |
| 2947 | ELAC2 |
| 2948 | SCARB2 |
| 2949 | CORIN |
| 2950 | IL23R |
| 2951 | PPARD |
| 2952 | TAC1 |
| 2953 | DNAJC19 |
| 2954 | SPIB |
| 2955 | BICD2 |
| 2956 | CCL22 |
| 2957 | PIGY |
| 2958 | NDUFB10 |
| 2959 | MT-TL2 |
| 2960 | MIR95 |
| 2961 | ADORA1 |
| 2962 | HTRA2 |
| 2963 | ERN1 |
| 2964 | SRRT |
| 2965 | SERPINI1 |
| 2966 | MSRA |
| 2967 | STH |
| 2968 | MORF4L2 |
| 2969 | MIR323A |
| 2970 | BLVRB |
| 2971 | PLS1 |
| 2972 | UGP2 |
| 2973 | OSM |
| 2974 | COL12A1 |
| 2975 | ATXN10 |
| 2976 | WDR81 |
| 2977 | CEP120 |
| 2978 | DIS3L2 |
| 2979 | RPS6KA3 |
| 2980 | CYP51A1 |
| 2981 | LSM2 |
| 2982 | PDYN |
| 2983 | LCT |
| 2984 | PHOX2B |
| 2985 | PARP10 |
| 2986 | CPS1 |
| 2987 | KCNT2 |
| 2988 | COPS4 |
| 2989 | NSF |
| 2990 | S1PR2 |
| 2991 | CS |
| 2992 | ATP2A1 |
| 2993 | RIN2 |
| 2994 | CTRC |
| 2995 | USP48 |
| 2996 | DRD4 |
| 2997 | KDM5B |
| 2998 | RPS23 |
| 2999 | CXCL1 |
| 3000 | AGRN |
| 3001 | IL16 |
| 3002 | SLC25A10 |
| 3003 | SASS6 |
| 3004 | SLC39A7 |
| 3005 | SLC19A3 |
| 3006 | NAT10 |
| 3007 | OTOF |
| 3008 | TNFRSF13B |
| 3009 | COX6A1 |
| 3010 | FHL2 |
| 3011 | GSN |
| 3012 | WHRN |
| 3013 | RPS6KB1 |
| 3014 | CLCN6 |
| 3015 | MIR24-2 |
| 3016 | CENPA |
| 3017 | CYTOR |
| 3018 | ITGB6 |
| 3019 | USF1 |
| 3020 | BCAT2 |
| 3021 | FABP1 |
| 3022 | SRP19 |
| 3023 | MAN1B1 |
| 3024 | OXT |
| 3025 | NUP98 |
| 3026 | MIR202 |
| 3027 | NF2 |
| 3028 | CDC16 |
| 3029 | INA |
| 3030 | AXDND1 |
| 3031 | KRT5 |
| 3032 | HNRNPUL1 |
| 3033 | IRGM |
| 3034 | TTC19 |
| 3035 | LOC111674477 |
| 3036 | MAML1 |
| 3037 | NDUFA12 |
| 3038 | MAP2K4 |
| 3039 | NOS1AP |
| 3040 | EPOR |
| 3041 | TRIP13 |
| 3042 | MGME1 |
| 3043 | H3C14 |
| 3044 | NEDD4 |
| 3045 | ALDH6A1 |
| 3046 | WWC1 |
| 3047 | NAT1 |
| 3048 | DTNA |
| 3049 | CEACAM6 |
| 3050 | PPP1R10 |
| 3051 | PRPF31 |
| 3052 | MIR208B |
| 3053 | PROP1 |
| 3054 | HSPA1B |
| 3055 | NDUFB7 |
| 3056 | EXOSC10 |
| 3057 | SMARCA1 |
| 3058 | NUDC |
| 3059 | DAB1 |
| 3060 | MIR584 |
| 3061 | H2AC21 |
| 3062 | CLCA4 |
| 3063 | H2BC14 |
| 3064 | DVL2 |
| 3065 | DCAF17 |
| 3066 | ZNF638 |
| 3067 | PRPF3 |
| 3068 | KLF6 |
| 3069 | MYL9 |
| 3070 | SULT1A1 |
| 3071 | ALDOA |
| 3072 | EIF3B |
| 3073 | ALOX15 |
| 3074 | MMP8 |
| 3075 | RHD |
| 3076 | CHAMP1 |
| 3077 | CSN1S1 |
| 3078 | TAB2 |
| 3079 | PADI2 |
| 3080 | CRIPTO |
| 3081 | DNAAF11 |
| 3082 | KIF1B |
| 3083 | PTH1R |
| 3084 | MIR33B |
| 3085 | PGAP3 |
| 3086 | AGK |
| 3087 | KLRK1 |
| 3088 | PABPN1 |
| 3089 | H3C13 |
| 3090 | ALG1 |
| 3091 | DGKE |
| 3092 | HTR2A |
| 3093 | SERAC1 |
| 3094 | SGCD |
| 3095 | GATA6 |
| 3096 | MIR499A |
| 3097 | PMPCA |
| 3098 | CIDEA |
| 3099 | OMG |
| 3100 | GTPBP3 |
| 3101 | H2AC1 |
| 3102 | VANGL1 |
| 3103 | HIVEP2 |
| 3104 | COQ5 |
| 3105 | TNFSF15 |
| 3106 | SKAP2 |
| 3107 | CD247 |
| 3108 | MIR20B |
| 3109 | MIR301A |
| 3110 | SGCB |
| 3111 | AREG |
| 3112 | BDP1 |
| 3113 | TRIP11 |
| 3114 | WDR45 |
| 3115 | USP45 |
| 3116 | H2BC17 |
| 3117 | SLC6A14 |
| 3118 | TXNRD1 |
| 3119 | H2AC12 |
| 3120 | NR1I3 |
| 3121 | LOC654780 |
| 3122 | C1R |
| 3123 | DISP1 |
| 3124 | IFIT1 |
| 3125 | HMGN1 |
| 3126 | TRIM47 |
| 3127 | MLXIPL |
| 3128 | VRK2 |
| 3129 | LRP6 |
| 3130 | ARHGAP35 |
| 3131 | SRP68 |
| 3132 | TTF2 |
| 3133 | ANKH |
| 3134 | SNX14 |
| 3135 | PPP2R5D |
| 3136 | GJC1 |
| 3137 | SLC40A1 |
| 3138 | GRSF1 |
| 3139 | AZGP1 |
| 3140 | ABCC4 |
| 3141 | NSDHL |
| 3142 | ACVR1 |
| 3143 | FMNL1 |
| 3144 | PRKACA |
| 3145 | CYP20A1 |
| 3146 | KLC1 |
| 3147 | SLC37A4 |
| 3148 | SLC4A1 |
| 3149 | H2AC15 |
| 3150 | DRD3 |
| 3151 | GJA8 |
| 3152 | PDPN |
| 3153 | CCNA1 |
| 3154 | BRF1 |
| 3155 | TWIST1 |
| 3156 | CDC40 |
| 3157 | ACTN3 |
| 3158 | CCR1 |
| 3159 | WWP1 |
| 3160 | NSUN2 |
| 3161 | VAC14 |
| 3162 | HINT1 |
| 3163 | PPIL1 |
| 3164 | FGF1 |
| 3165 | TBC1D20 |
| 3166 | RHOBTB2 |
| 3167 | LPIN2 |
| 3168 | MIR99B |
| 3169 | ITGAE |
| 3170 | SNHG4 |
| 3171 | LGALS1 |
| 3172 | SLC6A2 |
| 3173 | FDX2 |
| 3174 | MAD2L1 |
| 3175 | TCP1 |
| 3176 | ANAPC1 |
| 3177 | SIL1 |
| 3178 | DCTN4 |
| 3179 | TCOF1 |
| 3180 | NFIX |
| 3181 | EIF4A1 |
| 3182 | UBE3C |
| 3183 | SDR9C7 |
| 3184 | ADCY10 |
| 3185 | KIF4A |
| 3186 | TPPP |
| 3187 | TBR1 |
| 3188 | PPOX |
| 3189 | EXOSC8 |
| 3190 | BMPR1A |
| 3191 | SIX6 |
| 3192 | MIR32 |
| 3193 | GABRB1 |
| 3194 | ISCU |
| 3195 | BCKDHB |
| 3196 | DDHD1 |
| 3197 | POMP |
| 3198 | CFHR1 |
| 3199 | ATP8A2 |
| 3200 | FTL |
| 3201 | RRP12 |
| 3202 | TGFB3 |
| 3203 | ASF1A |
| 3204 | CD86 |
| 3205 | MTPAP |
| 3206 | MIR100HG |
| 3207 | ATP6V0A2 |
| 3208 | TBC1D23 |
| 3209 | TUSC7 |
| 3210 | PXDN |
| 3211 | SPATA33 |
| 3212 | MT-TM |
| 3213 | FRAS1 |
| 3214 | LRSAM1 |
| 3215 | TIAM1 |
| 3216 | AMFR |
| 3217 | SCN11A |
| 3218 | TAF9 |
| 3219 | CTU2 |
| 3220 | NEUROD1 |
| 3221 | CYB5R3 |
| 3222 | ASS1 |
| 3223 | SNRNP200 |
| 3224 | RPSA |
| 3225 | MIR31HG |
| 3226 | NEB |
| 3227 | HYLS1 |
| 3228 | SNHG5 |
| 3229 | SUPV3L1 |
| 3230 | SOX3 |
| 3231 | DPF2 |
| 3232 | RPL27A |
| 3233 | EDEM3 |
| 3234 | NNT |
| 3235 | ARG1 |
| 3236 | AFP |
| 3237 | SIK1 |
| 3238 | HNRNPL |
| 3239 | MIR302A |
| 3240 | KPNA2 |
| 3241 | ECE1 |
| 3242 | AICDA |
| 3243 | MIR19B1 |
| 3244 | ZMIZ1 |
| 3245 | WAPL |
| 3246 | ZFHX3 |
| 3247 | SLF1 |
| 3248 | CSGALNACT1 |
| 3249 | TBX1 |
| 3250 | CRY1 |
| 3251 | ERLIN1 |
| 3252 | PHOX2A |
| 3253 | NDUFA13 |
| 3254 | CALB2 |
| 3255 | SLC39A13 |
| 3256 | RAB18 |
| 3257 | THY1 |
| 3258 | RACK1 |
| 3259 | MRPS16 |
| 3260 | FGFRL1 |
| 3261 | SLK |
| 3262 | GABBR1 |
| 3263 | NEU1 |
| 3264 | PLD3 |
| 3265 | MCCC1 |
| 3266 | DAO |
| 3267 | LRRC37A4P |
| 3268 | MAPK8IP1P1 |
| 3269 | ASH2L |
| 3270 | DDOST |
| 3271 | CHRNG |
| 3272 | H3-7 |
| 3273 | EEF1A1 |
| 3274 | OGA |
| 3275 | FAT1 |
| 3276 | FZD2 |
| 3277 | TRAPPC10 |
| 3278 | CELSR3 |
| 3279 | TNPO3 |
| 3280 | HNRNPUL2-BSCL2 |
| 3281 | MIR425 |
| 3282 | F2R |
| 3283 | ANKHD1 |
| 3284 | LIAS |
| 3285 | TALDO1 |
| 3286 | UTRN |
| 3287 | SLC7A11 |
| 3288 | MCCC2 |
| 3289 | TNNT1 |
| 3290 | MPI |
| 3291 | HNRNPA3 |
| 3292 | RIPK3 |
| 3293 | TCEA1 |
| 3294 | DSC3 |
| 3295 | HIBCH |
| 3296 | ASIP |
| 3297 | CYP7A1 |
| 3298 | COX14 |
| 3299 | DYNC2H1 |
| 3300 | PRR12 |
| 3301 | PMP2 |
| 3302 | MED12L |
| 3303 | HESX1 |
| 3304 | NSRP1 |
| 3305 | TRPV3 |
| 3306 | SPTLC2 |
| 3307 | EMC1 |
| 3308 | CAMK2G |
| 3309 | RPL22 |
| 3310 | RPS24 |
| 3311 | SNTA1 |
| 3312 | SPIDR |
| 3313 | PLEKHG1 |
| 3314 | CACNA1G-AS1 |
| 3315 | ENDOG |
| 3316 | MTIF3 |
| 3317 | MIR17HG |
| 3318 | THBS4 |
| 3319 | CRY2 |
| 3320 | NTS |
| 3321 | RNU6-1 |
| 3322 | CLP1 |
| 3323 | GRIA3 |
| 3324 | NUP93 |
| 3325 | PHF6 |
| 3326 | CPOX |
| 3327 | SEMA3A |
| 3328 | FOXH1 |
| 3329 | EBP |
| 3330 | TBXT |
| 3331 | H6PD |
| 3332 | MIR1275 |
| 3333 | STMN1 |
| 3334 | LOC126862860 |
| 3335 | SUN2 |
| 3336 | HGFAC |
| 3337 | SNCB |
| 3338 | CRLS1 |
| 3339 | VAMP2 |
| 3340 | RPS10 |
| 3341 | DHX37 |
| 3342 | DHX58 |
| 3343 | ANK1 |
| 3344 | IDO1 |
| 3345 | GAS8 |
| 3346 | FLT4 |
| 3347 | SFTPA1 |
| 3348 | NR0B1 |
| 3349 | HNF1A-AS1 |
| 3350 | CALCOCO2 |
| 3351 | TAPBP |
| 3352 | RB1CC1 |
| 3353 | PSORS1C1 |
| 3354 | HDAC7 |
| 3355 | MUC2 |
| 3356 | KNL1 |
| 3357 | MYEF2 |
| 3358 | SRY |
| 3359 | DYNLL1 |
| 3360 | LAMA4 |
| 3361 | IBSP |
| 3362 | TTLL4 |
| 3363 | GPAM |
| 3364 | MXI1 |
| 3365 | GATAD2A |
| 3366 | BTF3 |
| 3367 | TPH1 |
| 3368 | SEC24D |
| 3369 | GCH1 |
| 3370 | USH1C |
| 3371 | VTRNA1-1 |
| 3372 | KANSL1-AS1 |
| 3373 | GATA4 |
| 3374 | EIF6 |
| 3375 | NPHP3-ACAD11 |
| 3376 | GSTO1 |
| 3377 | SHMT1 |
| 3378 | NEFM |
| 3379 | OXA1L |
| 3380 | HPCA |
| 3381 | FOXA2 |
| 3382 | CDK5RAP3 |
| 3383 | LOC107032825 |
| 3384 | LOC129929053 |
| 3385 | H3C4 |
| 3386 | EXOSC1 |
| 3387 | IGBP1 |
| 3388 | ATP2B2 |
| 3389 | PRMT5 |
| 3390 | HSPB6 |
| 3391 | MST1 |
| 3392 | MIR302C |
| 3393 | SLC32A1 |
| 3394 | IL10RB |
| 3395 | SLC30A10 |
| 3396 | NGFR |
| 3397 | RCC1 |
| 3398 | ZGRF1 |
| 3399 | FOXL1 |
| 3400 | MTG2 |
| 3401 | PMF1 |
| 3402 | PRG2 |
| 3403 | ARPC2 |
| 3404 | RPS16 |
| 3405 | MTNR1B |
| 3406 | DNAJB4 |
| 3407 | FGF9 |
| 3408 | FGG |
| 3409 | AMN |
| 3410 | VSX1 |
| 3411 | UROD |
| 3412 | POU1F1 |
| 3413 | SMAD1 |
| 3414 | CD5 |
| 3415 | TMEM222 |
| 3416 | LINGO1 |
| 3417 | EXTL3 |
| 3418 | NDUFB8 |
| 3419 | MED23 |
| 3420 | MIR18B |
| 3421 | SLC6A9 |
| 3422 | POU2AF1 |
| 3423 | SYT1 |
| 3424 | POLE4 |
| 3425 | COLGALT1 |
| 3426 | SPRED1 |
| 3427 | MS4A1 |
| 3428 | PUS1 |
| 3429 | PTS |
| 3430 | PARL |
| 3431 | PICK1 |
| 3432 | H2AC11 |
| 3433 | ARSG |
| 3434 | CXCL13 |
| 3435 | CCT7 |
| 3436 | COP1 |
| 3437 | ADAMTS2 |
| 3438 | DOCK2 |
| 3439 | NDUFA11 |
| 3440 | GFOD3P |
| 3441 | HLA-E |
| 3442 | DPYSL2 |
| 3443 | REEP2 |
| 3444 | DDX59 |
| 3445 | MACROD2 |
| 3446 | LINC01394 |
| 3447 | GJB3 |
| 3448 | PAK2 |
| 3449 | SNORD44 |
| 3450 | LPO |
| 3451 | MAML2 |
| 3452 | CHRM3 |
| 3453 | MBD2 |
| 3454 | NNMT |
| 3455 | PCM1 |
| 3456 | RPL31 |
| 3457 | ITGA2 |
| 3458 | PLAGL1 |
| 3459 | ESPN |
| 3460 | ABCC3 |
| 3461 | PGAP2 |
| 3462 | GJC3 |
| 3463 | VEGFC |
| 3464 | CYB5A |
| 3465 | PRKCG |
| 3466 | MIR216A |
| 3467 | TPR |
| 3468 | PDE5A |
| 3469 | GFI1 |
| 3470 | COX10 |
| 3471 | ALKBH5 |
| 3472 | MC2R |
| 3473 | KLK6 |
| 3474 | STAR |
| 3475 | LOC126653351 |
| 3476 | TRAF2 |
| 3477 | LOC117125594 |
| 3478 | F10 |
| 3479 | XPO5 |
| 3480 | SLC12A1 |
| 3481 | RIPOR2 |
| 3482 | TRERNA1 |
| 3483 | PPFIA1 |
| 3484 | PHYH |
| 3485 | CAMTA1 |
| 3486 | SF3B3 |
| 3487 | DEFB4A |
| 3488 | NAIP |
| 3489 | RPS6 |
| 3490 | KMT2B |
| 3491 | PPP1CC |
| 3492 | MYH10 |
| 3493 | CFHR3 |
| 3494 | SLC34A3 |
| 3495 | UQCC3 |
| 3496 | MED27 |
| 3497 | LMO2 |
| 3498 | ABCC11 |
| 3499 | PIGT |
| 3500 | CTNND2 |
| 3501 | TCTN3 |
| 3502 | DCAKD |
| 3503 | RNF217-AS1 |
| 3504 | PIGV |
| 3505 | FRMD4A |
| 3506 | CASZ1 |
| 3507 | DANCR |
| 3508 | TRIOBP |
| 3509 | CHCHD10 |
| 3510 | GJA4 |
| 3511 | SLITRK2 |
| 3512 | MT-TE |
| 3513 | CORO1A |
| 3514 | FOXF1 |
| 3515 | DNAJA1 |
| 3516 | TAF7 |
| 3517 | SLC22A12 |
| 3518 | IAPP |
| 3519 | GSTO2 |
| 3520 | RNF170 |
| 3521 | SLC25A17 |
| 3522 | TUBB6 |
| 3523 | AMT |
| 3524 | ATPAF2 |
| 3525 | UBE2E2 |
| 3526 | MSTN |
| 3527 | PTX3 |
| 3528 | DUSP1 |
| 3529 | GTF2IRD1 |
| 3530 | GPR35 |
| 3531 | SERBP1 |
| 3532 | UVRAG |
| 3533 | CKAP4 |
| 3534 | AGTR2 |
| 3535 | BCL11A |
| 3536 | GLP1R |
| 3537 | RECK |
| 3538 | SAA4 |
| 3539 | CAMK2A |
| 3540 | CASQ1 |
| 3541 | MIR455 |
| 3542 | SMARCD1 |
| 3543 | PGF |
| 3544 | BLID |
| 3545 | TPH2 |
| 3546 | PRUNE1 |
| 3547 | YBX3 |
| 3548 | DGCR8 |
| 3549 | CRIPT |
| 3550 | SALL4 |
| 3551 | GLE1 |
| 3552 | MLYCD |
| 3553 | TTI1 |
| 3554 | DNAJB6 |
| 3555 | FNDC3B |
| 3556 | PAICS |
| 3557 | GLUD1 |
| 3558 | CRYBA4 |
| 3559 | FADS1 |
| 3560 | ATP6AP1 |
| 3561 | KIAA0319L |
| 3562 | NTF4 |
| 3563 | TAF2 |
| 3564 | MIR9-2 |
| 3565 | EEF1D |
| 3566 | MIR409 |
| 3567 | MRPL44 |
| 3568 | GRHPR |
| 3569 | TSEN2 |
| 3570 | ADAMTSL1 |
| 3571 | ABCC10 |
| 3572 | H1-1 |
| 3573 | PIEZO1 |
| 3574 | REST |
| 3575 | PARD3 |
| 3576 | ALDOC |
| 3577 | WNT4 |
| 3578 | EXOC7 |
| 3579 | SLC27A1 |
| 3580 | UNC45A |
| 3581 | ARHGAP27 |
| 3582 | FADS2 |
| 3583 | SRPX2 |
| 3584 | IRF9 |
| 3585 | TRN-GTT2-1 |
| 3586 | ZNF365 |
| 3587 | CCL27 |
| 3588 | GSTA1 |
| 3589 | CARD14 |
| 3590 | TCEA3 |
| 3591 | CNDP1 |
| 3592 | ADPRH |
| 3593 | NCSTN |
| 3594 | AKAP13 |
| 3595 | CTCF |
| 3596 | HEY2 |
| 3597 | PER2 |
| 3598 | TMEM216 |
| 3599 | CSPP1 |
| 3600 | SLC26A2 |
| 3601 | TIMM8A |
| 3602 | SUPT20H |
| 3603 | DIS3 |
| 3604 | CNNM2 |
| 3605 | DYNC2I1 |
| 3606 | KCNE1 |
| 3607 | TTC7A |
| 3608 | GPKOW |
| 3609 | HNRNPDL |
| 3610 | POLR2E |
| 3611 | IL1RAP |
| 3612 | PITX1 |
| 3613 | DNAJB11 |
| 3614 | TRIM27 |
| 3615 | TNR |
| 3616 | IFIT3 |
| 3617 | NDEL1 |
| 3618 | KPNA3 |
| 3619 | UBE4B |
| 3620 | QDPR |
| 3621 | SMC4 |
| 3622 | IL10RA |
| 3623 | MYL2 |
| 3624 | ZNF142 |
| 3625 | COL4A2-AS1 |
| 3626 | TBL1X |
| 3627 | SLC7A5 |
| 3628 | ITGAX |
| 3629 | PYGL |
| 3630 | CALM1 |
| 3631 | CLPX |
| 3632 | MIR129-1 |
| 3633 | SELPLG |
| 3634 | LAP3 |
| 3635 | MUC16 |
| 3636 | PTAFR |
| 3637 | PPP2R3C |
| 3638 | VIPAS39 |
| 3639 | WDR5 |
| 3640 | CAND1 |
| 3641 | CCHCR1 |
| 3642 | IL12RB2 |
| 3643 | PLA2G4A |
| 3644 | CC2D2A |
| 3645 | GET4 |
| 3646 | KCTD7 |
| 3647 | MPDU1 |
| 3648 | PTGDR |
| 3649 | RF00017-2421 |
| 3650 | MIR423 |
| 3651 | MSX2 |
| 3652 | LORICRIN |
| 3653 | E2F3 |
| 3654 | ALDH1A2 |
| 3655 | ARHGEF2 |
| 3656 | LEMD3 |
| 3657 | PPP1CB |
| 3658 | PART1 |
| 3659 | MIPEP |
| 3660 | PRDX6 |
| 3661 | SHROOM3 |
| 3662 | TRPA1 |
| 3663 | APCS |
| 3664 | RETREG1 |
| 3665 | COCH |
| 3666 | DBH |
| 3667 | CASC15 |
| 3668 | ITM2B |
| 3669 | TFB1M |
| 3670 | DHX8 |
| 3671 | ALDH4A1 |
| 3672 | POU3F1 |
| 3673 | CD58 |
| 3674 | MT-RNR2 |
| 3675 | SLC30A9 |
| 3676 | ARL3 |
| 3677 | PDE3B |
| 3678 | MBNL1 |
| 3679 | MAD1L1 |
| 3680 | MTHFD1L |
| 3681 | MIR125B2 |
| 3682 | BLNK |
| 3683 | ZC3H4 |
| 3684 | VPS35 |
| 3685 | REPS1 |
| 3686 | SLC12A3 |
| 3687 | GANAB |
| 3688 | AMPD1 |
| 3689 | LSS |
| 3690 | KIAA0319 |
| 3691 | MBTPS1 |
| 3692 | SLC9A1 |
| 3693 | ZBTB32 |
| 3694 | H2BC12L |
| 3695 | HSPA1L |
| 3696 | SDHC |
| 3697 | MCFD2 |
| 3698 | GREM1 |
| 3699 | CD33 |
| 3700 | LEPQTL1 |
| 3701 | IL23A |
| 3702 | UBE2S |
| 3703 | ALOX15B |
| 3704 | ATG16L1 |
| 3705 | DLD |
| 3706 | DNAJC30 |
| 3707 | ATIC |
| 3708 | SF3A1 |
| 3709 | TRAPPC12 |
| 3710 | GMNC |
| 3711 | GCGR |
| 3712 | TPRKB |
| 3713 | CDH13 |
| 3714 | CLEC4E |
| 3715 | GPNMB |
| 3716 | MARVELD2 |
| 3717 | HCCS |
| 3718 | PFDN1 |
| 3719 | DEPDC5 |
| 3720 | MED13L |
| 3721 | MT-TN |
| 3722 | CD69 |
| 3723 | MAVS |
| 3724 | ACY1 |
| 3725 | HEXB |
| 3726 | CHIC2 |
| 3727 | MAPRE2 |
| 3728 | NKX2-2 |
| 3729 | EPCAM |
| 3730 | UBIAD1 |
| 3731 | CALML5 |
| 3732 | DDX23 |
| 3733 | CCL17 |
| 3734 | KRT7 |
| 3735 | PHF20 |
| 3736 | DMBT1 |
| 3737 | OGDHL |
| 3738 | ELAVL1 |
| 3739 | GSK3A |
| 3740 | CDC42BPB |
| 3741 | FBF1 |
| 3742 | CARF |
| 3743 | SLC16A1 |
| 3744 | CD52 |
| 3745 | CACNA1G |
| 3746 | ATP8A1 |
| 3747 | MDH1 |
| 3748 | PITRM1 |
| 3749 | MYL12A |
| 3750 | APPL2 |
| 3751 | PMF1-BGLAP |
| 3752 | COL4A2-AS2 |
| 3753 | ELOC |
| 3754 | NFATC2 |
| 3755 | APBB3 |
| 3756 | TRIM56 |
| 3757 | CSK |
| 3758 | IFNA21 |
| 3759 | CCR3 |
| 3760 | DNAJA3 |
| 3761 | FAS-AS1 |
| 3762 | SERPIND1 |
| 3763 | HNMT |
| 3764 | TCF12 |
| 3765 | LRRC37A |
| 3766 | CCR4 |
| 3767 | MAP3K5 |
| 3768 | STX6 |
| 3769 | DGCR5 |
| 3770 | ALDH5A1 |
| 3771 | TNFRSF13C |
| 3772 | DAPK1 |
| 3773 | ETFB |
| 3774 | TBL2 |
| 3775 | LYRM4 |
| 3776 | CNR2 |
| 3777 | HS2ST1 |
| 3778 | ZNF462 |
| 3779 | WNK1 |
| 3780 | ROBO3 |
| 3781 | CCDC47 |
| 3782 | AQP5 |
| 3783 | WT1-AS |
| 3784 | SLC29A3 |
| 3785 | ITIH4 |
| 3786 | DCD |
| 3787 | ST3GAL4 |
| 3788 | A2M |
| 3789 | CCL19 |
| 3790 | NRF1 |
| 3791 | SCN1A-AS1 |
| 3792 | ADIPOR2 |
| 3793 | RAN |
| 3794 | AP2S1 |
| 3795 | PLA2R1 |
| 3796 | RNH1 |
| 3797 | AQP1 |
| 3798 | IDE |
| 3799 | THAP1 |
| 3800 | GPC3 |
| 3801 | MIR664A |
| 3802 | PCARE |
| 3803 | HLA-DRA |
| 3804 | SENP1 |
| 3805 | WDR4 |
| 3806 | PRDM2 |
| 3807 | MSX1 |
| 3808 | TUBA1B |
| 3809 | MACF1 |
| 3810 | MRPS7 |
| 3811 | CASC2 |
| 3812 | H2AZ2 |
| 3813 | DND1P1 |
| 3814 | CCT3 |
| 3815 | IFT140 |
| 3816 | JAGN1 |
| 3817 | HOXA1 |
| 3818 | LOC129931597 |
| 3819 | SNCG |
| 3820 | DNAJA2 |
| 3821 | CRK |
| 3822 | TRAP1 |
| 3823 | SLC6A1 |
| 3824 | SMAD9 |
| 3825 | POLR1A |
| 3826 | LFNG |
| 3827 | MACROH2A2 |
| 3828 | LHX3 |
| 3829 | LARS1 |
| 3830 | HTR2C |
| 3831 | ACADSB |
| 3832 | PRRT2 |
| 3833 | UQCRC2 |
| 3834 | CA3 |
| 3835 | CBX8 |
| 3836 | MAFB |
| 3837 | ADAM12 |
| 3838 | VAPB |
| 3839 | THRA |
| 3840 | RFT1 |
| 3841 | GOLGA2 |
| 3842 | EPAS1 |
| 3843 | GSTM2 |
| 3844 | ZCCHC8 |
| 3845 | H3-5 |
| 3846 | NDN |
| 3847 | IFNA6 |
| 3848 | TRPC6 |
| 3849 | ARHGAP42 |
| 3850 | HEY1 |
| 3851 | TRIM25 |
| 3852 | SLC4A2 |
| 3853 | CDIPT |
| 3854 | MAPK8IP1 |
| 3855 | FDPS |
| 3856 | OSGEP |
| 3857 | NR2F1-AS1 |
| 3858 | NAT8L |
| 3859 | PI3 |
| 3860 | STX8 |
| 3861 | FCHO2 |
| 3862 | BST2 |
| 3863 | SBF2-AS1 |
| 3864 | MT2A |
| 3865 | MAPKAPK5 |
| 3866 | TRAPPC13 |
| 3867 | MED24 |
| 3868 | CIB1 |
| 3869 | YJU2 |
| 3870 | MIR422A |
| 3871 | AQP2 |
| 3872 | MTAP |
| 3873 | RBM7 |
| 3874 | S100A1 |
| 3875 | UNC119 |
| 3876 | HMX1 |
| 3877 | TRIM5 |
| 3878 | TDO2 |
| 3879 | MIR193B |
| 3880 | TSEN15 |
| 3881 | SHOC2 |
| 3882 | PLCH1 |
| 3883 | GDF6 |
| 3884 | RAB38 |
| 3885 | ZKSCAN8 |
| 3886 | ZKSCAN4 |
| 3887 | LOC106050102 |
| 3888 | DUSP19 |
| 3889 | IGFBP7 |
| 3890 | BDKRB2 |
| 3891 | MIP |
| 3892 | PSMF1 |
| 3893 | STS |
| 3894 | MSRB3 |
| 3895 | CD22 |
| 3896 | FOXJ1 |
| 3897 | FERMT3 |
| 3898 | PHF11 |
| 3899 | NFIB |
| 3900 | UBE2O |
| 3901 | LRRC8A |
| 3902 | ILDR1 |
| 3903 | CFL1 |
| 3904 | STT3A |
| 3905 | CHRM2 |
| 3906 | AIP |
| 3907 | CYP24A1 |
| 3908 | CCKAR |
| 3909 | PNMA2 |
| 3910 | SRD5A1 |
| 3911 | ATOX1 |
| 3912 | MAGT1 |
| 3913 | ITCH |
| 3914 | VSX2 |
| 3915 | LBP |
| 3916 | FXR2 |
| 3917 | DNAJB1 |
| 3918 | CERS3 |
| 3919 | LMBRD1 |
| 3920 | XBP1 |
| 3921 | MINAR2 |
| 3922 | TPPP3 |
| 3923 | CD79B |
| 3924 | VAX1 |
| 3925 | KIF5B |
| 3926 | CALM3 |
| 3927 | PTCD2 |
| 3928 | PRPSAP1 |
| 3929 | NCK1 |
| 3930 | FCRL3 |
| 3931 | RHBDF2 |
| 3932 | TMEM237 |
| 3933 | TNPO1 |
| 3934 | DAZAP1 |
| 3935 | SETD7 |
| 3936 | TAF4 |
| 3937 | ADIPOR1 |
| 3938 | GAL |
| 3939 | JPH3 |
| 3940 | HIF1A-AS2 |
| 3941 | CXCL11 |
| 3942 | RNPC3 |
| 3943 | SOX17 |
| 3944 | GCAT |
| 3945 | PLCE1 |
| 3946 | NEXMIF |
| 3947 | AGPS |
| 3948 | DEFB1 |
| 3949 | SPHK1 |
| 3950 | GMPPA |
| 3951 | AMBRA1 |
| 3952 | PCGF2 |
| 3953 | CXCR1 |
| 3954 | SLC35C1 |
| 3955 | IFNA5 |
| 3956 | TBX21 |
| 3957 | LGALS7 |
| 3958 | SVBP |
| 3959 | GNRHR |
| 3960 | FABP12 |
| 3961 | ALCAM |
| 3962 | ILF2 |
| 3963 | LOC126860933 |
| 3964 | RN7SL1 |
| 3965 | CAPZB |
| 3966 | PARP4 |
| 3967 | FSCN2 |
| 3968 | SOAT1 |
| 3969 | RNY1 |
| 3970 | GNPAT |
| 3971 | PABPC1 |
| 3972 | RPS27 |
| 3973 | CDK3 |
| 3974 | UBE3B |
| 3975 | ILK |
| 3976 | IL31 |
| 3977 | RPL28 |
| 3978 | LMAN1 |
| 3979 | CLIC1 |
| 3980 | TNFRSF12A |
| 3981 | NALT1 |
| 3982 | MIR376A1 |
| 3983 | GATA6-AS1 |
| 3984 | AOC1 |
| 3985 | PSIP1 |
| 3986 | FASTKD5 |
| 3987 | CNTN1 |
| 3988 | CTNND1 |
| 3989 | MT-TR |
| 3990 | FAM117B |
| 3991 | IFIT2 |
| 3992 | RNY3 |
| 3993 | PDGFA |
| 3994 | TMEM150B |
| 3995 | COL4A6 |
| 3996 | ACBD4 |
| 3997 | GFPT1 |
| 3998 | SLC34A2 |
| 3999 | PTBP1 |
| 4000 | LUCAT1 |
| 4001 | COL7A1 |
| 4002 | SUMF2 |
| 4003 | YIF1B |
| 4004 | MTIF2 |
| 4005 | LOC126861242 |
| 4006 | SNORA40 |
| 4007 | MIR599 |
| 4008 | IFNL3 |
| 4009 | ACOX3 |
| 4010 | CISH |
| 4011 | IFNA4 |
| 4012 | TMEM163 |
| 4013 | AGTPBP1 |
| 4014 | DLK1 |
| 4015 | HSF4 |
| 4016 | RIT1 |
| 4017 | CALML3 |
| 4018 | SPTBN2 |
| 4019 | TFPI |
| 4020 | RBP3 |
| 4021 | EXT2 |
| 4022 | IFT27 |
| 4023 | SLC25A3 |
| 4024 | ADD2 |
| 4025 | CAVIN1 |
| 4026 | RPS7 |
| 4027 | LOC130068781 |
| 4028 | SMG8 |
| 4029 | PANK4 |
| 4030 | DROSHA |
| 4031 | E2F4 |
| 4032 | GZMA |
| 4033 | SEMA4D |
| 4034 | PLOD3 |
| 4035 | HAGLR |
| 4036 | TMPRSS4 |
| 4037 | LHX4 |
| 4038 | GAP43 |
| 4039 | PROCR |
| 4040 | PMPCB |
| 4041 | ACO1 |
| 4042 | PHB2 |
| 4043 | MYOG |
| 4044 | MCM5 |
| 4045 | ALG6 |
| 4046 | GTF3C1 |
| 4047 | CAV2 |
| 4048 | CCNC |
| 4049 | PNPLA2 |
| 4050 | SETD1A |
| 4051 | EOGT |
| 4052 | ANO10 |
| 4053 | WNT7B |
| 4054 | ACTBL2 |
| 4055 | PSMG2 |
| 4056 | IL21 |
| 4057 | PCBP4 |
| 4058 | NTRK3 |
| 4059 | SPINK5 |
| 4060 | WBP2 |
| 4061 | MYT1 |
| 4062 | STRA6 |
| 4063 | PDXK |
| 4064 | ALDH16A1 |
| 4065 | TCEA2 |
| 4066 | TOMM20 |
| 4067 | CAMLG |
| 4068 | MRPL12 |
| 4069 | VPS41 |
| 4070 | QSOX1 |
| 4071 | PGD |
| 4072 | HTR7 |
| 4073 | HK2 |
| 4074 | CAMSAP1 |
| 4075 | SIGLEC1 |
| 4076 | CLDN5 |
| 4077 | CHRM1 |
| 4078 | MT-TD |
| 4079 | AS3MT |
| 4080 | TUBA4A |
| 4081 | ALX3 |
| 4082 | VAMP7 |
| 4083 | CLEC16A |
| 4084 | SRD5A3 |
| 4085 | CX3CL1 |
| 4086 | MRPS27 |
| 4087 | MSN |
| 4088 | CLK2 |
| 4089 | SRGAP1 |
| 4090 | RNF217 |
| 4091 | ATP5F1C |
| 4092 | MYT1L |
| 4093 | CISD2 |
| 4094 | PGRMC1 |
| 4095 | CD70 |
| 4096 | UQCC2 |
| 4097 | IFNA8 |
| 4098 | IFNA17 |
| 4099 | IGFBP4 |
| 4100 | UCN |
| 4101 | EIF3F |
| 4102 | SYNM |
| 4103 | SERPINB7 |
| 4104 | ACOX2 |
| 4105 | NADK2 |
| 4106 | ZCCHC14 |
| 4107 | RPS6KA5 |
| 4108 | KCNAB2 |
| 4109 | DENND5A |
| 4110 | COL8A1 |
| 4111 | IFNA13 |
| 4112 | PTPRD |
| 4113 | ADAM10 |
| 4114 | LINC00963 |
| 4115 | EPS15L1 |
| 4116 | PIGU |
| 4117 | IFNA14 |
| 4118 | TGS1 |
| 4119 | SEC31A |
| 4120 | WNT11 |
| 4121 | HEXIM1 |
| 4122 | TRIM71 |
| 4123 | SLC35A1 |
| 4124 | PTK2B |
| 4125 | FALEC |
| 4126 | LAMA3 |
| 4127 | BAIAP2L2 |
| 4128 | ULK1 |
| 4129 | PROKR2 |
| 4130 | EVPL |
| 4131 | IFNA10 |
| 4132 | LIN28B |
| 4133 | PHETA1 |
| 4134 | ADCYAP1 |
| 4135 | HNRNPD |
| 4136 | MRM2 |
| 4137 | FOXK1 |
| 4138 | CSTF1 |
| 4139 | MTX2 |
| 4140 | S100A6 |
| 4141 | CD47 |
| 4142 | PTDSS1 |
| 4143 | YTHDF1 |
| 4144 | MIR574 |
| 4145 | LOC108663996 |
| 4146 | SLC9A7 |
| 4147 | PIK3R3 |
| 4148 | KIAA0753 |
| 4149 | MEIS1 |
| 4150 | CAMP |
| 4151 | SGTB |
| 4152 | BCAS3 |
| 4153 | PTRH2 |
| 4154 | GOLGB1 |
| 4155 | G2E3 |
| 4156 | SPI1 |
| 4157 | SMNDC1 |
| 4158 | SYT2 |
| 4159 | ZBTB18 |
| 4160 | NCAPD2 |
| 4161 | MITD1 |
| 4162 | TRPM2 |
| 4163 | MED17 |
| 4164 | PRKCQ |
| 4165 | DAAM2 |
| 4166 | MRPS26 |
| 4167 | GFRA2 |
| 4168 | ECEL1 |
| 4169 | CYFIP1 |
| 4170 | TFB2M |
| 4171 | OBI1 |
| 4172 | NAGS |
| 4173 | PAWR |
| 4174 | TMPRSS2 |
| 4175 | TEFM |
| 4176 | E2F2 |
| 4177 | EIF5A |
| 4178 | DAOA |
| 4179 | FABP5 |
| 4180 | MRPS9 |
| 4181 | PDE4B |
| 4182 | GRB10 |
| 4183 | TRPM7 |
| 4184 | SAMD9 |
| 4185 | COL25A1 |
| 4186 | TMPRSS5 |
| 4187 | ZNF516 |
| 4188 | MMADHC |
| 4189 | MEG8 |
| 4190 | HTR1B |
| 4191 | VPS13C |
| 4192 | SEC23IP |
| 4193 | B3GALT6 |
| 4194 | COIL |
| 4195 | BBS1 |
| 4196 | UNC93B1 |
| 4197 | AGBL1 |
| 4198 | OTUD7A |
| 4199 | MAST1 |
| 4200 | IFNA7 |
| 4201 | FBXO7 |
| 4202 | DYNC2I2 |
| 4203 | TCF19 |
| 4204 | LDLRAP1 |
| 4205 | MCU |
| 4206 | EFEMP2 |
| 4207 | TNFSF12 |
| 4208 | TAF13 |
| 4209 | HYOU1 |
| 4210 | HPX |
| 4211 | ZNHIT3 |
| 4212 | FSHR |
| 4213 | C3AR1 |
| 4214 | LGR5 |
| 4215 | SHPK |
| 4216 | SUN1 |
| 4217 | LOC130056175 |
| 4218 | IFNA16 |
| 4219 | H3C2 |
| 4220 | IFT74 |
| 4221 | MAEA |
| 4222 | SIRPA |
| 4223 | GATB |
| 4224 | CPSF1 |
| 4225 | EXT1 |
| 4226 | PRKAB1 |
| 4227 | MIR582 |
| 4228 | ZNF592 |
| 4229 | RPS28 |
| 4230 | GRAMD2B |
| 4231 | CRMA |
| 4232 | CNOT3 |
| 4233 | UBA2 |
| 4234 | BRD3 |
| 4235 | DNASE1L1 |
| 4236 | SCHLAP1 |
| 4237 | ZKSCAN3 |
| 4238 | ZSCAN31 |
| 4239 | LUZP2 |
| 4240 | FRMPD4 |
| 4241 | DOLK |
| 4242 | VTI1A |
| 4243 | RCL1 |
| 4244 | FXR1 |
| 4245 | DNM3 |
| 4246 | SLAMF1 |
| 4247 | ARHGEF12 |
| 4248 | NR1D1 |
| 4249 | MCPH1-AS1 |
| 4250 | PDIA3 |
| 4251 | VPS33B |
| 4252 | PPIA |
| 4253 | SECISBP2 |
| 4254 | DGAT1 |
| 4255 | P2RX5 |
| 4256 | SP110 |
| 4257 | OMA1 |
| 4258 | NUMB |
| 4259 | ABCF2 |
| 4260 | SC5D |
| 4261 | SKI |
| 4262 | SNHG16 |
| 4263 | PLEK |
| 4264 | NCOA6 |
| 4265 | TCF25 |
| 4266 | MIR502 |
| 4267 | CFHR4 |
| 4268 | AAGAB |
| 4269 | XK |
| 4270 | ATAD3B |
| 4271 | ENPP2 |
| 4272 | MCRS1 |
| 4273 | CD2AP |
| 4274 | PNLIP |
| 4275 | WDR55 |
| 4276 | DDX54 |
| 4277 | NUP88 |
| 4278 | SEC61A1 |
| 4279 | UPF3B |
| 4280 | RPS18 |
| 4281 | SGCA |
| 4282 | PIBF1 |
| 4283 | CLASP2 |
| 4284 | ARHGEF10 |
| 4285 | WDR37 |
| 4286 | EEFSEC |
| 4287 | ZFP57 |
| 4288 | UBE2C |
| 4289 | STRN |
| 4290 | LNPK |
| 4291 | SPRED2 |
| 4292 | MRPS25 |
| 4293 | ST3GAL3 |
| 4294 | CGGBP1 |
| 4295 | TBX2 |
| 4296 | CXCL5 |
| 4297 | MAPT-AS1 |
| 4298 | CHRDL1 |
| 4299 | FSCN1 |
| 4300 | GRK5 |
| 4301 | DCLK1 |
| 4302 | HOMER1 |
| 4303 | CARMN |
| 4304 | H1-10 |
| 4305 | GRIA1 |
| 4306 | NEGR1 |
| 4307 | DDX47 |
| 4308 | NR4A1 |
| 4309 | EGFL8 |
| 4310 | GLI1 |
| 4311 | CKAP5 |
| 4312 | MIR137HG |
| 4313 | GPAA1 |
| 4314 | PNKD |
| 4315 | MEPE |
| 4316 | TXN2 |
| 4317 | SRSF7 |
| 4318 | CDC23 |
| 4319 | VEGFB |
| 4320 | ACAD8 |
| 4321 | FUT2 |
| 4322 | THORLNC |
| 4323 | DUSP10 |
| 4324 | TFPT |
| 4325 | ASPN |
| 4326 | MCAM |
| 4327 | PCSK1 |
| 4328 | SRCAP |
| 4329 | DMP1 |
| 4330 | VWA1 |
| 4331 | CPQ |
| 4332 | TMEM126A |
| 4333 | GTPBP4 |
| 4334 | RRAS2 |
| 4335 | ALYREF |
| 4336 | VPS33A |
| 4337 | IFT52 |
| 4338 | TTBK2 |
| 4339 | TACC3 |
| 4340 | IL37 |
| 4341 | ZC3H11A |
| 4342 | RMST |
| 4343 | ARHGDIA |
| 4344 | RUSC2 |
| 4345 | MRC1 |
| 4346 | SLIT2 |
| 4347 | NOTCH4 |
| 4348 | GABRB3 |
| 4349 | SCD5 |
| 4350 | HACD3 |
| 4351 | PRKD1 |
| 4352 | AGO2 |
| 4353 | ZEB2-AS1 |
| 4354 | GRM1 |
| 4355 | CXCR5 |
| 4356 | CD9 |
| 4357 | SFRP4 |
| 4358 | VPS50 |
| 4359 | FGF14 |
| 4360 | ITGA7 |
| 4361 | MT3 |
| 4362 | PNPO |
| 4363 | MYPN |
| 4364 | CDIN1 |
| 4365 | VAMP8 |
| 4366 | TLN1 |
| 4367 | DIDO1 |
| 4368 | SLC25A44 |
| 4369 | RASGRP3 |
| 4370 | AMPH |
| 4371 | NUTM2B-AS1 |
| 4372 | LOXHD1 |
| 4373 | ZBP1 |
| 4374 | B9D2 |
| 4375 | SNRNP40 |
| 4376 | IFT80 |
| 4377 | SETD5 |
| 4378 | RPLP1 |
| 4379 | SERPINF2 |
| 4380 | ST7-OT3 |
| 4381 | PPP3R1 |
| 4382 | PHF13 |
| 4383 | PFKP |
| 4384 | AP1S1 |
| 4385 | NAP1L1 |
| 4386 | PRPF4 |
| 4387 | EIF4G2 |
| 4388 | RPL27 |
| 4389 | ITGAD |
| 4390 | EPPK1 |
| 4391 | MBD5 |
| 4392 | FAM98B |
| 4393 | PLD1 |
| 4394 | ABCB10 |
| 4395 | PDE4A |
| 4396 | AEBP1 |
| 4397 | NME2 |
| 4398 | NINJ2 |
| 4399 | HDLBP |
| 4400 | ZC3HC1 |
| 4401 | IL34 |
| 4402 | SETDB1 |
| 4403 | MIR590 |
| 4404 | FUZ |
| 4405 | ADAMTS9-AS2 |
| 4406 | QRSL1 |
| 4407 | MIR9-3HG |
| 4408 | TMPRSS6 |
| 4409 | CREB5 |
| 4410 | CALCRL |
| 4411 | MAML3 |
| 4412 | BIK |
| 4413 | MADCAM1 |
| 4414 | HAR1A |
| 4415 | KIF15 |
| 4416 | VAV1 |
| 4417 | STAMBP |
| 4418 | NUP210 |
| 4419 | SH3PXD2B |
| 4420 | FOXC2-AS1 |
| 4421 | CES1 |
| 4422 | SP140 |
| 4423 | PHACTR1 |
| 4424 | CRYBB3 |
| 4425 | SORT1 |
| 4426 | MIR526B |
| 4427 | LOC126805877 |
| 4428 | CDS1 |
| 4429 | GOLPH3 |
| 4430 | STIM1 |
| 4431 | EPS15 |
| 4432 | CHRD |
| 4433 | RBBP7 |
| 4434 | SART1 |
| 4435 | DPH2 |
| 4436 | LMAN2 |
| 4437 | PRKCE |
| 4438 | CTNNBL1 |
| 4439 | TCTN2 |
| 4440 | GLYCTK |
| 4441 | H2BC1 |
| 4442 | GAST |
| 4443 | PHC2 |
| 4444 | PRPF6 |
| 4445 | CHN1 |
| 4446 | RNF13 |
| 4447 | TCHH |
| 4448 | TAT |
| 4449 | MMAA |
| 4450 | PTGIS |
| 4451 | GJB4 |
| 4452 | NBR1 |
| 4453 | ST8SIA2 |
| 4454 | LUZP1 |
| 4455 | APOA4 |
| 4456 | SAP30BP |
| 4457 | ZNF292 |
| 4458 | HAR1B |
| 4459 | SLC30A5 |
| 4460 | ACADL |
| 4461 | NR5A1 |
| 4462 | MED15 |
| 4463 | LRAT |
| 4464 | WLS |
| 4465 | MT-TA |
| 4466 | UPP1 |
| 4467 | ARPC1B |
| 4468 | LNX1 |
| 4469 | PLTP |
| 4470 | PIK3C2G |
| 4471 | NAF1 |
| 4472 | SPATA32 |
| 4473 | XRN2 |
| 4474 | LOC130058543 |
| 4475 | SERPINE2 |
| 4476 | MKLN1 |
| 4477 | ATP10D |
| 4478 | TFF2 |
| 4479 | EXOC8 |
| 4480 | CYP4F2 |
| 4481 | SELENOH |
| 4482 | HDAC5 |
| 4483 | SNHG7 |
| 4484 | TSNAX |
| 4485 | GID8 |
| 4486 | PSTPIP1 |
| 4487 | KIRREL1 |
| 4488 | WDR1 |
| 4489 | UMPS |
| 4490 | PCID2 |
| 4491 | EIF3H |
| 4492 | PLCG1 |
| 4493 | IAH1 |
| 4494 | FZD6 |
| 4495 | MYBBP1A |
| 4496 | CTSS |
| 4497 | IFT81 |
| 4498 | DDR1 |
| 4499 | SERPINA7 |
| 4500 | ROBO4 |
| 4501 | WDR35 |
| 4502 | FREM2 |
| 4503 | ADORA2B |
| 4504 | NOVA1 |
| 4505 | SEC16A |
| 4506 | MSMB |
| 4507 | GRIK4 |
| 4508 | MX2 |
| 4509 | PIK3R4 |
| 4510 | ANG |
| 4511 | TMEM138 |
| 4512 | DUSP9 |
| 4513 | VDAC2 |
| 4514 | RHOD |
| 4515 | CCBE1 |
| 4516 | DEF8 |
| 4517 | SLC18A2 |
| 4518 | FAM98A |
| 4519 | UBAP1 |
| 4520 | MSL2 |
| 4521 | RAB11A |
| 4522 | PHAX |
| 4523 | FAM111A |
| 4524 | IMPDH1 |
| 4525 | ANLN |
| 4526 | METTL5 |
| 4527 | ARRB1 |
| 4528 | MPZL1 |
| 4529 | AMER1 |
| 4530 | LRRK1 |
| 4531 | SCFD1 |
| 4532 | GCLM |
| 4533 | RTN2 |
| 4534 | B9D1 |
| 4535 | COQ3 |
| 4536 | RSAD2 |
| 4537 | PBX2 |
| 4538 | AKR1D1 |
| 4539 | PRDX4 |
| 4540 | PDS5A |
| 4541 | MIR1972-1 |
| 4542 | RAB4B-EGLN2 |
| 4543 | LRP8 |
| 4544 | PPP4R3B |
| 4545 | NFE2L3 |
| 4546 | BRD1 |
| 4547 | GABRD |
| 4548 | SP2 |
| 4549 | MBNL2 |
| 4550 | CHD6 |
| 4551 | RP2 |
| 4552 | ADGRB3 |
| 4553 | MYOM2 |
| 4554 | MYO18A |
| 4555 | KANK1 |
| 4556 | ATP9A |
| 4557 | RPS12 |
| 4558 | DIO2 |
| 4559 | DENND1B |
| 4560 | HSPB3 |
| 4561 | NCKAP1 |
| 4562 | VAPA |
| 4563 | IRF6 |
| 4564 | CALU |
| 4565 | BRSK2 |
| 4566 | AOX1 |
| 4567 | RBBP6 |
| 4568 | ADGRB1 |
| 4569 | FGGY |
| 4570 | NOVA2 |
| 4571 | DOCK1 |
| 4572 | CCT8 |
| 4573 | PAX7 |
| 4574 | PVR |
| 4575 | PAPPA-AS1 |
| 4576 | FER |
| 4577 | SHOX |
| 4578 | FRA10AC1 |
| 4579 | WIPI2 |
| 4580 | SMAD5 |
| 4581 | SLIT1 |
| 4582 | TCEAL1 |
| 4583 | AP2B1 |
| 4584 | RAB8A |
| 4585 | LMX1A |
| 4586 | UQCRB |
| 4587 | COG1 |
| 4588 | ACTR3B |
| 4589 | RFX7 |
| 4590 | CCL21 |
| 4591 | CD2 |
| 4592 | TOP1MT |
| 4593 | PDZD7 |
| 4594 | TFE3 |
| 4595 | STMN2 |
| 4596 | CREB3 |
| 4597 | TAGLN2 |
| 4598 | PTF1A |
| 4599 | KRT3 |
| 4600 | UGT2B7 |
| 4601 | SGCE |
| 4602 | ORM2 |
| 4603 | ELL |
| 4604 | PPAT |
| 4605 | DLX3 |
| 4606 | INCENP |
| 4607 | CAPN5 |
| 4608 | NOG |
| 4609 | TUBGCP6 |
| 4610 | SCYL2 |
| 4611 | SLC4A11 |
| 4612 | ATP5F1B |
| 4613 | NOD1 |
| 4614 | HP1BP3 |
| 4615 | GNAI1 |
| 4616 | RNF11 |
| 4617 | CD24 |
| 4618 | PAX1 |
| 4619 | PDE6A |
| 4620 | SAFB |
| 4621 | TMEM30A |
| 4622 | TK1 |
| 4623 | FLOT1 |
| 4624 | ZNF366 |
| 4625 | SLC1A5 |
| 4626 | LOC109461477 |
| 4627 | TBC1D7 |
| 4628 | SGCG |
| 4629 | STAU1 |
| 4630 | SSBP3 |
| 4631 | RBM38 |
| 4632 | ADGRG6 |
| 4633 | CEP41 |
| 4634 | TRIM38 |
| 4635 | CCL7 |
| 4636 | NELFE |
| 4637 | STK19 |
| 4638 | PWAR5 |
| 4639 | POLR2C |
| 4640 | DPP10 |
| 4641 | MIR136 |
| 4642 | VPS13A |
| 4643 | PRKCSH |
| 4644 | ATP6V0A4 |
| 4645 | RPL38 |
| 4646 | MYSM1 |
| 4647 | SEC23A |
| 4648 | MMP23B |
| 4649 | ZC3HAV1 |
| 4650 | LANCL2 |
| 4651 | DMAC2L |
| 4652 | HELZ2 |
| 4653 | MIR29B2 |
| 4654 | B4GALT1 |
| 4655 | ETS2 |
| 4656 | SEMA4A |
| 4657 | NASP |
| 4658 | MMEL1 |
| 4659 | ADARB2 |
| 4660 | PANK1 |
| 4661 | ZRANB2 |
| 4662 | UQCRC1 |
| 4663 | TMCC3 |
| 4664 | SAE1 |
| 4665 | TRIM23 |
| 4666 | SFXN2 |
| 4667 | PTPRF |
| 4668 | ENSG00000262539 |
| 4669 | STK36 |
| 4670 | SLC1A1 |
| 4671 | RNASE1 |
| 4672 | PLA2G1B |
| 4673 | RPL37A |
| 4674 | NOL9 |
| 4675 | ABI2 |
| 4676 | STT3B |
| 4677 | DNAJB2 |
| 4678 | GK |
| 4679 | ACSL3 |
| 4680 | PIPOX |
| 4681 | PDLIM1 |
| 4682 | ARSK |
| 4683 | COLEC10 |
| 4684 | LOC109504728 |
| 4685 | DOCK5 |
| 4686 | LOC100507346 |
| 4687 | MTA2 |
| 4688 | PER3 |
| 4689 | WNT10A |
| 4690 | EIF3A |
| 4691 | DBNDD1 |
| 4692 | S100A4 |
| 4693 | PSORS1C3 |
| 4694 | PPP1R18 |
| 4695 | LIMA1 |
| 4696 | BLOC1S3 |
| 4697 | NRXN2 |
| 4698 | CBY1 |
| 4699 | CD180 |
| 4700 | RCN2 |
| 4701 | PEG3 |
| 4702 | SLC18A1 |
| 4703 | ITPR2 |
| 4704 | SHARPIN |
| 4705 | GRIN2C |
| 4706 | AQP3 |
| 4707 | DNAH11 |
| 4708 | TAF8 |
| 4709 | IFNK |
| 4710 | CSTB |
| 4711 | GCN1 |
| 4712 | DHODH |
| 4713 | TCERG1 |
| 4714 | SSPN |
| 4715 | MT-TT |
| 4716 | EIF4G3 |
| 4717 | HID1 |
| 4718 | ALDH3B1 |
| 4719 | NPEPPS |
| 4720 | CTSF |
| 4721 | CRNDE |
| 4722 | WTAP |
| 4723 | CALML4 |
| 4724 | GHRH |
| 4725 | FAM120A |
| 4726 | MIR376B |
| 4727 | SMG9 |
| 4728 | RDH8 |
| 4729 | GRM3 |
| 4730 | MIA3 |
| 4731 | LACTB |
| 4732 | ZSCAN12 |
| 4733 | NUDCD3 |
| 4734 | RGS7BP |
| 4735 | RASA2 |
| 4736 | SCYL1 |
| 4737 | CENPH |
| 4738 | HGD |
| 4739 | PXDNL |
| 4740 | ACKR2 |
| 4741 | LNCTAM34A |
| 4742 | GTF2B |
| 4743 | ARL13B |
| 4744 | SNORD43 |
| 4745 | PNISR |
| 4746 | RNF135 |
| 4747 | MED4 |
| 4748 | GABARAPL2 |
| 4749 | CHERP |
| 4750 | BAMBI |
| 4751 | ALX1 |
| 4752 | SLC10A2 |
| 4753 | RPLP2 |
| 4754 | KDM3B |
| 4755 | AFF2 |
| 4756 | LOC126861615 |
| 4757 | MYOT |
| 4758 | CYP8B1 |
| 4759 | NFS1 |
| 4760 | FCN3 |
| 4761 | FTSJ3 |
| 4762 | SLC66A2 |
| 4763 | CAPZA1 |
| 4764 | NOP2 |
| 4765 | IRF2BPL |
| 4766 | CEACAM5 |
| 4767 | CAPN15 |
| 4768 | NHLH2 |
| 4769 | STX5 |
| 4770 | EEF1G |
| 4771 | TET1 |
| 4772 | NPR2 |
| 4773 | CCNK |
| 4774 | LOC129390683 |
| 4775 | CDH5 |
| 4776 | FOXE3 |
| 4777 | ENDOV |
| 4778 | SPINT1 |
| 4779 | HSD3B7 |
| 4780 | CHRNA7 |
| 4781 | ZNF668 |
| 4782 | CACNA1S |
| 4783 | DDR2 |
| 4784 | MCHR2 |
| 4785 | ZCCHC3 |
| 4786 | USP44 |
| 4787 | ORMDL3 |
| 4788 | SLCO2A1 |
| 4789 | IFNGR2 |
| 4790 | POLR3GL |
| 4791 | PRLR |
| 4792 | PACS1 |
| 4793 | COX7B |
| 4794 | WDR47 |
| 4795 | BTC |
| 4796 | AP1G1 |
| 4797 | RHOG |
| 4798 | POLA2 |
| 4799 | MAST3 |
| 4800 | RUNX1T1 |
| 4801 | RPS5 |
| 4802 | ADK |
| 4803 | SPNS2 |
| 4804 | APCDD1 |
| 4805 | MDCMP |
| 4806 | ACAD11 |
| 4807 | AVPR2 |
| 4808 | GNLY |
| 4809 | KRT9 |
| 4810 | CYC1 |
| 4811 | MID1 |
| 4812 | SLC31A1 |
| 4813 | FABP6 |
| 4814 | KRT12 |
| 4815 | AIFM2 |
| 4816 | KGD4 |
| 4817 | FBLN1 |
| 4818 | CCDC6 |
| 4819 | CFAP43 |
| 4820 | CWC27 |
| 4821 | EPHA1 |
| 4822 | TRD-GTC9-1 |
| 4823 | GNAT1 |
| 4824 | CCDC115 |
| 4825 | EIF3L |
| 4826 | NUAK2 |
| 4827 | RAC3 |
| 4828 | RBFOX3 |
| 4829 | RAPGEF2 |
| 4830 | OASL |
| 4831 | BLMH |
| 4832 | LRATD1 |
| 4833 | LINC01191 |
| 4834 | NLN |
| 4835 | PRDM1 |
| 4836 | DNAAF2 |
| 4837 | TUBGCP5 |
| 4838 | G3BP2 |
| 4839 | INVS |
| 4840 | UTS2 |
| 4841 | JPX |
| 4842 | NPHP3 |
| 4843 | MMAB |
| 4844 | CARMIL2 |
| 4845 | LRFN4 |
| 4846 | MAGEL2 |
| 4847 | FCER2 |
| 4848 | PTCD1 |
| 4849 | MRPL49 |
| 4850 | NCR1 |
| 4851 | DPYD-AS1 |
| 4852 | TENT4B |
| 4853 | HOXA-AS3 |
| 4854 | AUTS2 |
| 4855 | SLC30A6 |
| 4856 | MTERF1 |
| 4857 | SYMPK |
| 4858 | LIMK1 |
| 4859 | SNU13 |
| 4860 | PYCR3 |
| 4861 | TUBB1 |
| 4862 | CST7 |
| 4863 | CFC1 |
| 4864 | ADAMTSL4 |
| 4865 | MIR92A2 |
| 4866 | FIS1 |
| 4867 | MBD1 |
| 4868 | LUC7L3 |
| 4869 | SPINT2 |
| 4870 | TMEM218 |
| 4871 | MIR485 |
| 4872 | MICU2 |
| 4873 | HCG22 |
| 4874 | SNX10 |
| 4875 | GTPBP1 |
| 4876 | SPATA7 |
| 4877 | RRAGC |
| 4878 | DNAJC1 |
| 4879 | ITLN1 |
| 4880 | ANAPC5 |
| 4881 | TCEAL3 |
| 4882 | MVB12A |
| 4883 | JPH1 |
| 4884 | CD5L |
| 4885 | RNF14 |
| 4886 | TACR1 |
| 4887 | KIF2B |
| 4888 | ARCN1 |
| 4889 | CSH1 |
| 4890 | HAAO |
| 4891 | CKAP2 |
| 4892 | GPC4 |
| 4893 | ZBTB24 |
| 4894 | DNAL4 |
| 4895 | PCDH12 |
| 4896 | CD48 |
| 4897 | ADRM1 |
| 4898 | KCNH5 |
| 4899 | RSPRY1 |
| 4900 | EVL |
| 4901 | PROZ |
| 4902 | HLA-DQB2 |
| 4903 | SHLD3 |
| 4904 | NOX5 |
| 4905 | IPW |
| 4906 | IPO11 |
| 4907 | PRODH |
| 4908 | ENAH |
| 4909 | WDFY3 |
| 4910 | M6PR |
| 4911 | TBCEL |
| 4912 | HOXA11-AS |
| 4913 | EIF4A2 |
| 4914 | ATXN2L |
| 4915 | ATP6V0D1 |
| 4916 | MRPL36 |
| 4917 | DIP2B |
| 4918 | HNRNPA0 |
| 4919 | MYO1D |
| 4920 | SNRPE |
| 4921 | PTPRJ |
| 4922 | RPL21 |
| 4923 | CRYM |
| 4924 | KDM8 |
| 4925 | GGT5 |
| 4926 | AFG3L1P |
| 4927 | FKBP4 |
| 4928 | LONP2 |
| 4929 | INHA |
| 4930 | DUX4 |
| 4931 | ATL3 |
| 4932 | EIF3C |
| 4933 | CSH2 |
| 4934 | RPL34 |
| 4935 | HTR3A |
| 4936 | SEC13 |
| 4937 | MIR496 |
| 4938 | CIB2 |
| 4939 | B3GAT1 |
| 4940 | SYN1 |
| 4941 | TTYH3 |
| 4942 | TSR1 |
| 4943 | PIGK |
| 4944 | LOC130059818 |
| 4945 | HMGN2 |
| 4946 | LOC105369149 |
| 4947 | GTF2H2C |
| 4948 | CCL26 |
| 4949 | CDAN1 |
| 4950 | MIR1909 |
| 4951 | CXADR |
| 4952 | ZNF148 |
| 4953 | MRM3 |
| 4954 | CCAR1 |
| 4955 | LTBP3 |
| 4956 | SIM1 |
| 4957 | CENPK |
| 4958 | KLHL7 |
| 4959 | KMT5C |
| 4960 | IRX2-DT |
| 4961 | FBXO3 |
| 4962 | PIGS |
| 4963 | HLA-DOB |
| 4964 | CALM2 |
| 4965 | ARNT2 |
| 4966 | DAOA-AS1 |
| 4967 | ADGRE5 |
| 4968 | KLLN |
| 4969 | THRB |
| 4970 | BEND5 |
| 4971 | TIE1 |
| 4972 | FGF19 |
| 4973 | SMS |
| 4974 | IFNL1 |
| 4975 | HACL1 |
| 4976 | ASH1L |
| 4977 | BBS4 |
| 4978 | YLPM1 |
| 4979 | PPIH |
| 4980 | HSCB |
| 4981 | BSND |
| 4982 | BICC1 |
| 4983 | DMXL1 |
| 4984 | STK39 |
| 4985 | ADAMTS6 |
| 4986 | ANAPC2 |
| 4987 | PRPF40A |
| 4988 | RHOC |
| 4989 | LOC130006142 |
| 4990 | DDX55 |
| 4991 | RNMT |
| 4992 | NFYC |
| 4993 | ACAT1 |
| 4994 | LOC129993881 |
| 4995 | ACLY |
| 4996 | ZSWIM7 |
| 4997 | DPH6 |
| 4998 | PIGO |
| 4999 | BNC2 |
| 5000 | KIR2DL4 |
| 5001 | AGRP |
| 5002 | TCN1 |
| 5003 | MASP2 |
| 5004 | MAP2K7 |
| 5005 | DDX50 |
| 5006 | CCKBR |
| 5007 | SND1 |
| 5008 | RAB23 |
| 5009 | CLDN1 |
| 5010 | SLC25A53 |
| 5011 | MAP1LC3A |
| 5012 | IFNE |
| 5013 | MIR181D |
| 5014 | ENTREP1 |
| 5015 | GNAI3 |
| 5016 | PRKRA |
| 5017 | AQP9 |
| 5018 | PRP4K |
| 5019 | LRRC32 |
| 5020 | AGA |
| 5021 | RUFY1 |
| 5022 | LIPG |
| 5023 | APOA1-AS |
| 5024 | MANBA |
| 5025 | CDC123 |
| 5026 | LAG3 |
| 5027 | RPTOR |
| 5028 | COL8A2 |
| 5029 | HYAL1 |
| 5030 | CCDC85C |
| 5031 | TBX15 |
| 5032 | PAX8 |
| 5033 | SARS2 |
| 5034 | ATG14 |
| 5035 | DHX38 |
| 5036 | CA1 |
| 5037 | CCL20 |
| 5038 | LSM8 |
| 5039 | ODAD4 |
| 5040 | ODAD2 |
| 5041 | TOR1AIP1 |
| 5042 | MIR675 |
| 5043 | MAT2A |
| 5044 | GABARAPL1 |
| 5045 | TRAPPC2 |
| 5046 | SLC5A2 |
| 5047 | IFT25 |
| 5048 | SLAMF7 |
| 5049 | LTBR |
| 5050 | PDCD11 |
| 5051 | LUC7L |
| 5052 | F11R |
| 5053 | POSTN |
| 5054 | H2BW1 |
| 5055 | FST |
| 5056 | COX4I2 |
| 5057 | CEPT1 |
| 5058 | MUC5B |
| 5059 | SOCS2 |
| 5060 | NME1-NME2 |
| 5061 | DNAJC9 |
| 5062 | RALA |
| 5063 | PEX5L |
| 5064 | TRH |
| 5065 | PI4KB |
| 5066 | ATP6V1E1 |
| 5067 | RBCK1 |
| 5068 | PDPK1 |
| 5069 | MIR101-1 |
| 5070 | SLC51B |
| 5071 | ADGRF1 |
| 5072 | ABO |
| 5073 | MIR374B |
| 5074 | THADA |
| 5075 | ELF2 |
| 5076 | FYN |
| 5077 | GON7 |
| 5078 | APOM |
| 5079 | SLC16A8 |
| 5080 | ENSA |
| 5081 | RPN2 |
| 5082 | PPM1K |
| 5083 | MAP3K20 |
| 5084 | DDX56 |
| 5085 | IHH |
| 5086 | EMG1 |
| 5087 | RBPMS |
| 5088 | PHF3 |
| 5089 | WNT2 |
| 5090 | GHSR |
| 5091 | AK6 |
| 5092 | PRICKLE1 |
| 5093 | CD1A |
| 5094 | ACVR2A |
| 5095 | MED13 |
| 5096 | ITGA5 |
| 5097 | LRP1B |
| 5098 | SIK3 |
| 5099 | REG3A |
| 5100 | AMZ2P1 |
| 5101 | APLN |
| 5102 | EIF3E |
| 5103 | CLRN2 |
| 5104 | SCFD2 |
| 5105 | CA14 |
| 5106 | COPG2 |
| 5107 | ZFP91-CNTF |
| 5108 | DEGS2 |
| 5109 | CACNA1I |
| 5110 | PRPS2 |
| 5111 | F2RL1 |
| 5112 | TRIM66 |
| 5113 | LARGE2 |
| 5114 | COPB1 |
| 5115 | CHST14 |
| 5116 | CHST6 |
| 5117 | SREK1 |
| 5118 | SLC4A4 |
| 5119 | SULT2A1 |
| 5120 | MAX |
| 5121 | OPHN1 |
| 5122 | TRP-AGG2-6 |
| 5123 | MAP4 |
| 5124 | CILK1 |
| 5125 | MSI1 |
| 5126 | VPS35L |
| 5127 | DBF4B |
| 5128 | ULBP3 |
| 5129 | NOL11 |
| 5130 | CTSE |
| 5131 | ATP2B4 |
| 5132 | KCNC3 |
| 5133 | MED8 |
| 5134 | FBN2 |
| 5135 | HAS2-AS1 |
| 5136 | RXRG |
| 5137 | CYGB |
| 5138 | NRTN |
| 5139 | SOS2 |
| 5140 | KRIT1 |
| 5141 | DGAT2 |
| 5142 | RPIA |
| 5143 | GFRA1 |
| 5144 | RTN3 |
| 5145 | LRRC8E |
| 5146 | TFF3 |
| 5147 | CHRNB4 |
| 5148 | ROGDI |
| 5149 | ZMAT2 |
| 5150 | DEDD |
| 5151 | CNTROB |
| 5152 | CPVL |
| 5153 | GORAB |
| 5154 | TPK1 |
| 5155 | SIRT5 |
| 5156 | CWC22 |
| 5157 | ACKR3 |
| 5158 | IL1RL1 |
| 5159 | AKR1C1 |
| 5160 | AZIN2 |
| 5161 | TNFRSF17 |
| 5162 | PPP2R5A |
| 5163 | ESCO2 |
| 5164 | UQCRFS1 |
| 5165 | FZD3 |
| 5166 | CARTPT |
| 5167 | PLN |
| 5168 | SUGP1 |
| 5169 | RAE1 |
| 5170 | RBM42 |
| 5171 | EPB41 |
| 5172 | KIF13B |
| 5173 | WNK3 |
| 5174 | SLC44A4 |
| 5175 | RPL29 |
| 5176 | AP2A1 |
| 5177 | MANF |
| 5178 | MECR |
| 5179 | GABARAP |
| 5180 | RNASEH2B-AS1 |
| 5181 | LOC130009810 |
| 5182 | LAMTOR2 |
| 5183 | INPP5K |
| 5184 | TSPAN18 |
| 5185 | FCER1G |
| 5186 | SATB1 |
| 5187 | ANAPC11 |
| 5188 | PCAT2 |
| 5189 | TRPM8 |
| 5190 | CDYL |
| 5191 | CCS |
| 5192 | WDR19 |
| 5193 | SAA2 |
| 5194 | KCNA3 |
| 5195 | TNIK |
| 5196 | TREML2 |
| 5197 | OTOG |
| 5198 | SIPA1L2 |
| 5199 | EPHA3 |
| 5200 | CHMP4B |
| 5201 | TAGAP |
| 5202 | ERBIN |
| 5203 | DDX39A |
| 5204 | FOXP4 |
| 5205 | SLC25A38 |
| 5206 | KCNQ3 |
| 5207 | TAX1BP3 |
| 5208 | DDX20 |
| 5209 | GSDMD |
| 5210 | SLC11A2 |
| 5211 | TANGO2 |
| 5212 | PICSAR |
| 5213 | RRAS |
| 5214 | SCO1 |
| 5215 | MCM3AP-AS1 |
| 5216 | MAIP1 |
| 5217 | PLXNA1 |
| 5218 | ARVCF |
| 5219 | AP5S1 |
| 5220 | PDLIM5 |
| 5221 | ARHGAP26 |
| 5222 | JOSD2 |
| 5223 | SLC30A1 |
| 5224 | NHERF1 |
| 5225 | SLC19A2 |
| 5226 | ARID3A |
| 5227 | IPPK |
| 5228 | GABRA1 |
| 5229 | EFNB1 |
| 5230 | SARDH |
| 5231 | SP3 |
| 5232 | STAU2 |
| 5233 | LOC111365141 |
| 5234 | IQCA1 |
| 5235 | TNRC6B |
| 5236 | YTHDC2 |
| 5237 | IDH3B |
| 5238 | SMURF1 |
| 5239 | SLCO4A1 |
| 5240 | CCR6 |
| 5241 | MRM1 |
| 5242 | AKR1B10 |
| 5243 | LMF1 |
| 5244 | FKBP1A |
| 5245 | AZU1 |
| 5246 | AIPL1 |
| 5247 | CNGB1 |
| 5248 | DBN1 |
| 5249 | U2AF1L4 |
| 5250 | PEBP1 |
| 5251 | CYP2U1-AS1 |
| 5252 | HHAT |
| 5253 | VPS26A |
| 5254 | PDYN-AS1 |
| 5255 | KRT20 |
| 5256 | TLCD1 |
| 5257 | RARG |
| 5258 | ERV3-1 |
| 5259 | SF1 |
| 5260 | TPRN |
| 5261 | HOXD13 |
| 5262 | ADAMTS1 |
| 5263 | ZFAND6 |
| 5264 | CACNA2D2 |
| 5265 | TGDS |
| 5266 | MLN |
| 5267 | B4GALT7 |
| 5268 | MTDH |
| 5269 | SYNE4 |
| 5270 | DPEP1 |
| 5271 | TCTN1 |
| 5272 | LOC109504725 |
| 5273 | SF3B5 |
| 5274 | NPPC |
| 5275 | IRAK3 |
| 5276 | IGLON5 |
| 5277 | PKD2L1 |
| 5278 | SPR |
| 5279 | CADM1 |
| 5280 | KCNC2 |
| 5281 | DCAF8 |
| 5282 | CRLF2 |
| 5283 | BBS12 |
| 5284 | GGCX |
| 5285 | PNMT |
| 5286 | TRMT112 |
| 5287 | CCN1 |
| 5288 | PA2G4 |
| 5289 | SLC38A8 |
| 5290 | GDF1 |
| 5291 | BCL7B |
| 5292 | SPEF2 |
| 5293 | NPY4R |
| 5294 | IDH3A |
| 5295 | DDX3Y |
| 5296 | LOC130006506 |
| 5297 | MIR124-1HG |
| 5298 | CHM |
| 5299 | UBD |
| 5300 | ADAM33 |
| 5301 | CCDC174 |
| 5302 | CSMD1 |
| 5303 | NPRL2 |
| 5304 | IL21R |
| 5305 | FURIN |
| 5306 | CCP110 |
| 5307 | SYVN1 |
| 5308 | FGFR4 |
| 5309 | GJD2 |
| 5310 | EPB41L3 |
| 5311 | FTMT |
| 5312 | GHRHR |
| 5313 | MAPT-IT1 |
| 5314 | ACOT8 |
| 5315 | VPS52 |
| 5316 | KLHL15 |
| 5317 | IFI35 |
| 5318 | OPRL1 |
| 5319 | ERVW-1 |
| 5320 | CHMP1B |
| 5321 | C12orf43 |
| 5322 | TNPO2 |
| 5323 | SLC35D1 |
| 5324 | CSNK1A1L |
| 5325 | INTS8 |
| 5326 | MPRIP |
| 5327 | RAPH1 |
| 5328 | MT-TC |
| 5329 | TRAPPC6B |
| 5330 | LINC00261 |
| 5331 | LRRC37A3 |
| 5332 | PHC3 |
| 5333 | ARSI |
| 5334 | GATA5 |
| 5335 | UTP18 |
| 5336 | LINC01133 |
| 5337 | LSR |
| 5338 | RBFOX2 |
| 5339 | CGB5 |
| 5340 | IL13RA1 |
| 5341 | SOX6 |
| 5342 | SMARCD2 |
| 5343 | FAU |
| 5344 | PMEL |
| 5345 | MYOM1 |
| 5346 | MED7 |
| 5347 | MFF |
| 5348 | MYO1B |
| 5349 | IFI44 |
| 5350 | IFI44L |
| 5351 | PPWD1 |
| 5352 | MIR9-2HG |
| 5353 | SNX8 |
| 5354 | PSPH |
| 5355 | TYSND1 |
| 5356 | TFEC |
| 5357 | TAF10 |
| 5358 | DOK7 |
| 5359 | CEP104 |
| 5360 | UBE2K |
| 5361 | ZSCAN26 |
| 5362 | S100A7 |
| 5363 | IGHM |
| 5364 | KIF21B |
| 5365 | GRINA |
| 5366 | TXNDC15 |
| 5367 | ITGA3 |
| 5368 | DOHH |
| 5369 | AZIN1 |
| 5370 | UPK1A |
| 5371 | PPIF |
| 5372 | SORBS3 |
| 5373 | IL32 |
| 5374 | BAIAP2L1 |
| 5375 | GPATCH8 |
| 5376 | SLC20A2 |
| 5377 | SRF |
| 5378 | DISC1FP1 |
| 5379 | CARHSP1 |
| 5380 | RBM5-AS1 |
| 5381 | PLCD3 |
| 5382 | FAM149B1 |
| 5383 | CAMK1D |
| 5384 | MIR135A1 |
| 5385 | SLC25A32 |
| 5386 | TBPL1 |
| 5387 | LCA5 |
| 5388 | ATP11C |
| 5389 | UTP14C |
| 5390 | EGFLAM |
| 5391 | RAB32 |
| 5392 | WWP2 |
| 5393 | COG8 |
| 5394 | LMO7 |
| 5395 | SLC27A2 |
| 5396 | HLA-DRB6 |
| 5397 | CRYBA1 |
| 5398 | GRWD1 |
| 5399 | BAG1 |
| 5400 | MIR1-1 |
| 5401 | IFNAR2-IL10RB |
| 5402 | CLEC6A |
| 5403 | TXNL4A |
| 5404 | LARS2-AS1 |
| 5405 | SLC2A5 |
| 5406 | PHYKPL |
| 5407 | MIR379 |
| 5408 | CTTNBP2 |
| 5409 | PAK4 |
| 5410 | KCTD9 |
| 5411 | KIR2DL1 |
| 5412 | ELP2 |
| 5413 | DEPDC1B |
| 5414 | RAB27B |
| 5415 | POU3F3 |
| 5416 | SLC33A1 |
| 5417 | TTLL6 |
| 5418 | ENSG00000276376 |
| 5419 | SLC39A5 |
| 5420 | ARPC3 |
| 5421 | GMPS |
| 5422 | C8B |
| 5423 | RAX |
| 5424 | E2F5 |
| 5425 | PANX1 |
| 5426 | PDE10A |
| 5427 | ESX1 |
| 5428 | RSPH4A |
| 5429 | RAB3A |
| 5430 | TAF6 |
| 5431 | TRA2A |
| 5432 | NAV2 |
| 5433 | MARCHF3 |
| 5434 | PHKG2 |
| 5435 | DIMT1 |
| 5436 | ELOB |
| 5437 | MIR421 |
| 5438 | POU3F4 |
| 5439 | SNORD94 |
| 5440 | ERP44 |
| 5441 | MIR1915 |
| 5442 | MARCKSL1 |
| 5443 | POLR3H |
| 5444 | BYSL |
| 5445 | RPL36 |
| 5446 | PGAM1 |
| 5447 | BFSP2 |
| 5448 | LINC01116 |
| 5449 | CAMTA2 |
| 5450 | RBM28 |
| 5451 | CACNA1H |
| 5452 | GMDS |
| 5453 | MTO1 |
| 5454 | SRBD1 |
| 5455 | TNFRSF18 |
| 5456 | FUBP1 |
| 5457 | MIR135B |
| 5458 | LTA4H |
| 5459 | FFAR1 |
| 5460 | NSMF |
| 5461 | MYCNOS |
| 5462 | LOC126806913 |
| 5463 | YEATS2 |
| 5464 | CTH |
| 5465 | SERF1A |
| 5466 | THUMPD1 |
| 5467 | CDH11 |
| 5468 | MVD |
| 5469 | MED20 |
| 5470 | PAEP |
| 5471 | CALCR |
| 5472 | ANOS1 |
| 5473 | ZNF621 |
| 5474 | CNTLN |
| 5475 | MYBL2 |
| 5476 | UROS |
| 5477 | TBC1D8B |
| 5478 | FYCO1 |
| 5479 | RNF112 |
| 5480 | XYLT2 |
| 5481 | PAF1 |
| 5482 | CDC42EP2 |
| 5483 | FGF3 |
| 5484 | RAB6A |
| 5485 | SLC26A1 |
| 5486 | GON4L |
| 5487 | BAIAP3 |
| 5488 | FRMPD1 |
| 5489 | GABRG3 |
| 5490 | CD1D |
| 5491 | NIPSNAP2 |
| 5492 | ANKRD17 |
| 5493 | VPS37D |
| 5494 | LOC126862097 |
| 5495 | RAPSN |
| 5496 | COBL |
| 5497 | SLC39A4 |
| 5498 | GDF5 |
| 5499 | CLCF1 |
| 5500 | BACH1 |
| 5501 | AHSP |
| 5502 | PTGR1 |
| 5503 | NKX1-1 |
| 5504 | NT5C3A |
| 5505 | ICMT |
| 5506 | YTHDC1 |
| 5507 | HES1 |
| 5508 | MMP16 |
| 5509 | JADRR |
| 5510 | CLIP2 |
| 5511 | PPY |
| 5512 | SAMD4B |
| 5513 | PDCD1LG2 |
| 5514 | DAPK2 |
| 5515 | DIRAS3 |
| 5516 | IGKC |
| 5517 | MTMR3 |
| 5518 | LDAH |
| 5519 | PRKCI |
| 5520 | ZCCHC18 |
| 5521 | ATAD3C |
| 5522 | TRIM36 |
| 5523 | LSM14A |
| 5524 | LINC01194 |
| 5525 | NRL |
| 5526 | DDRGK1 |
| 5527 | PRDM13 |
| 5528 | EMB |
| 5529 | CD1C |
| 5530 | ADAM9 |
| 5531 | PITX3 |
| 5532 | POM121L12 |
| 5533 | FOXD1 |
| 5534 | AHCYL1 |
| 5535 | SEMA3C |
| 5536 | HOXA11 |
| 5537 | C1QL1 |
| 5538 | EXOSC7 |
| 5539 | BMS1 |
| 5540 | SART3 |
| 5541 | LIN9 |
| 5542 | ZC3H13 |
| 5543 | DNMT3L |
| 5544 | KIF20B |
| 5545 | OSGIN1 |
| 5546 | CPSF2 |
| 5547 | LOC130002651 |
| 5548 | GPANK1 |
| 5549 | FKBP15 |
| 5550 | RPL24 |
| 5551 | PELI1 |
| 5552 | SRSF4 |
| 5553 | LIMCH1 |
| 5554 | ITGB3BP |
| 5555 | FN3K |
| 5556 | CYB561D2 |
| 5557 | CIZ1 |
| 5558 | DSC1 |
| 5559 | PLA2G10 |
| 5560 | RAB11FIP5 |
| 5561 | COQ10B |
| 5562 | OXSR1 |
| 5563 | MPHOSPH10 |
| 5564 | EDN2 |
| 5565 | ZBTB11 |
| 5566 | MGAT5B |
| 5567 | HELZ |
| 5568 | TACSTD2 |
| 5569 | PFKL |
| 5570 | OPN4 |
| 5571 | FGF18 |
| 5572 | MRPL38 |
| 5573 | OPALIN |
| 5574 | FUT3 |
| 5575 | FRY |
| 5576 | WDR75 |
| 5577 | THBS3 |
| 5578 | ROS1 |
| 5579 | RBM26 |
| 5580 | EFHC1 |
| 5581 | ATP6V1B1 |
| 5582 | COA3 |
| 5583 | ABCA13 |
| 5584 | PLEKHG4 |
| 5585 | ARHGEF15 |
| 5586 | CD99 |
| 5587 | ILF3-DT |
| 5588 | GAB2 |
| 5589 | SEH1L |
| 5590 | FOLR2 |
| 5591 | NUDCD1 |
| 5592 | CUX1 |
| 5593 | SYTL2 |
| 5594 | MMP19 |
| 5595 | SPECC1L |
| 5596 | LINC01104 |
| 5597 | DNAAF4-CCPG1 |
| 5598 | NCRUPAR |
| 5599 | SEMA6A |
| 5600 | TNS3 |
| 5601 | ANKHD1-EIF4EBP3 |
| 5602 | ARGLU1 |
| 5603 | TUT1 |
| 5604 | ARMC5 |
| 5605 | STXBP3 |
| 5606 | EXOSC5 |
| 5607 | IMMP2L |
| 5608 | BRD9 |
| 5609 | MYH2 |
| 5610 | CUX2 |
| 5611 | MTMR10 |
| 5612 | PYURF |
| 5613 | TMEM171 |
| 5614 | ABITRAM |
| 5615 | MMP10 |
| 5616 | PTTG1IP |
| 5617 | MTERF2 |
| 5618 | TRIB3 |
| 5619 | SLC10A1 |
| 5620 | APOBEC3C |
| 5621 | DHTKD1 |
| 5622 | DENR |
| 5623 | SNRPF |
| 5624 | INPP5D |
| 5625 | CELF4 |
| 5626 | TMEM199 |
| 5627 | RP9 |
| 5628 | SLAMF6 |
| 5629 | EMC6 |
| 5630 | APLNR |
| 5631 | SLC2A10 |
| 5632 | EIF4EBP3 |
| 5633 | KCNA4 |
| 5634 | NCBP1 |
| 5635 | TLN2 |
| 5636 | GPR68 |
| 5637 | B4GALNT2 |
| 5638 | PBX3 |
| 5639 | ESYT1 |
| 5640 | TAF5 |
| 5641 | NKX6-1 |
| 5642 | DNAJB12 |
| 5643 | SNHG6 |
| 5644 | CA4 |
| 5645 | ROBO2 |
| 5646 | PLCB3 |
| 5647 | ARK2N |
| 5648 | CLIP1 |
| 5649 | RAB6B |
| 5650 | ZBED5 |
| 5651 | LOC130059980 |
| 5652 | LOC130059981 |
| 5653 | KCNJ1 |
| 5654 | POM121 |
| 5655 | CARD11-AS1 |
| 5656 | TMEM86B |
| 5657 | CALML6 |
| 5658 | MLANA |
| 5659 | SLX4IP |
| 5660 | SELENOM |
| 5661 | ATG9A |
| 5662 | PKNOX1 |
| 5663 | ARHGAP21 |
| 5664 | ZNF184 |
| 5665 | NIPSNAP3B |
| 5666 | CYLD-AS1 |
| 5667 | TM2D1 |
| 5668 | TOGARAM1 |
| 5669 | APLP1 |
| 5670 | GTF3C5 |
| 5671 | HASPIN |
| 5672 | FRRS1L |
| 5673 | KCND2 |
| 5674 | EPN2 |
| 5675 | C8orf82 |
| 5676 | ARPC5 |
| 5677 | DLG1 |
| 5678 | GLG1 |
| 5679 | ADAMTS20 |
| 5680 | CCR9 |
| 5681 | FAM53B |
| 5682 | CYP2R1 |
| 5683 | GOLM1 |
| 5684 | TEAD1 |
| 5685 | SMTNL2 |
| 5686 | GATM |
| 5687 | MIR4516 |
| 5688 | PRDM8 |
| 5689 | ITGB7 |
| 5690 | PIGW |
| 5691 | WDR6 |
| 5692 | MED10 |
| 5693 | HSPE1 |
| 5694 | RMND5A |
| 5695 | CNNM4 |
| 5696 | TECR |
| 5697 | SYTL4 |
| 5698 | EXOSC4 |
| 5699 | PRR18 |
| 5700 | GCC1 |
| 5701 | ADGRA2 |
| 5702 | TRPC4 |
| 5703 | MAGED1 |
| 5704 | ANAPC7 |
| 5705 | MAN1A1 |
| 5706 | LRIG3 |
| 5707 | ST3GAL5 |
| 5708 | TYW3 |
| 5709 | PAFAH1B3 |
| 5710 | YRDC |
| 5711 | TMC6 |
| 5712 | HPR |
| 5713 | WDR70 |
| 5714 | PI4K2A |
| 5715 | MT-TY |
| 5716 | VASP |
| 5717 | POLR2I |
| 5718 | KIAA1549 |
| 5719 | RNU6ATAC |
| 5720 | GPR65 |
| 5721 | SERPINB2 |
| 5722 | MGAT2 |
| 5723 | RALY |
| 5724 | CTSH |
| 5725 | CLRN1 |
| 5726 | DRG1 |
| 5727 | MYRIP |
| 5728 | NECTIN2 |
| 5729 | RPUSD4 |
| 5730 | CERS1 |
| 5731 | APTR |
| 5732 | TNNI1 |
| 5733 | HSD3B1 |
| 5734 | ISOC2 |
| 5735 | CAPN12 |
| 5736 | SEPT5-GP1BB |
| 5737 | ZZZ3 |
| 5738 | GLRA1 |
| 5739 | CACYBP |
| 5740 | WDR18 |
| 5741 | SNAPC4 |
| 5742 | TWIST2 |
| 5743 | DDT |
| 5744 | GLMN |
| 5745 | TMEM167A |
| 5746 | RFK |
| 5747 | CEP170 |
| 5748 | TRIP4 |
| 5749 | BOLA2 |
| 5750 | MOCS3 |
| 5751 | RASSF3 |
| 5752 | DUSP16 |
| 5753 | SULF2 |
| 5754 | NPRL3 |
| 5755 | BAAT |
| 5756 | CHST3 |
| 5757 | HAGH |
| 5758 | SCN10A |
| 5759 | KTN1 |
| 5760 | LINC01587 |
| 5761 | MIR4804 |
| 5762 | CHRNB2 |
| 5763 | RND3 |
| 5764 | SLC16A12 |
| 5765 | LGALS9 |
| 5766 | FLAD1 |
| 5767 | LTB |
| 5768 | TNFAIP6 |
| 5769 | MADD |
| 5770 | MTMR2 |
| 5771 | OPRK1 |
| 5772 | NFASC |
| 5773 | SREK1IP1 |
| 5774 | SLC30A2 |
| 5775 | DOCK9 |
| 5776 | TCEAL4 |
| 5777 | RAVER2 |
| 5778 | NINJ1 |
| 5779 | RIT2 |
| 5780 | ZP2 |
| 5781 | TBCB |
| 5782 | BRAP |
| 5783 | USP49 |
| 5784 | ZFPL1 |
| 5785 | LINC00173 |
| 5786 | TMEM86A |
| 5787 | TRL-TAG1-1 |
| 5788 | CCL8 |
| 5789 | GRB14 |
| 5790 | SLC16A7 |
| 5791 | RALGDS |
| 5792 | AXIN2 |
| 5793 | PHF2 |
| 5794 | ALDH1L2 |
| 5795 | DEPTOR |
| 5796 | CDK5RAP1 |
| 5797 | SELENOW |
| 5798 | PRRC2C |
| 5799 | ZNRD2 |
| 5800 | RABAC1 |
| 5801 | AKAP12 |
| 5802 | PRC1 |
| 5803 | SLC8A1 |
| 5804 | CHRNA2 |
| 5805 | SLC22A8 |
| 5806 | FREM1 |
| 5807 | ARHGAP45 |
| 5808 | NPR3 |
| 5809 | EXOSC2 |
| 5810 | SAFB2 |
| 5811 | BLOC1S2 |
| 5812 | AEBP2 |
| 5813 | CLDN16 |
| 5814 | DECR1 |
| 5815 | CPSF4 |
| 5816 | FGR |
| 5817 | TPM4 |
| 5818 | SRPRB |
| 5819 | MED22 |
| 5820 | SYTL1 |
| 5821 | VPS9D1 |
| 5822 | CLMN |
| 5823 | DNAJC12 |
| 5824 | LAMC2 |
| 5825 | TPCN2 |
| 5826 | SPRR3 |
| 5827 | BCAT1 |
| 5828 | SNORA75 |
| 5829 | RAB40A |
| 5830 | FAM199X |
| 5831 | LOC126862464 |
| 5832 | LOC130059979 |
| 5833 | TLDC2 |
| 5834 | SH2D4B |
| 5835 | TARBP1 |
| 5836 | MIR585 |
| 5837 | SLC6A15 |
| 5838 | SRSF5 |
| 5839 | RASSF6 |
| 5840 | WDR11 |
| 5841 | CEP250 |
| 5842 | PCNX3 |
| 5843 | ESAM |
| 5844 | SETD1B |
| 5845 | LINC02153 |
| 5846 | FMNL1-AS1 |
| 5847 | FBXW5 |
| 5848 | MED11 |
| 5849 | RBKS |
| 5850 | ACAA1 |
| 5851 | TRMT61B |
| 5852 | CYP4A11 |
| 5853 | MIR936 |
| 5854 | CPM |
| 5855 | MS4A4A |
| 5856 | KCNH7 |
| 5857 | POLR2M |
| 5858 | WEE2-AS1 |
| 5859 | LOC101928965 |
| 5860 | LOC127898564 |
| 5861 | DNAH14 |
| 5862 | TIMM22 |
| 5863 | ZBTB10 |
| 5864 | SCGN |
| 5865 | MAGOH |
| 5866 | FPR1 |
| 5867 | SDCCAG8 |
| 5868 | MRPL43 |
| 5869 | SLC22A2 |
| 5870 | SUPT4H1 |
| 5871 | SCN2B |
| 5872 | CDK18 |
| 5873 | SPRY4 |
| 5874 | JARID2 |
| 5875 | KIFC3 |
| 5876 | OIP5-AS1 |
| 5877 | PLCB4 |
| 5878 | GIPC2 |
| 5879 | GEMIN5 |
| 5880 | H2BW2 |
| 5881 | MIR545 |
| 5882 | PIP4P1 |
| 5883 | TNFAIP8 |
| 5884 | WIPI1 |
| 5885 | ZNF146 |
| 5886 | HEXIM2 |
| 5887 | ANAPC10 |
| 5888 | TRIT1 |
| 5889 | PDLIM4 |
| 5890 | RALBP1 |
| 5891 | BAZ2B |
| 5892 | TENM1 |
| 5893 | CPNE7 |
| 5894 | ATG12 |
| 5895 | SNCAIP |
| 5896 | CXCL16 |
| 5897 | CASD1 |
| 5898 | PSENEN |
| 5899 | EED |
| 5900 | MIR4521 |
| 5901 | ACP3 |
| 5902 | GGCT |
| 5903 | SRP14 |
| 5904 | LRRC41 |
| 5905 | KIF18B |
| 5906 | MNX1 |
| 5907 | GIP |
| 5908 | VPS28 |
| 5909 | CFAP20 |
| 5910 | LOC109461484 |
| 5911 | AGAP2-AS1 |
| 5912 | WDR33 |
| 5913 | SCAF1 |
| 5914 | LINCMD1 |
| 5915 | GLIS3 |
| 5916 | GIPC1 |
| 5917 | LAMB3 |
| 5918 | UTP6 |
| 5919 | GNMT |
| 5920 | NRM |
| 5921 | ELOVL7 |
| 5922 | RAB3C |
| 5923 | LINC00534 |
| 5924 | FLRT3 |
| 5925 | THEMIS |
| 5926 | VKORC1L1 |
| 5927 | GPM6A |
| 5928 | SCN4B |
| 5929 | SAMD1 |
| 5930 | LARP1 |
| 5931 | MIR490 |
| 5932 | CLASP1 |
| 5933 | FBLN2 |
| 5934 | FNBP1 |
| 5935 | HEPH |
| 5936 | UNK |
| 5937 | CCPG1 |
| 5938 | SHTN1 |
| 5939 | PRMT8 |
| 5940 | RNLS |
| 5941 | C1orf35 |
| 5942 | KCND3 |
| 5943 | BTBD16 |
| 5944 | TXLNA |
| 5945 | ALG14 |
| 5946 | CBX2 |
| 5947 | SOX5 |
| 5948 | BEND3 |
| 5949 | MIR652 |
| 5950 | GLIPR1 |
| 5951 | CYP26B1 |
| 5952 | GUSBP2 |
| 5953 | TBXA2R |
| 5954 | POC1B |
| 5955 | DDX42 |
| 5956 | ASPH |
| 5957 | STC2 |
| 5958 | XAF1 |
| 5959 | ORMDL1 |
| 5960 | FDX1 |
| 5961 | DUSP6 |
| 5962 | PCDH19 |
| 5963 | CSTF2 |
| 5964 | ARL6IP4 |
| 5965 | DLEC1 |
| 5966 | EGOT |
| 5967 | KLF3-AS1 |
| 5968 | VPS72 |
| 5969 | PRPSAP2 |
| 5970 | EIF4B |
| 5971 | ALDH1A3 |
| 5972 | IRX3 |
| 5973 | UQCC1 |
| 5974 | RHOT1 |
| 5975 | SLC27A3 |
| 5976 | AASDHPPT |
| 5977 | MERTK |
| 5978 | FBXL7 |
| 5979 | SMURF2 |
| 5980 | HAS2 |
| 5981 | VMA21 |
| 5982 | SMPD2 |
| 5983 | SAV1 |
| 5984 | OTUD4 |
| 5985 | SPCS3 |
| 5986 | CHTOP |
| 5987 | FAM120AOS |
| 5988 | LINC00365 |
| 5989 | CMA1 |
| 5990 | MED16 |
| 5991 | CLCA1 |
| 5992 | FAXC |
| 5993 | TOMM70 |
| 5994 | SFXN1 |
| 5995 | KIF13A |
| 5996 | POC5 |
| 5997 | MATK |
| 5998 | GET3 |
| 5999 | PEX11A |
| 6000 | NCAPH |
| 6001 | PWP1 |
| 6002 | CHST12 |
| 6003 | CCDC157 |
| 6004 | ECHDC1 |
| 6005 | ATXN3L |
| 6006 | RPF2 |
| 6007 | SMG6 |
| 6008 | HPGD |
| 6009 | ARRB2 |
| 6010 | DUS4L |
| 6011 | SLC7A7 |
| 6012 | EIF3I |
| 6013 | SLC35D3 |
| 6014 | GOPC |
| 6015 | CASC8 |
| 6016 | CMTM5 |
| 6017 | PLD2 |
| 6018 | NUFIP2 |
| 6019 | GNAL |
| 6020 | NFKBIZ |
| 6021 | SEC61G |
| 6022 | USP5 |
| 6023 | CETN3 |
| 6024 | AVEN |
| 6025 | GDF10 |
| 6026 | NRAP |
| 6027 | SMYD3 |
| 6028 | TMEM31 |
| 6029 | TMSB15B |
| 6030 | MORF4L2-AS1 |
| 6031 | TMSB15B-AS1 |
| 6032 | LINC02589 |
| 6033 | TMSB15C |
| 6034 | LOC286437 |
| 6035 | LL0XNC01-250H12.3 |
| 6036 | LOC113845781 |
| 6037 | LOC126863296 |
| 6038 | LOC126863297 |
| 6039 | LOC130068509 |
| 6040 | LOC130068514 |
| 6041 | LOC130068515 |
| 6042 | LOC130068516 |
| 6043 | LOC130068517 |
| 6044 | LOC130068510 |
| 6045 | LOC130068511 |
| 6046 | LOC130068512 |
| 6047 | LOC130068513 |
| 6048 | ATE1 |
| 6049 | PTPRN |
| 6050 | MBNL3 |
| 6051 | MGST1 |
| 6052 | PTPRZ1 |
| 6053 | ZWINT |
| 6054 | SEC14L2 |
| 6055 | TSPYL1 |
| 6056 | RSU1 |
| 6057 | SYF2 |
| 6058 | GYG1 |
| 6059 | GRIK1 |
| 6060 | NPR1 |
| 6061 | VTRNA1-3 |
| 6062 | TENM2 |
| 6063 | AGBL5 |
| 6064 | MAN2A1 |
| 6065 | ALG5 |
| 6066 | CMYA5 |
| 6067 | KCNK2 |
| 6068 | TUBD1 |
| 6069 | LINC00243 |
| 6070 | CEACAM8 |
| 6071 | DFFB |
| 6072 | NXF1 |
| 6073 | HEXA-AS1 |
| 6074 | GAPLINC |
| 6075 | LINC00572 |
| 6076 | LINC00663 |
| 6077 | LINC00689 |
| 6078 | RAMP2-AS1 |
| 6079 | PTCSC2 |
| 6080 | GLIDR |
| 6081 | LINC01494 |
| 6082 | LINC02604 |
| 6083 | LOC100130691 |
| 6084 | DUS4L-BCAP29 |
| 6085 | LINC01277 |
| 6086 | LINC01471 |
| 6087 | LOC285638 |
| 6088 | RPS3AP25 |
| 6089 | LOC105377407 |
| 6090 | LOC114803470 |
| 6091 | LOC129389837 |
| 6092 | ACSBG1 |
| 6093 | ABLIM1 |
| 6094 | TUBGCP3 |
| 6095 | ATXN7L3 |
| 6096 | ZNF260 |
| 6097 | SEPTIN2 |
| 6098 | RNF25 |
| 6099 | MBD6 |
| 6100 | RBMX2 |
| 6101 | KLHL24 |
| 6102 | MIR432 |
| 6103 | TULP3 |
| 6104 | XPO6 |
| 6105 | PLAGL2 |
| 6106 | MSMO1 |
| 6107 | ATP10A |
| 6108 | DEF6 |
| 6109 | CCL24 |
| 6110 | FKBP3 |
| 6111 | NFE2L1 |
| 6112 | UNC5B |
| 6113 | PYCARD |
| 6114 | IFITM1 |
| 6115 | ZNF12 |
| 6116 | BNC1 |
| 6117 | CIR1 |
| 6118 | ATP5MC1 |
| 6119 | PPL |
| 6120 | NAP1L4 |
| 6121 | EEA1 |
| 6122 | MRPL28 |
| 6123 | CCDC61 |
| 6124 | NCAN |
| 6125 | DACH1 |
| 6126 | UBE4A |
| 6127 | EN2 |
| 6128 | PTN |
| 6129 | PAX9 |
| 6130 | VTI1B |
| 6131 | ATP5MF |
| 6132 | ADM2 |
| 6133 | CA7 |
| 6134 | HLA-H |
| 6135 | IRF2 |
| 6136 | KCNQ5 |
| 6137 | IL1F10 |
| 6138 | ANKRD30A |
| 6139 | IGFBP5 |
| 6140 | PLLP |
| 6141 | NUDT6 |
| 6142 | SRM |
| 6143 | COG3 |
| 6144 | POGLUT1 |
| 6145 | RBM27 |
| 6146 | CLIC5 |
| 6147 | NBR2 |
| 6148 | SSH2 |
| 6149 | PCF11 |
| 6150 | PDGFC |
| 6151 | DBI |
| 6152 | TFF1 |
| 6153 | PINK1-AS |
| 6154 | IQCE |
| 6155 | DCP2 |
| 6156 | UPP2 |
| 6157 | BBOF1 |
| 6158 | ZNF337 |
| 6159 | PTER |
| 6160 | FAR2 |
| 6161 | UBXN4 |
| 6162 | DLGAP2 |
| 6163 | EPB42 |
| 6164 | NAB2 |
| 6165 | GNG12 |
| 6166 | CCL13 |
| 6167 | NGB |
| 6168 | NR2C1 |
| 6169 | KLHL22 |
| 6170 | ASCL1 |
| 6171 | TRAF4 |
| 6172 | DNAH17 |
| 6173 | ERI1 |
| 6174 | SWAP70 |
| 6175 | AFTPH |
| 6176 | TRMT5 |
| 6177 | ASB2 |
| 6178 | RD3 |
| 6179 | TUBGCP4 |
| 6180 | RRP1 |
| 6181 | DXO |
| 6182 | LRP4 |
| 6183 | CSNK1G1 |
| 6184 | ZFP14 |
| 6185 | INSIG1 |
| 6186 | CYB5B |
| 6187 | POLR1D |
| 6188 | NDUFC2-KCTD14 |
| 6189 | LINC01419 |
| 6190 | DDAH1 |
| 6191 | RPS21 |
| 6192 | PWRN1 |
| 6193 | HOMER3 |
| 6194 | NEDD1 |
| 6195 | SOCS2-AS1 |
| 6196 | SELENOV |
| 6197 | MIA-RAB4B |
| 6198 | PCDH11X |
| 6199 | C8A |
| 6200 | PSD |
| 6201 | BACE2 |
| 6202 | SRPK2 |
| 6203 | RICTOR |
| 6204 | CDK20 |
| 6205 | NPM3 |
| 6206 | JMJD6 |
| 6207 | QTRT1 |
| 6208 | SNHG14 |
| 6209 | PHKA2 |
| 6210 | NDUFV3 |
| 6211 | MORC1 |
| 6212 | ZFYVE27 |
| 6213 | ADAT2 |
| 6214 | CPT1B |
| 6215 | RORC |
| 6216 | REEP3 |
| 6217 | NLRC5 |
| 6218 | CADM2 |
| 6219 | ZNF407 |
| 6220 | STRN3 |
| 6221 | EFNB3 |
| 6222 | EFCAB15P |
| 6223 | KDM1B |
| 6224 | TRO |
| 6225 | EDF1 |
| 6226 | PLEKHA5 |
| 6227 | PLXDC2 |
| 6228 | EIF3D |
| 6229 | OCM |
| 6230 | VPS16 |
| 6231 | ARG2 |
| 6232 | CLPP |
| 6233 | FKBP6 |
| 6234 | QRFP |
| 6235 | ZFPM2 |
| 6236 | MIER1 |
| 6237 | NLRX1 |
| 6238 | MUCL3 |
| 6239 | ZNF205 |
| 6240 | RTF2 |
| 6241 | SPTBN5 |
| 6242 | CTNNBIP1 |
| 6243 | GOSR1 |
| 6244 | SCARF2 |
| 6245 | THOC7 |
| 6246 | DNM3OS |
| 6247 | KCNJ12 |
| 6248 | FSTL1 |
| 6249 | USP9Y |
| 6250 | RNF43 |
| 6251 | LTBP4 |
| 6252 | VPS25 |
| 6253 | CANT1 |
| 6254 | RRP9 |
| 6255 | CDO1 |
| 6256 | LRRC8B |
| 6257 | PAPPA |
| 6258 | SLC17A1 |
| 6259 | DHRS7B |
| 6260 | LINC00473 |
| 6261 | LOC126862463 |
| 6262 | LOC129390823 |
| 6263 | LOC129390824 |
| 6264 | LOC130059975 |
| 6265 | LOC130059976 |
| 6266 | LOC130059977 |
| 6267 | LOC130059978 |
| 6268 | LOC130059982 |
| 6269 | LOC130059983 |
| 6270 | LOC130059984 |
| 6271 | LOC130059985 |
| 6272 | LOC130059986 |
| 6273 | PRKAR2B |
| 6274 | SIX1 |
| 6275 | ACVR2B |
| 6276 | PXMP4 |
| 6277 | DCAF4 |
| 6278 | MED30 |
| 6279 | DLX4 |
| 6280 | KANK4 |
| 6281 | LIMK2 |
| 6282 | FIGN |
| 6283 | CA5A |
| 6284 | PENK |
| 6285 | CHCHD2 |
| 6286 | IFT88 |
| 6287 | SLC39A6 |
| 6288 | NAGK |
| 6289 | LOC129930446 |
| 6290 | SLC12A7 |
| 6291 | STX3 |
| 6292 | EHD1 |
| 6293 | PLEK2 |
| 6294 | INHBB |
| 6295 | NMT2 |
| 6296 | CARD16 |
| 6297 | NT5DC2 |
| 6298 | PDE11A |
| 6299 | PEX11G |
| 6300 | ZDHHC20 |
| 6301 | GGPS1 |
| 6302 | FGD4 |
| 6303 | COMMD1 |
| 6304 | SYT7 |
| 6305 | LTV1 |
| 6306 | KIF2C |
| 6307 | ESAM-AS1 |
| 6308 | CAAP1 |
| 6309 | DDO |
| 6310 | BMP15 |
| 6311 | SSBP4 |
| 6312 | P2RX4 |
| 6313 | SDHAF2 |
| 6314 | TMEM240 |
| 6315 | HLA-S |
| 6316 | ITGA8 |
| 6317 | TUT7 |
| 6318 | MTMR1 |
| 6319 | SCAPER |
| 6320 | CTAG2 |
| 6321 | XPR1 |
| 6322 | TRV-AAC1-4 |
| 6323 | MIR615 |
| 6324 | UBE2E1 |
| 6325 | KDELR3 |
| 6326 | SRP9 |
| 6327 | KSR1 |
| 6328 | NPDC1 |
| 6329 | SERPINB5 |
| 6330 | BICRA |
| 6331 | FOXI3 |
| 6332 | NHLRC1 |
| 6333 | ABHD4 |
| 6334 | NPAP1 |
| 6335 | GTF2F2 |
| 6336 | HTR2B |
| 6337 | HSD3B2 |
| 6338 | ASB11 |
| 6339 | YKT6 |
| 6340 | TRANK1 |
| 6341 | SMOX |
| 6342 | NKAPL |
| 6343 | GLRA2 |
| 6344 | FGL2 |
| 6345 | EIF4H |
| 6346 | LEFTY1 |
| 6347 | NOMO1 |
| 6348 | USH1G |
| 6349 | SPAG5 |
| 6350 | POM121C |
| 6351 | TUSC3 |
| 6352 | ADCY1 |
| 6353 | ARMC7 |
| 6354 | KIAA1614 |
| 6355 | GDI1 |
| 6356 | TMCC2 |
| 6357 | OLFM1 |
| 6358 | TMEM184B |
| 6359 | TBC1D10A |
| 6360 | RCVRN |
| 6361 | PSMC3IP |
| 6362 | FZD7 |
| 6363 | GADL1 |
| 6364 | PAM16 |
| 6365 | CCR8 |
| 6366 | COMMD5 |
| 6367 | EXOC4 |
| 6368 | AMY2B |
| 6369 | HAP1 |
| 6370 | NTN4 |
| 6371 | NIN |
| 6372 | DENND2B |
| 6373 | MIR133A2 |
| 6374 | IL13RA2 |
| 6375 | HSD17B12 |
| 6376 | ALAS1 |
| 6377 | NOSIP |
| 6378 | POU3F2 |
| 6379 | GAS7 |
| 6380 | ZNF593 |
| 6381 | PKN2 |
| 6382 | EXOSC6 |
| 6383 | FAM234A |
| 6384 | MAP1A |
| 6385 | MIR663A |
| 6386 | MASP1 |
| 6387 | MIR4286 |
| 6388 | SLC25A26 |
| 6389 | MIR659 |
| 6390 | CDC42EP1 |
| 6391 | DAP |
| 6392 | GYPE |
| 6393 | INTS1 |
| 6394 | FAM20A |
| 6395 | CEP95 |
| 6396 | SNAP47 |
| 6397 | MROCKI |
| 6398 | TP53TG1 |
| 6399 | PLXNB2 |
| 6400 | PHF14 |
| 6401 | CNOT9 |
| 6402 | NKX2-8 |
| 6403 | SMYD2 |
| 6404 | BOLA1 |
| 6405 | DIAPH3 |
| 6406 | ACOT1 |
| 6407 | SIX4 |
| 6408 | TMPPE |
| 6409 | BCKDK |
| 6410 | ILRUN |
| 6411 | MAST2 |
| 6412 | SNORA64 |
| 6413 | COTL1 |
| 6414 | RAB11FIP1 |
| 6415 | COX17 |
| 6416 | CCN6 |
| 6417 | CYP39A1 |
| 6418 | CA12 |
| 6419 | PRXL2A |
| 6420 | CORO1C |
| 6421 | RNGTT |
| 6422 | PROX1 |
| 6423 | GALR2 |
| 6424 | AFF3 |
| 6425 | CD6 |
| 6426 | BUD23 |
| 6427 | MCEE |
| 6428 | PDE1C |
| 6429 | PFDN5 |
| 6430 | TRMT2A |
| 6431 | PARP8 |
| 6432 | ICAM5 |
| 6433 | WWTR1 |
| 6434 | HVCN1 |
| 6435 | FZD1 |
| 6436 | PRPS1L1 |
| 6437 | ARL6IP6 |
| 6438 | DCPS |
| 6439 | FAM83H |
| 6440 | TEP1 |
| 6441 | POFUT1 |
| 6442 | RENBP |
| 6443 | COX8A |
| 6444 | FBXO17 |
| 6445 | LPIN3 |
| 6446 | OFCC1 |
| 6447 | DSCC1 |
| 6448 | PHKB |
| 6449 | TUT4 |
| 6450 | INPP5B |
| 6451 | SLC24A3 |
| 6452 | NHERF2 |
| 6453 | CEP72 |
| 6454 | C1orf94 |
| 6455 | CD83 |
| 6456 | SLC25A2 |
| 6457 | LMOD3 |
| 6458 | AQP8 |
| 6459 | CFAP44 |
| 6460 | PRCD |
| 6461 | BORCS6 |
| 6462 | CATSPERG |
| 6463 | COX11 |
| 6464 | TXNRD3 |
| 6465 | GGA3 |
| 6466 | PRDM7 |
| 6467 | MED6 |
| 6468 | PRSS16 |
| 6469 | SLC12A4 |
| 6470 | EPM2A |
| 6471 | POLR1B |
| 6472 | RAB28 |
| 6473 | STX16 |
| 6474 | PPP6R1 |
| 6475 | GRM4 |
| 6476 | RAB11FIP2 |
| 6477 | SPAG9 |
| 6478 | SF3B6 |
| 6479 | CYP26C1 |
| 6480 | PRG4 |
| 6481 | MEPCE |
| 6482 | FOXN1 |
| 6483 | HAUS5 |
| 6484 | IST1 |
| 6485 | OXER1 |
| 6486 | CCT6B |
| 6487 | ISY1 |
| 6488 | WDPCP |
| 6489 | ZNF707 |
| 6490 | MEAK7 |
| 6491 | LPCAT1 |
| 6492 | P4HTM |
| 6493 | COL14A1 |
| 6494 | MID2 |
| 6495 | TGFBR3 |
| 6496 | EGFL7 |
| 6497 | ACY3 |
| 6498 | ZC3H3 |
| 6499 | BAIAP2 |
| 6500 | CHCHD4 |
| 6501 | ADGRE2 |
| 6502 | STARD7 |
| 6503 | CLDND1 |
| 6504 | ECHDC2 |
| 6505 | FNDC10 |
| 6506 | TRA |
| 6507 | TGOLN2 |
| 6508 | TACR2 |
| 6509 | FUT8 |
| 6510 | GNG2 |
| 6511 | ARPC5L |
| 6512 | MYO9A |
| 6513 | CCDC85A |
| 6514 | GOLGA5 |
| 6515 | TIMP4 |
| 6516 | MIR4485 |
| 6517 | TRIM9 |
| 6518 | TNNT3 |
| 6519 | ELAPOR2 |
| 6520 | MN1 |
| 6521 | C1orf226 |
| 6522 | TRIM17 |
| 6523 | ACSM1 |
| 6524 | TMC8 |
| 6525 | CEP97 |
| 6526 | FOCAD |
| 6527 | GABPA |
| 6528 | LRRC8D |
| 6529 | PRDM15 |
| 6530 | MTMR12 |
| 6531 | VEGFD |
| 6532 | MAPRE3 |
| 6533 | TRMT11 |
| 6534 | PWAR6 |
| 6535 | CLINT1 |
| 6536 | KCTD3 |
| 6537 | SNORD24 |
| 6538 | DDX28 |
| 6539 | SFT2D2 |
| 6540 | ZNF597 |
| 6541 | RPS6KA1 |
| 6542 | TNFAIP1 |
| 6543 | SLC25A37 |
| 6544 | PCSK2 |
| 6545 | SNAPC3 |
| 6546 | PCMTD2 |
| 6547 | ZNF566 |
| 6548 | ZNF628 |
| 6549 | LHX1 |
| 6550 | LRRC8C |
| 6551 | SH3D19 |
| 6552 | KLK11 |
| 6553 | HLA-F-AS1 |
| 6554 | DSG4 |
| 6555 | LIX1 |
| 6556 | ZNF7 |
| 6557 | UTP20 |
| 6558 | GLUD2 |
| 6559 | KATNAL2 |
| 6560 | SPPL2A |
| 6561 | LINC-PINT |
| 6562 | XPNPEP3 |
| 6563 | COPZ1 |
| 6564 | MPHOSPH9 |
| 6565 | B3GALT4 |
| 6566 | PPT2-EGFL8 |
| 6567 | ELMO1 |
| 6568 | ADAMTS7 |
| 6569 | LILRA2 |
| 6570 | PCSK1N |
| 6571 | MED21 |
| 6572 | PLXND1 |
| 6573 | ABTB2 |
| 6574 | DNAAF6 |
| 6575 | SSR3 |
| 6576 | MAF1 |
| 6577 | BBIP1 |
| 6578 | CLEC4D |
| 6579 | AP5B1 |
| 6580 | ELAVL2 |
| 6581 | AKIP1 |
| 6582 | NVL |
| 6583 | GNPDA2 |
| 6584 | CA8 |
| 6585 | ZC3H14 |
| 6586 | NDRG4 |
| 6587 | SLC25A16 |
| 6588 | TRIM54 |
| 6589 | SLC44A2 |
| 6590 | WASHC5-AS1 |
| 6591 | ABCD1P1 |
| 6592 | GDF3 |
| 6593 | TMEM116 |
| 6594 | NKTR |
| 6595 | LOC129992929 |
| 6596 | DPH5 |
| 6597 | TBATA |
| 6598 | AMY2A |
| 6599 | ACBD3 |
| 6600 | ZRSR2 |
| 6601 | KCNN3 |
| 6602 | AGO1 |
| 6603 | QRICH1 |
| 6604 | GABRA4 |
| 6605 | MOS |
| 6606 | ETFRF1 |
| 6607 | TMX1 |
| 6608 | CPNE3 |
| 6609 | PCDH7 |
| 6610 | BSN |
| 6611 | GPR89A |
| 6612 | KNSTRN |
| 6613 | SEMA3E |
| 6614 | APOL2 |
| 6615 | SARNP |
| 6616 | SH3BP5 |
| 6617 | DGKB |
| 6618 | RLF |
| 6619 | MTERF3 |
| 6620 | NCBP2 |
| 6621 | SAC3D1 |
| 6622 | SAR1B |
| 6623 | MAK16 |
| 6624 | UNC5A |
| 6625 | PRSS56 |
| 6626 | LTK |
| 6627 | SQOR |
| 6628 | PLEKHH1 |
| 6629 | ZNF395 |
| 6630 | LIMS2 |
| 6631 | SLC12A9 |
| 6632 | R3HCC1L |
| 6633 | PDCD7 |
| 6634 | OSGEPL1 |
| 6635 | TSPAN9 |
| 6636 | EMP1 |
| 6637 | SIPA1 |
| 6638 | PPAN |
| 6639 | SLC48A1 |
| 6640 | MIR218-1 |
| 6641 | TAF1C |
| 6642 | EN1 |
| 6643 | CLDN7 |
| 6644 | FAM20C |
| 6645 | ATP5MC3 |
| 6646 | PACSIN3 |
| 6647 | SNX5 |
| 6648 | WDR77 |
| 6649 | TARBP2 |
| 6650 | LRRFIP2 |
| 6651 | L3HYPDH |
| 6652 | KLHL21 |
| 6653 | APPBP2 |
| 6654 | ARTN |
| 6655 | HOXA13 |
| 6656 | KCNAB1 |
| 6657 | GTF2A1 |
| 6658 | RCAN1 |
| 6659 | ZACN |
| 6660 | PAAF1 |
| 6661 | AMD1 |
| 6662 | GTF2IRD2 |
| 6663 | GNPTG |
| 6664 | LY9 |
| 6665 | PPP2R3A |
| 6666 | EXOC3L2 |
| 6667 | SGK2 |
| 6668 | GCM2 |
| 6669 | SLC1A6 |
| 6670 | GNAI2 |
| 6671 | GALNT6 |
| 6672 | SOX18 |
| 6673 | SNURF |
| 6674 | MIR6891 |
| 6675 | ARFGAP1 |
| 6676 | SNAPIN |
| 6677 | MCF2L |
| 6678 | SEC61B |
| 6679 | ANGPTL8 |
| 6680 | PEMT |
| 6681 | MLST8 |
| 6682 | FAM133B |
| 6683 | SAMD12 |
| 6684 | HAPLN2 |
| 6685 | HMGN4 |
| 6686 | EPB41L1 |
| 6687 | NCAM2 |
| 6688 | SMC1B |
| 6689 | ELOF1 |
| 6690 | BORCS5 |
| 6691 | PNOC |
| 6692 | S100A2 |
| 6693 | HECTD1 |
| 6694 | PROK2 |
| 6695 | BMPR1B |
| 6696 | RABGGTA |
| 6697 | SLCO1A2 |
| 6698 | NMB |
| 6699 | TNFRSF21 |
| 6700 | ZAN |
| 6701 | PRRC2B |
| 6702 | MAB21L2 |
| 6703 | ZSWIM2 |
| 6704 | MIR22HG |
| 6705 | PIH1D2 |
| 6706 | SLC38A10 |
| 6707 | LOC109461479 |
| 6708 | METTL27 |
| 6709 | TMEM270 |
| 6710 | HSD17B7 |
| 6711 | HCG18 |
| 6712 | TOM1 |
| 6713 | PDLIM2 |
| 6714 | RPL36A |
| 6715 | ATP6V0E2 |
| 6716 | MIR301B |
| 6717 | CNPY3 |
| 6718 | STIM2 |
| 6719 | RAB3IP |
| 6720 | LOC130056998 |
| 6721 | GBA3 |
| 6722 | PSMG4 |
| 6723 | INTS11 |
| 6724 | CHMP5 |
| 6725 | ATCAY |
| 6726 | HCG14 |
| 6727 | HCG15 |
| 6728 | EIF3M |
| 6729 | SLC66A1 |
| 6730 | SLC6A19 |
| 6731 | PEA15 |
| 6732 | GRPR |
| 6733 | MS4A6A |
| 6734 | WDR46 |
| 6735 | P2RY1 |
| 6736 | PTBP2 |
| 6737 | CEP131 |
| 6738 | FAM83C |
| 6739 | SCIN |
| 6740 | TIMM21 |
| 6741 | GPR161 |
| 6742 | HSD17B13 |
| 6743 | AHCYL2 |
| 6744 | SNTG1 |
| 6745 | REEP4 |
| 6746 | ZNF346 |
| 6747 | MRPS23 |
| 6748 | ZP3 |
| 6749 | CNTFR |
| 6750 | RPL37 |
| 6751 | GZMH |
| 6752 | GRID2 |
| 6753 | KIR2DS2 |
| 6754 | S100A3 |
| 6755 | MIF4GD |
| 6756 | PARVB |
| 6757 | BRK1 |
| 6758 | ARAF |
| 6759 | USP26 |
| 6760 | RNR1 |
| 6761 | WASF2 |
| 6762 | CNTNAP5 |
| 6763 | BLOC1S1 |
| 6764 | ATP1B2 |
| 6765 | MIR3144 |
| 6766 | DBNL |
| 6767 | EDA |
| 6768 | SSTR2 |
| 6769 | POF1B |
| 6770 | EIF2A |
| 6771 | GOLIM4 |
| 6772 | KIF17 |
| 6773 | VTA1 |
| 6774 | SRFBP1 |
| 6775 | SGF29 |
| 6776 | NAPRT |
| 6777 | PPP1R1B |
| 6778 | SLC13A2 |
| 6779 | CSRNP3 |
| 6780 | SCAP |
| 6781 | LMLN |
| 6782 | AP1S2 |
| 6783 | SENP7 |
| 6784 | MIR1224 |
| 6785 | ADH4 |
| 6786 | GPR37 |
| 6787 | MIR506 |
| 6788 | NSUN5 |
| 6789 | UBR3 |
| 6790 | PLUT |
| 6791 | HCG20 |
| 6792 | MICAL1 |
| 6793 | SLTM |
| 6794 | USP29 |
| 6795 | ANO3 |
| 6796 | PQBP1 |
| 6797 | OR8U3 |
| 6798 | MRC2 |
| 6799 | CACTIN |
| 6800 | RTCA |
| 6801 | GDF11 |
| 6802 | LOC112272578 |
| 6803 | GUF1 |
| 6804 | SYTL3 |
| 6805 | SIX5 |
| 6806 | ANKRD36B |
| 6807 | PIGM |
| 6808 | HTT-AS |
| 6809 | PPFIA2 |
| 6810 | DIRAS2 |
| 6811 | MDM1 |
| 6812 | ATL2 |
| 6813 | LENG1 |
| 6814 | USF2 |
| 6815 | LOC129936434 |
| 6816 | CLDN6 |
| 6817 | TMEM123 |
| 6818 | MAB21L1 |
| 6819 | CWF19L2 |
| 6820 | LRRC14 |
| 6821 | KANSL2 |
| 6822 | NSUN6 |
| 6823 | KCTD2 |
| 6824 | SPATA2L |
| 6825 | EID2B |
| 6826 | TRAM1 |
| 6827 | PANX2 |
| 6828 | EHD3 |
| 6829 | TENM3 |
| 6830 | KATNAL1 |
| 6831 | MUC4 |
| 6832 | TM9SF3 |
| 6833 | DEPDC7 |
| 6834 | ERGIC3 |
| 6835 | UBA6 |
| 6836 | KYAT3 |
| 6837 | ABI1 |
| 6838 | RNA18SN1 |
| 6839 | CFHR2 |
| 6840 | FRMD7 |
| 6841 | CABLES1 |
| 6842 | C18orf21 |
| 6843 | NRGN |
| 6844 | MIR147B |
| 6845 | CA10 |
| 6846 | EFHD2 |
| 6847 | SLC17A3 |
| 6848 | DAGLB |
| 6849 | FNBP4 |
| 6850 | CSTF3 |
| 6851 | PLEKHG3 |
| 6852 | MRPS21 |
| 6853 | TM7SF2 |
| 6854 | POPDC3 |
| 6855 | CNIH3 |
| 6856 | MYO10 |
| 6857 | FAM118A |
| 6858 | SEMA3D |
| 6859 | DCDC1 |
| 6860 | MIR3613 |
| 6861 | AFDN |
| 6862 | DOCK10 |
| 6863 | CACNB4 |
| 6864 | DDX60L |
| 6865 | COA7 |
| 6866 | GAS6 |
| 6867 | TSTD1 |
| 6868 | SMAD6 |
| 6869 | MFSD12 |
| 6870 | MAN2A2 |
| 6871 | SEMA3B |
| 6872 | MTARC2 |
| 6873 | NEU2 |
| 6874 | GGT7 |
| 6875 | COA5 |
| 6876 | ZNF461 |
| 6877 | piR-38252 |
| 6878 | METTL21A |
| 6879 | NTSR1 |
| 6880 | SH2D2A |
| 6881 | CA6 |
| 6882 | BPHL |
| 6883 | MBTD1 |
| 6884 | LGALS7B |
| 6885 | ANKRD49 |
| 6886 | LETM2 |
| 6887 | BRWD3 |
| 6888 | CBLN4 |
| 6889 | CROCC |
| 6890 | SSTR5 |
| 6891 | EIPR1 |
| 6892 | MIR1182 |
| 6893 | KIF3A |
| 6894 | MRPL11 |
| 6895 | NDUFA5 |
| 6896 | CNTN3 |
| 6897 | PAM |
| 6898 | DSCAML1 |
| 6899 | GPC6 |
| 6900 | RNR4 |
| 6901 | KCNJ3 |
| 6902 | SETD3 |
| 6903 | KIR2DL2 |
| 6904 | STXBP4 |
| 6905 | ZNF16 |
| 6906 | EMILIN3 |
| 6907 | UBE3D |
| 6908 | SPATS1 |
| 6909 | KERA |
| 6910 | FANK1 |
| 6911 | SHANK1 |
| 6912 | PLEKHG4B |
| 6913 | GLIS2 |
| 6914 | DAZ1 |
| 6915 | RNF125 |
| 6916 | NUB1 |
| 6917 | PRKACG |
| 6918 | FAM50A |
| 6919 | SLC39A10 |
| 6920 | SEMA7A |
| 6921 | LIMS1 |
| 6922 | MKRN3 |
| 6923 | SLC22A6 |
| 6924 | WDR7 |
| 6925 | MRPL57 |
| 6926 | ASNSD1 |
| 6927 | EIF3CL |
| 6928 | CLTA |
| 6929 | STX1B |
| 6930 | NIPA2 |
| 6931 | EML4 |
| 6932 | NCAPG2 |
| 6933 | NADSYN1 |
| 6934 | NID2 |
| 6935 | PXN |
| 6936 | PLSCR3 |
| 6937 | KCNJ16 |
| 6938 | SQLE |
| 6939 | VNN3P |
| 6940 | ASIC2 |
| 6941 | ICAM2 |
| 6942 | COX7A2L |
| 6943 | HOXB-AS3 |
| 6944 | MYORG |
| 6945 | ATXN7L2 |
| 6946 | SPECC1 |
| 6947 | AIF1L |
| 6948 | PRIMA1 |
| 6949 | LPCAT2 |
| 6950 | LMO4 |
| 6951 | SGCZ |
| 6952 | SDK2 |
| 6953 | LMF2 |
| 6954 | EPB41L4B |
| 6955 | FAM171A1 |
| 6956 | C16orf74 |
| 6957 | GARNL3 |
| 6958 | SLC7A8 |
| 6959 | SNORD64 |
| 6960 | SNORD109B |
| 6961 | SNORD107 |
| 6962 | LINC02250 |
| 6963 | EOLA1-DT |
| 6964 | LINC00621 |
| 6965 | MAGEA8-AS1 |
| 6966 | LOC106050103 |
| 6967 | MAPKAP1 |
| 6968 | ATP8B4 |
| 6969 | GRK6 |
| 6970 | COLQ |
| 6971 | PPP6R2 |
| 6972 | CA5B |
| 6973 | SYCP1 |
| 6974 | NECAP2 |
| 6975 | MARK2 |
| 6976 | COX6A2 |
| 6977 | PLCL1 |
| 6978 | NUDT10 |
| 6979 | METAP2 |
| 6980 | PLAC1 |
| 6981 | PSD2 |
| 6982 | TACR3 |
| 6983 | KHDC4 |
| 6984 | HS3ST3B1 |
| 6985 | ZBTB17 |
| 6986 | DIO3 |
| 6987 | KIF27 |
| 6988 | ZNF595 |
| 6989 | CROT |
| 6990 | SLC22A18AS |
| 6991 | AHRR |
| 6992 | ADRA1D |
| 6993 | QRICH2 |
| 6994 | GAS8-AS1 |
| 6995 | RBM48 |
| 6996 | CNTN5 |
| 6997 | KRT76 |
| 6998 | KLF13 |
| 6999 | CTNNAL1 |
| 7000 | TMUB1 |
| 7001 | ATG2A |
| 7002 | KRT31 |
| 7003 | GATD3 |
| 7004 | MS |
| 7005 | LLGL2 |
| 7006 | LAPTM5 |
| 7007 | GPATCH2 |
| 7008 | DAAM1 |
| 7009 | AGBL2 |
| 7010 | SLC25A27 |
| 7011 | PLEKHF1 |
| 7012 | CCDC14 |
| 7013 | ATP8B2 |
| 7014 | AVPR1A |
| 7015 | ANAPC4 |
| 7016 | GZMK |
| 7017 | TRP-TGG3-1 |
| 7018 | DBET |
| 7019 | YIF1A |
| 7020 | PDGFRL |
| 7021 | CDH4 |
| 7022 | ZBTB48 |
| 7023 | EFNA1 |
| 7024 | MTFR1L |
| 7025 | CEP89 |
| 7026 | FNTA |
| 7027 | HDHD2 |
| 7028 | UQCRQ |
| 7029 | LINC02907 |
| 7030 | RBM15B |
| 7031 | CCDC12 |
| 7032 | CNOT11 |
| 7033 | NHLH1 |
| 7034 | RTP2 |
| 7035 | AP3S1 |
| 7036 | FAM136A |
| 7037 | TXK |
| 7038 | ANKRD2 |
| 7039 | CHGB |
| 7040 | RASSF5 |
| 7041 | DUSP15 |
| 7042 | LINC01132 |
| 7043 | ATF5 |
| 7044 | SLC35F2 |
| 7045 | ZNF275 |
| 7046 | CLTB |
| 7047 | TMEM245 |
| 7048 | ZNF2 |
| 7049 | H4C7 |
| 7050 | LINC01599 |
| 7051 | LINC01588 |
| 7052 | HS3ST1 |
| 7053 | MIR4315-1 |
| 7054 | GYS2 |
| 7055 | LGMNP1 |
| 7056 | TRDN |
| 7057 | ATP6V0A1 |
| 7058 | STAP1 |
| 7059 | LURAP1 |
| 7060 | MXRA5 |
| 7061 | LOC108660406 |
| 7062 | SULT4A1 |
| 7063 | TMEM88 |
| 7064 | C1orf210 |
| 7065 | NECTIN1 |
| 7066 | KCNIP3 |
| 7067 | ARHGEF9 |
| 7068 | CCDC170 |
| 7069 | CCM2 |
| 7070 | RTF1 |
| 7071 | ATRN |
| 7072 | SNORD50A |
| 7073 | RBMY1A1 |
| 7074 | ECPAS |
| 7075 | CD200 |
| 7076 | DZIP1L |
| 7077 | BCAR1 |
| 7078 | KIAA0232 |
| 7079 | AMY1B |
| 7080 | AFG1L |
| 7081 | TMEM160 |
| 7082 | SHISA6 |
| 7083 | GPATCH3 |
| 7084 | GIT1 |
| 7085 | SEMA3F |
| 7086 | NBPF14 |
| 7087 | PTP4A1 |
| 7088 | VSNL1 |
| 7089 | ANGEL2 |
| 7090 | ONECUT2 |
| 7091 | VPS9D1-AS1 |
| 7092 | CHI3L2 |
| 7093 | KCNJ8 |
| 7094 | SPP2 |
| 7095 | ERG28 |
| 7096 | TSHB |
| 7097 | SNX15 |
| 7098 | KISS1 |
| 7099 | PKD1P6 |
| 7100 | INTS2 |
| 7101 | DLX6 |
| 7102 | TAS2R38 |
| 7103 | RRP7A |
| 7104 | GYPB |
| 7105 | TSPOAP1 |
| 7106 | MROH6 |
| 7107 | DMWD |
| 7108 | SNORD95 |
| 7109 | CNTRL |
| 7110 | NOB1 |
| 7111 | LIPF |
| 7112 | NPTX1 |
| 7113 | IL20 |
| 7114 | MIR3065 |
| 7115 | MIR218-2 |
| 7116 | MTMR8 |
| 7117 | HIVEP3 |
| 7118 | HIF1AN |
| 7119 | UQCRH |
| 7120 | LOC126806039 |
| 7121 | FABP7 |
| 7122 | VAMP1 |
| 7123 | SIK2 |
| 7124 | FAM180B |
| 7125 | ZFP82 |
| 7126 | CPEB2 |
| 7127 | FYTTD1 |
| 7128 | TOX3 |
| 7129 | FAM193A |
| 7130 | MIR377 |
| 7131 | AADAT |
| 7132 | TST |
| 7133 | RASGRP4 |
| 7134 | PPP1R9B |
| 7135 | LOC130060223 |
| 7136 | RBP1 |
| 7137 | PPP4R4 |
| 7138 | MELTF |
| 7139 | PHLDB2 |
| 7140 | TMEM11 |
| 7141 | ZSWIM8 |
| 7142 | SLC17A6 |
| 7143 | EIF3J |
| 7144 | GNAS-AS1 |
| 7145 | VWA5B2 |
| 7146 | MIR1246 |
| 7147 | ACTL7A |
| 7148 | CETN1 |
| 7149 | SYT11 |
| 7150 | LOC125024479 |
| 7151 | LOC125024480 |
| 7152 | LOC129390630 |
| 7153 | LOC130055566 |
| 7154 | LOC130055567 |
| 7155 | LOC130055568 |
| 7156 | LOC130055569 |
| 7157 | LOC130055570 |
| 7158 | LOC130055571 |
| 7159 | LOC130055572 |
| 7160 | LOC130055573 |
| 7161 | LOC130055574 |
| 7162 | LOC130055575 |
| 7163 | LOC130055576 |
| 7164 | LOC130055577 |
| 7165 | LOC130055578 |
| 7166 | LOC130055579 |
| 7167 | LOC130055580 |
| 7168 | LOC130055581 |
| 7169 | LOC130055582 |
| 7170 | LOC130055583 |
| 7171 | LOC130055584 |
| 7172 | LOC130055585 |
| 7173 | CYP4F3 |
| 7174 | RBMS1 |
| 7175 | MIR300 |
| 7176 | RBFA |
| 7177 | ARL2 |
| 7178 | CCNY |
| 7179 | NAV1 |
| 7180 | ATP8B3 |
| 7181 | CHMP2A |
| 7182 | GIT2 |
| 7183 | TWF1 |
| 7184 | SV2A |
| 7185 | INAVA |
| 7186 | TMC2 |
| 7187 | PHLDB3 |
| 7188 | SOD1-DT |
| 7189 | CWC15 |
| 7190 | TMTC2 |
| 7191 | RUBCN |
| 7192 | ANKRD44 |
| 7193 | HEYL |
| 7194 | IGSF1 |
| 7195 | MRPS33 |
| 7196 | TLE5 |
| 7197 | MYMK |
| 7198 | STX17 |
| 7199 | NPFF |
| 7200 | ARHGAP30 |
| 7201 | ERVFRD-1 |
| 7202 | CALN1 |
| 7203 | CHMP7 |
| 7204 | PCSK6 |
| 7205 | ATXN7L1 |
| 7206 | PACC1 |
| 7207 | SLBP |
| 7208 | COL6A5 |
| 7209 | IRX5 |
| 7210 | LOC107075317 |
| 7211 | ERC2 |
| 7212 | NOMO3 |
| 7213 | HYAL3 |
| 7214 | SEC14L3 |
| 7215 | DYNLT5 |
| 7216 | MT1E |
| 7217 | CXCL14 |
| 7218 | LOC105372310 |
| 7219 | SBNO1 |
| 7220 | MAP3K13 |
| 7221 | HEBP1 |
| 7222 | RTL1 |
| 7223 | SNRNP48 |
| 7224 | NTNG2 |
| 7225 | NEUROG3 |
| 7226 | ANO2 |
| 7227 | ADAM22 |
| 7228 | STX10 |
| 7229 | CBR4 |
| 7230 | TMEM223 |
| 7231 | CD300LF |
| 7232 | CDR1 |
| 7233 | WDR83 |
| 7234 | LRCH3 |
| 7235 | LINC00665 |
| 7236 | MPST |
| 7237 | POLR1F |
| 7238 | SPATA6 |
| 7239 | ZP4 |
| 7240 | MRPS30 |
| 7241 | VCX |
| 7242 | COLEC11 |
| 7243 | NAT14 |
| 7244 | TMEM30B |
| 7245 | OSBP |
| 7246 | TEX38 |
| 7247 | LCP1 |
| 7248 | SH3BP2 |
| 7249 | KALRN |
| 7250 | ST8SIA4 |
| 7251 | FMR1-AS1 |
| 7252 | CHRFAM7A |
| 7253 | SRR |
| 7254 | ST6GAL1 |
| 7255 | ENTPD2 |
| 7256 | PLA2G3 |
| 7257 | ENTPD5 |
| 7258 | CUL9 |
| 7259 | SEMA5B |
| 7260 | FGF17 |
| 7261 | STBD1 |
| 7262 | RHOU |
| 7263 | TMEM185A |
| 7264 | POFUT2 |
| 7265 | TSPAN16 |
| 7266 | IPO13 |
| 7267 | BRINP3 |
| 7268 | ISYNA1 |
| 7269 | SHOC1 |
| 7270 | DIRC1 |
| 7271 | HCG25 |
| 7272 | ALG9 |
| 7273 | KANSL3 |
| 7274 | ARFGEF1 |
| 7275 | BICD1 |
| 7276 | PWWP2B |
| 7277 | HTR4 |
| 7278 | KXD1 |
| 7279 | IMPA2 |
| 7280 | FRYL |
| 7281 | ANKRD54 |
| 7282 | ZNF444 |
| 7283 | APH1B |
| 7284 | AKR1C4 |
| 7285 | OR56A5 |
| 7286 | PIP4K2A |
| 7287 | PMVK |
| 7288 | LY96 |
| 7289 | ECI2 |
| 7290 | RNR3 |
| 7291 | PANK3 |
| 7292 | LMTK2 |
| 7293 | ZFYVE16 |
| 7294 | EFR3A |
| 7295 | TBCA |
| 7296 | SPICE1 |
| 7297 | SNORD47 |
| 7298 | TMED7 |
| 7299 | CDX1 |
| 7300 | MIR630 |
| 7301 | EPHB3 |
| 7302 | NSUN4 |
| 7303 | GABPB1 |
| 7304 | CDC14C |
| 7305 | PMM1 |
| 7306 | RER1 |
| 7307 | CTAG1B |
| 7308 | SDC2 |
| 7309 | DGKG |
| 7310 | THTPA |
| 7311 | ZNF622 |
| 7312 | TUNAR |
| 7313 | CGN |
| 7314 | ELK4 |
| 7315 | SYT5 |
| 7316 | EID2 |
| 7317 | EIF1AD |
| 7318 | FRMD8 |
| 7319 | INSL3 |
| 7320 | KLHDC2 |
| 7321 | BLOC1S4 |
| 7322 | PERCC1 |
| 7323 | UBTD2 |
| 7324 | UAP1L1 |
| 7325 | ALKBH6 |
| 7326 | LINC00240 |
| 7327 | SNORD20 |
| 7328 | KLK15 |
| 7329 | TWF2 |
| 7330 | PKD1L1 |
| 7331 | SEPTIN10 |
| 7332 | ICE2 |
| 7333 | EDARADD |
| 7334 | SYT12 |
| 7335 | FHOD3 |
| 7336 | IFT57 |
| 7337 | SVEP1 |
| 7338 | PDK2 |
| 7339 | NLGN2 |
| 7340 | WIPF3 |
| 7341 | GNB4 |
| 7342 | IQSEC3 |
| 7343 | ARHGAP5 |
| 7344 | NT5DC1 |
| 7345 | TMEM53 |
| 7346 | TRPV6 |
| 7347 | PCDH10 |
| 7348 | DUSP3 |
| 7349 | ANKRD13B |
| 7350 | FAM72A |
| 7351 | UBA52P6 |
| 7352 | PANX3 |
| 7353 | MS2 |
| 7354 | MS3 |
| 7355 | MS4 |
| 7356 | PDE8B |
| 7357 | DGKD |
| 7358 | LETMD1 |
| 7359 | RNR5 |
| 7360 | SCG5 |
| 7361 | CAMK2B |
| 7362 | OSCP1 |
| 7363 | SHANK2 |
| 7364 | HRK |
| 7365 | CLDN23 |
| 7366 | SLC35B1 |
| 7367 | ZFP30 |
| 7368 | NPEPL1 |
| 7369 | C19orf33 |
| 7370 | HAFML |
| 7371 | GGTLC4P |
| 7372 | AZF1 |
| 7373 | SLC17A7 |
| 7374 | TMEM129 |
| 7375 | PLXNA3 |
| 7376 | EPS8L1 |
| 7377 | FGD6 |
| 7378 | GOT1L1 |
| 7379 | ZNHIT2 |
| 7380 | CASKIN2 |
| 7381 | PRAG1 |
| 7382 | KISS1R |
| 7383 | PPM1E |
| 7384 | MRPS5 |
| 7385 | SLC16A13 |
| 7386 | REX1BD |
| 7387 | RLIM |
| 7388 | BMP3 |
| 7389 | ZNF850 |
| 7390 | HEATR5A |
| 7391 | VCPKMT |
| 7392 | MIR200CHG |
| 7393 | ZSCAN2 |
| 7394 | SNORD41 |
| 7395 | MNT |
| 7396 | LINC00844 |
| 7397 | SLC38A1 |
| 7398 | SEMA5A |
| 7399 | UPB1 |
| 7400 | SNRPC |
| 7401 | ANKMY1 |
| 7402 | MIR4640 |
| 7403 | LOC129992304 |
| 7404 | TNNI3K |
| 7405 | SPDEF |
| 7406 | CA13 |
| 7407 | STEAP1 |
| 7408 | NEUROG2 |
| 7409 | TRIM14 |
| 7410 | CSTF2T |
| 7411 | NRP2 |
| 7412 | KIF16B |
| 7413 | CEP85 |
| 7414 | RBM47 |
| 7415 | ACAD10 |
| 7416 | PET100 |
| 7417 | TRPV2 |
| 7418 | ARHGEF37 |
| 7419 | FAM98C |
| 7420 | GPR78 |
| 7421 | COX6B2 |
| 7422 | SCART1 |
| 7423 | FMNL1-DT |
| 7424 | LINC00504 |
| 7425 | MSC-AS1 |
| 7426 | FAM86B3P |
| 7427 | MRPL14 |
| 7428 | PRKD2 |
| 7429 | SMYD1 |
| 7430 | PLSCR1 |
| 7431 | NELFCD |
| 7432 | GCC2 |
| 7433 | TAMALIN |
| 7434 | YPEL3 |
| 7435 | ELOVL3 |
| 7436 | CRYZL1 |
| 7437 | NPTX2 |
| 7438 | DGKA |
| 7439 | CACFD1 |
| 7440 | LGI4 |
| 7441 | N4BP3 |
| 7442 | SNORA63 |
| 7443 | TCHP |
| 7444 | QKI |
| 7445 | DENND4A |
| 7446 | KIFAP3 |
| 7447 | SNX27 |
| 7448 | GPR101 |
| 7449 | LOC130009913 |
| 7450 | THAP11 |
| 7451 | CEP192 |
| 7452 | HCLS1 |
| 7453 | PLCB2 |
| 7454 | ZFYVE9 |
| 7455 | TERF1P3 |
| 7456 | GNB5 |
| 7457 | PLEKHH2 |
| 7458 | COL20A1 |
| 7459 | UBE2Q1 |
| 7460 | KLF7 |
| 7461 | NEU4 |
| 7462 | SRGAP3 |
| 7463 | PCDHGC4 |
| 7464 | PLAAT5 |
| 7465 | TM2D2 |
| 7466 | RBM14-RBM4 |
| 7467 | PAPPA2 |
| 7468 | HOOK1 |
| 7469 | MIR3944 |
| 7470 | NDOR1 |
| 7471 | SLC9A8 |
| 7472 | RAB8B |
| 7473 | LOC126862737 |
| 7474 | ADGRL2 |
| 7475 | METTL8 |
| 7476 | NCK2 |
| 7477 | SSX2 |
| 7478 | S1PR4 |
| 7479 | WDFY1 |
| 7480 | SLC31A2 |
| 7481 | CHMP3 |
| 7482 | CCDC9 |
| 7483 | APH1A |
| 7484 | DPP7 |
| 7485 | GLS2 |
| 7486 | EMC2 |
| 7487 | MEMO1 |
| 7488 | RBM33 |
| 7489 | AAK1 |
| 7490 | DNAI7 |
| 7491 | PTPN4 |
| 7492 | TENM4 |
| 7493 | SESN3 |
| 7494 | RGL2 |
| 7495 | OSBPL8 |
| 7496 | ITSN2 |
| 7497 | HEXD |
| 7498 | UBE2Q2P16 |
| 7499 | LOC102724428 |
| 7500 | LOC130057475 |
| 7501 | LHX2 |
| 7502 | TBCC |
| 7503 | NBPF8 |
| 7504 | LACRT |
| 7505 | MIR4435-2HG |
| 7506 | ZMAT4 |
| 7507 | HEPHL1 |
| 7508 | DNAJC2 |
| 7509 | CHRNA1 |
| 7510 | SSH1 |
| 7511 | MPHOSPH8 |
| 7512 | B3GAT3 |
| 7513 | GDAP1L1 |
| 7514 | DGKQ |
| 7515 | PARK16 |
| 7516 | RGMA |
| 7517 | FUCA2 |
| 7518 | WFDC2 |
| 7519 | OR1E2 |
| 7520 | TLE1 |
| 7521 | ZGPAT |
| 7522 | THOC3 |
| 7523 | MIR522 |
| 7524 | MARK1 |
| 7525 | ZDHHC13 |
| 7526 | DSN1 |
| 7527 | EHF |
| 7528 | ZNF528-AS1 |
| 7529 | MIR1226 |
| 7530 | NELFB |
| 7531 | B4GALT3 |
| 7532 | SYPL2 |
| 7533 | PGAP6 |
| 7534 | ZNF771 |
| 7535 | CDS2 |
| 7536 | GGTLC1 |
| 7537 | SMOC1 |
| 7538 | RPH3A |
| 7539 | ADPGK |
| 7540 | IPO9 |
| 7541 | ZDHHC17 |
| 7542 | DAB2 |
| 7543 | PNMA1 |
| 7544 | RNA18SN5 |
| 7545 | RNA28SN5 |
| 7546 | ZDHHC15 |
| 7547 | MGRN1 |
| 7548 | TMEM79 |
| 7549 | UBR7 |
| 7550 | HS6ST1 |
| 7551 | LUM |
| 7552 | EMC4 |
| 7553 | BCAR4 |
| 7554 | APOOL |
| 7555 | TMEM9 |
| 7556 | SCG2 |
| 7557 | SYNE3 |
| 7558 | PPP2R3B |
| 7559 | ANO6 |
| 7560 | SYPL1 |
| 7561 | MTERF4 |
| 7562 | CBARP |
| 7563 | ZNF280A |
| 7564 | VPS26C |
| 7565 | PSD3 |
| 7566 | ZC3H11B |
| 7567 | AK4 |
| 7568 | SLC12A8 |
| 7569 | MRAS |
| 7570 | MCF2L2 |
| 7571 | SOX21 |
| 7572 | MFSD10 |
| 7573 | RAB39B |
| 7574 | DPH7 |
| 7575 | SERINC2 |
| 7576 | RNA5-8SN1 |
| 7577 | RNA5-8SN2 |
| 7578 | RNA5-8SN3 |
| 7579 | RNA5-8SP10 |
| 7580 | RNA28SN2 |
| 7581 | RNA45SN1 |
| 7582 | RNA45SN2 |
| 7583 | RNA45SN3 |
| 7584 | RNR2 |
| 7585 | RNA18SN2 |
| 7586 | RNA18SN3 |
| 7587 | RNA28SN1 |
| 7588 | RNA28SN3 |
| 7589 | RNA5-8SN5 |
| 7590 | RNA28S5 |
| 7591 | RNA5-8SP8 |
| 7592 | RNA45SN5 |
| 7593 | RNA18S1 |
| 7594 | RNA18S2 |
| 7595 | RNA18S3 |
| 7596 | RNA18S4 |
| 7597 | RNA18S5 |
| 7598 | RNA18SN4 |
| 7599 | RNA18SP3 |
| 7600 | RNA18SP4 |
| 7601 | RNA18SP5 |
| 7602 | RNA28S1 |
| 7603 | RNA28S2 |
| 7604 | RNA28S3 |
| 7605 | RNA28S4 |
| 7606 | RNA28SN4 |
| 7607 | RNA45S1 |
| 7608 | RNA45S2 |
| 7609 | RNA45S3 |
| 7610 | RNA45S4 |
| 7611 | RNA45S5 |
| 7612 | RNA45SN4 |
| 7613 | RNA5-8S1 |
| 7614 | RNA5-8S2 |
| 7615 | RNA5-8S3 |
| 7616 | RNA5-8S4 |
| 7617 | RNA5-8S5 |
| 7618 | RNA5-8SN4 |
| 7619 | RNA5-8SP9 |
| 7620 | NOMO2 |
| 7621 | DSCAM-AS1 |
| 7622 | CLDN3 |
| 7623 | ANKRD50 |
| 7624 | LINC00310 |
| 7625 | SLC44A5 |
| 7626 | SLC1A7 |
| 7627 | AP5M1 |
| 7628 | CZ1P-ASNS |
| 7629 | PPIL3 |
| 7630 | MIR1825 |
| 7631 | GGTLC3 |
| 7632 | PTP4A3 |
| 7633 | SCN7A |
| 7634 | CAPN9 |
| 7635 | BATF2 |
| 7636 | ELP6 |
| 7637 | SH3BP1 |
| 7638 | NUTF2 |
| 7639 | KRBOX4 |
| 7640 | JAM2 |
| 7641 | DPH3 |
| 7642 | TEPSIN |
| 7643 | C14orf178 |
| 7644 | ZCCHC17 |
| 7645 | ARC |
| 7646 | POC1A |
| 7647 | TBX10 |
| 7648 | SDAD1 |
| 7649 | EFR3B |
| 7650 | ZDHHC21 |
| 7651 | PELATON |
| 7652 | ANKRD24 |
| 7653 | DMRT3 |
| 7654 | PGAM4 |
| 7655 | LASP1 |
| 7656 | MOB4 |
| 7657 | PSD4 |
| 7658 | KCNK18 |
| 7659 | YAE1 |
| 7660 | ARMC2 |
| 7661 | FIRRE |
| 7662 | PLA2G2D |
| 7663 | MRPL22 |
| 7664 | ATP11B |
| 7665 | DIPK1C |
| 7666 | IL17RD |
| 7667 | TMT1B |
| 7668 | LOC108663985 |
| 7669 | CD200R1 |
| 7670 | PKD1L2 |
| 7671 | LOC125177489 |
| 7672 | TMX4 |
| 7673 | MRAP2 |
| 7674 | METAP1 |
| 7675 | IFT70B |
| 7676 | IFT22 |
| 7677 | MIR518B |
| 7678 | IMPA1 |
| 7679 | SEMA4C |
| 7680 | LOC108281177 |
| 7681 | MIR579 |
| 7682 | CREBL2 |
| 7683 | SLC30A4 |
| 7684 | RNU2-1 |
| 7685 | CDH10 |
| 7686 | LINC01470 |
| 7687 | LOC109610631 |
| 7688 | NLGN3 |
| 7689 | P2RX1 |
| 7690 | CACNB1 |
| 7691 | SPNS1 |
| 7692 | MCF2 |
| 7693 | HMGCS1 |
| 7694 | MIR194-2 |
| 7695 | SNORA8 |
| 7696 | SLC16A10 |
| 7697 | QPRT |
| 7698 | RABL6 |
| 7699 | LGI2 |
| 7700 | RSRC1 |
| 7701 | SYTL5 |
| 7702 | CEP83 |
| 7703 | MIR103A2 |
| 7704 | IFT43 |
| 7705 | GRM2 |
| 7706 | PAK3 |
| 7707 | MVP-DT |
| 7708 | CGREF1 |
| 7709 | RIMBP2 |
| 7710 | RABGAP1 |
| 7711 | WASHC2C |
| 7712 | PRAMEF25 |
| 7713 | ARHGEF18 |
| 7714 | CHPT1 |
| 7715 | GSX2 |
| 7716 | PRDM9 |
| 7717 | MRRF |
| 7718 | BLTP3B |
| 7719 | ASIC1 |
| 7720 | MRPL48 |
| 7721 | NLGN4X |
| 7722 | RIMS1 |
| 7723 | SLC8B1 |
| 7724 | FCF1 |
| 7725 | NT5DC3 |
| 7726 | FHIP1B |
| 7727 | FKBP1B |
| 7728 | WDR74 |
| 7729 | SPRN |
| 7730 | PGS1 |
| 7731 | MAGEC1 |
| 7732 | CEACAM1 |
| 7733 | KLRA1P |
| 7734 | LOC123002309 |
| 7735 | LOC123002310 |
| 7736 | LOC129937077 |
| 7737 | LOC129937078 |
| 7738 | TNNC2 |
| 7739 | ULK4 |
| 7740 | PKD2L2 |
| 7741 | PKD1P6-NPIPP1 |
| 7742 | SLC15A2 |
| 7743 | COL6A6 |
| 7744 | CTBS |
| 7745 | MAP7D2 |
| 7746 | NHSL1 |
| 7747 | ZNF672 |
| 7748 | SNORD42A |
| 7749 | CORO7 |
| 7750 | CCDC77 |
| 7751 | GMIP |
| 7752 | PHACTR4 |
| 7753 | CYB561 |
| 7754 | CENATAC |
| 7755 | GOLGA1 |
| 7756 | ZBTB47 |
| 7757 | NT5DC4 |
| 7758 | SOX1 |
| 7759 | TENT2 |
| 7760 | TMT1A |
| 7761 | LOC107548112 |
| 7762 | KIAA1549L |
| 7763 | MIR937 |
| 7764 | ZDHHC8 |
| 7765 | LGALS13 |
| 7766 | FAM32A |
| 7767 | TSC22D3 |
| 7768 | IFT122 |
| 7769 | TRAF3IP1 |
| 7770 | SNORD13 |
| 7771 | MALRD1 |
| 7772 | UPF3A |
| 7773 | LGALSL |
| 7774 | MIR30C2 |
| 7775 | NCEH1 |
| 7776 | ARB2A |
| 7777 | PPP1R9A |
| 7778 | ODF2 |
| 7779 | LOC108663993 |
| 7780 | GTF2A1L |
| 7781 | PCDHB8 |
| 7782 | DENND2A |
| 7783 | MIR1249 |
| 7784 | MIR943 |
| 7785 | MIR516B2 |
| 7786 | METTL16 |
| 7787 | PITPNA |
| 7788 | SNORD80 |
| 7789 | ERICH3 |
| 7790 | PLCL2 |
| 7791 | DGKK |
| 7792 | TSPAN7 |
| 7793 | TRV-CAC1-2 |
| 7794 | LRP10 |
| 7795 | SLC5A5 |
| 7796 | MCOLN3 |
| 7797 | GPR89B |
| 7798 | CASKIN1 |
| 7799 | SAMD11 |
| 7800 | CCDC28A |
| 7801 | LOC129937076 |
| 7802 | GPER1 |
| 7803 | DNAJB9 |
| 7804 | NCOA7 |
| 7805 | SERPINA10 |
| 7806 | PIP4K2B |
| 7807 | GLP2R |
| 7808 | CEP295 |
| 7809 | LRRC7 |
| 7810 | KCNC1 |
| 7811 | CDH7 |
| 7812 | LINC00520 |
| 7813 | BEX2 |
| 7814 | TTC27 |
| 7815 | GCFC2 |
| 7816 | SFI1 |
| 7817 | CDKL1 |
| 7818 | MIR520G |
| 7819 | MIR3178 |
| 7820 | LPCAT3 |
| 7821 | AMMECR1 |
| 7822 | ARMC12 |
| 7823 | MIS12 |
| 7824 | CDH8 |
| 7825 | PDIA5 |
| 7826 | MARVELD3 |
| 7827 | NCS1 |
| 7828 | MIR655 |
| 7829 | PCBP2-OT1 |
| 7830 | TSSC4 |
| 7831 | BRI3BP |
| 7832 | KCNG1 |
| 7833 | SEPTIN3 |
| 7834 | EIF2D |
| 7835 | AIDA |
| 7836 | PRKD3 |
| 7837 | KCMF1 |
| 7838 | MIR103B2 |
| 7839 | LOC130056997 |
| 7840 | DNAJC14 |
| 7841 | CORO2A |
| 7842 | CCDC43 |
| 7843 | MIR551A |
| 7844 | SNORD22 |
| 7845 | BCAN |
| 7846 | TM9SF2 |
| 7847 | C1orf174 |
| 7848 | POGLUT2 |
| 7849 | NAPEPLD |
| 7850 | S100A5 |
| 7851 | LOC130057309 |
| 7852 | IFTAP |
| 7853 | TCEANC2 |
| 7854 | ERMP1 |
| 7855 | PSG4 |
| 7856 | PLA2G2E |
| 7857 | ZNF503-AS2 |
| 7858 | COL18A1-AS1 |
| 7859 | SERF2 |
| 7860 | ST8SIA1 |
| 7861 | MIR4497 |
| 7862 | MIR378C |
| 7863 | LIN7A |
| 7864 | PPFIA3 |
| 7865 | BLTP2 |
| 7866 | SCRN3 |
| 7867 | IPMK |
| 7868 | RUFY3 |
| 7869 | SPRR1B |
| 7870 | NRAD1 |
| 7871 | LINC00511 |
| 7872 | LOC129391296 |
| 7873 | PREPL |
| 7874 | MYBL1 |
| 7875 | PLD4 |
| 7876 | RLN2 |
| 7877 | CDC42EP4 |
| 7878 | HYCC2 |
| 7879 | CCDC54 |
| 7880 | MRGPRE |
| 7881 | LINC01088 |
| 7882 | SNORD38B |
| 7883 | EGFL6 |
| 7884 | LELP1 |
| 7885 | CYLC2 |
| 7886 | GJA9-MYCBP |
| 7887 | LOC130065345 |
| 7888 | CDC26 |
| 7889 | MIR454 |
| 7890 | APOBEC3F |
| 7891 | HSD3BP4 |
| 7892 | CPLX2 |
| 7893 | PTDSS2 |
| 7894 | PLA2G2F |
| 7895 | PLSCR4 |
