# Supplementary material for: Isoliquiritigenin ameliorates abnormal oligodendrocyte development and behavior disorders induced by white matter injury
Source: Front Pharmacol. 2024 Sep 11;15:1473019. doi: 10.3389/fphar.2024.1473019 (PMC11423201; doi:10.3389/fphar.2024.1473019)
Supplement: Supplementary file 6 [file Table3.DOCX]

| Supplementary Table3. Shared Target | |
| --- | --- |
| **Number** | **Target** |
| 1 | MAPK14 |
| 2 | BCHE |
| 3 | ESR1 |
| 4 | CDK5R1 |
| 5 | GSTP1 |
| 6 | HCK |
| 7 | CES1 |
| 8 | PIM1 |
| 9 | PDE5A |
| 10 | CA2 |
| 11 | CDK2 |
| 12 | ESR2 |
| 13 | LTA4H |
| 14 | MAPK10 |
| 15 | CHEK1 |
| 16 | TTR |
| 17 | AURKA |
| 18 | MAOB |
| 19 | AR |
| 20 | PDPK1 |
| 21 | HSPA8 |
| 22 | EGFR |
| 23 | FGFR1 |
| 24 | BCAT2 |
| 25 | FNTA |
| 26 | CFB |
| 27 | PGR |
| 28 | AMY1A |
| 29 | AMY1B |
| 30 | AMY1C |
| 31 | BACE1 |
| 32 | ANG |
| 33 | NQO1 |
| 34 | CDK6 |
| 35 | PTPN1 |
| 36 | GSK3B |
| 37 | MIF |
| 38 | DAPK1 |
| 39 | F2 |
| 40 | MAPK8 |
| 41 | AKR1B1 |
| 42 | DHFR |
| 43 | HSP90AA1 |
| 44 | CCNA2 |
| 45 | MTHFD1 |
| 46 | CHIT1 |
| 47 | PNP |
| 48 | IMPA1 |
| 49 | HSD17B1 |
| 50 | PDE4B |
| 51 | MMP8 |
| 52 | PRKACA |
| 53 | KDR |
| 54 | KIF11 |
| 55 | MTAP |
| 56 | AMD1 |
| 57 | SHBG |
| 58 | SRC |
| 59 | PYGL |
| 60 | PLK1 |
| 61 | NOS3 |
| 62 | PDE4D |
| 63 | CSNK1G2 |
| 64 | UCK2 |
| 65 | PCK1 |
| 66 | HSD11B1 |
| 67 | PDE3B |
| 68 | ANXA5 |
| 69 | ALDH2 |
| 70 | PPARG |
| 71 | SOD2 |
| 72 | ALB |
| 73 | ESRRG |
| 74 | AHCY |
| 75 | YARS1 |
| 76 | ISG20 |
| 77 | ADAM17 |
| 78 | F7 |
| 79 | PARP1 |
| 80 | EPHX2 |
| 81 | DPP4 |
| 82 | CBR1 |
| 83 | IMPDH2 |
| 84 | LGALS7 |
| 85 | LGALS7B |
| 86 | CDA |
| 87 | AMY2A |
| 88 | NQO2 |
| 89 | HK1 |
| 90 | F10 |
| 91 | DCK |
| 92 | REG1A |
| 93 | PNMT |
| 94 | PDHB |
| 95 | FGFR2 |
| 96 | TNK2 |
| 97 | AKR1C3 |
| 98 | GSR |
| 99 | PAH |
| 100 | CASP3 |
| 101 | RAC2 |
| 102 | ADH5 |
| 103 | SORD |
| 104 | ABO |
| 105 | SULT2A1 |
| 106 | SYK |
| 107 | AKR1C1 |
| 108 | REN |
| 109 | RHOA |
| 110 | FHIT |
| 111 | IGF1 |
| 112 | IGF1R |
| 113 | TGFBR1 |
| 114 | HDAC8 |
| 115 | HMGCR |
| 116 | MMP7 |
| 117 | BIRC7 |
| 118 | NOS2 |
| 119 | PADI4 |
| 120 | DHODH |
| 121 | THRB |
| 122 | LCK |
| 123 | ACP3 |
| 124 | ITK |
| 125 | MMP3 |
| 126 | PLAU |
| 127 | RNASE4 |
| 128 | RNASE3 |
| 129 | MMP12 |
| 130 | TYMS |
| 131 | CCL5 |
| 132 | CYP2C9 |
| 133 | SSE1 |
| 134 | ELANE |
| 135 | EPHB4 |
| 136 | ALDOA |
| 137 | NR1H2 |
| 138 | TPI1 |
| 139 | MET |
| 140 | UMPS |
| 141 | NR3C2 |
| 142 | GSTA1 |
| 143 | ARSA |
| 144 | NR1H3 |
| 145 | ERBB4 |
| 146 | CTSS |
| 147 | B3GAT1 |
| 148 | BST1 |
| 149 | PDK2 |
| 150 | SELP |
| 151 | JAK3 |
| 152 | F11 |
| 153 | BLVRB |
| 154 | FECH |
| 155 | PAK6 |
| 156 | LGALS2 |
| 157 | ARHGAP1 |
| 158 | CD1A |
| 159 | MMP13 |
| 160 | PPARA |
| 161 | IL2 |
| 162 | DTYMK |
| 163 | THRA |
| 164 | NR1H4 |
| 165 | SULT2B1 |
| 166 | RXRA |
| 167 | FABP6 |
| 168 | TPH1 |
| 169 | XIAP |
| 170 | ADK |
| 171 | MAPKAPK2 |
| 172 | MAN1B1 |
| 173 | MMP9 |
| 174 | TK1 |
| 175 | CLK1 |
| 176 | PAPSS1 |
| 177 | SERPINA1 |
| 178 | FKBP1A |
| 179 | GPI |
| 180 | CTSK |
| 181 | JAK2 |
| 182 | CBS |
| 183 | LGALS3 |
| 184 | CTSF |
| 185 | ABL1 |
| 186 | NR1I2 |
| 187 | RAB11A |
| 188 | S100A9 |
| 189 | RAB5A |
| 190 | SULT1E1 |
| 191 | SDS |
| 192 | PLA2G2A |
| 193 | ACE |
| 194 | GSTT2B |
| 195 | ARG1 |
| 196 | PPCDC |
| 197 | APRT |
| 198 | MMP2 |
| 199 | PITPNA |
| 200 | TEK |
| 201 | OTC |
| 202 | ACADM |
| 203 | KIT |
| 204 | FABP4 |
| 205 | LYZ |
| 206 | CRABP2 |
| 207 | TGM3 |
| 208 | SETD7 |
| 209 | CTSG |
| 210 | HSPA1A |
| 211 | HSPA1B |
| 212 | IMPDH1 |
| 213 | NMNAT1 |
| 214 | HAGH |
| 215 | HRAS |
| 216 | HADH |
| 217 | SRM |
| 218 | VDR |
| 219 | NR3C1 |
| 220 | OAT |
| 221 | HEXB |
| 222 | CLEC4M |
| 223 | GART |
| 224 | HNF4G |
| 225 | EPHA2 |
| 226 | AKR1C2 |
| 227 | ZAP70 |
| 228 | TPSB2 |
| 229 | ARF4 |
| 230 | BHMT |
| 231 | MME |
| 232 | MAPK1 |
| 233 | STAT1 |
| 234 | DPEP1 |
| 235 | CDC42 |
| 236 | FDPS |
| 237 | FOLH1 |
| 238 | RARA |
| 239 | GNPDA1 |
| 240 | GALE |
| 241 | HMOX1 |
| 242 | CD209 |
| 243 | EIF4E |
| 244 | LCN2 |
| 245 | CANT1 |
| 246 | ACAT1 |
| 247 | PIK3R1 |
| 248 | HINT1 |
| 249 | SHMT1 |
| 250 | MMP16 |
| 251 | NNT |
| 252 | HNMT |
| 253 | MMP1 |
| 254 | GSTM2 |
| 255 | CMA1 |
| 256 | INSR |
| 257 | GCK |
| 258 | RARG |
| 259 | ARL5A |
| 260 | ITPKA |
| 261 | SELE |
| 262 | GSTO1 |
| 263 | Rheb |
| 264 | KAT2B |
| 265 | GP1BA |
| 266 | PPP1CC |
| 267 | TGFB2 |
| 268 | MAP2K1 |
| 269 | FKBP1B |
| 270 | GSTM1 |
| 271 | GSTA3 |
| 272 | HPRT1 |
| 273 | NT5M |
| 274 | CTSB |
| 275 | ATIC |
| 276 | ADAM33 |
| 277 | RAF1 |
| 278 | GLO1 |
| 279 | RNASE2 |
| 280 | NMNAT3 |
| 281 | G6PD |
| 282 | PKLR |
| 283 | GMPR |
| 284 | DDX39B |
| 285 | UAP1 |
| 286 | FABP3 |
| 287 | ERI1 |
| 288 | DOT1L |
| 289 | CASP1 |
| 290 | SEC14L2 |
| 291 | TRDMT1 |
| 292 | WARS1 |
| 293 | CHRNA7 |
| 294 | TERT |
| 295 | ABCG2 |
| 296 | APP |
| 297 | CYP19A1 |
| 298 | MAOA |
| 299 | F3 |
| 300 | SNCA |
| 301 | KCNA3 |
| 302 | ALOX5 |
| 303 | PTGS2 |
| 304 | ABCB1 |
| 305 | ACHE |
| 306 | TLR9 |
| 307 | HSD17B2 |
| 308 | TUBB1 |
| 309 | KCNMA1 |
| 310 | PTPRS |
| 311 | GRK6 |
| 312 | CTSL |
| 313 | NOX4 |
| 314 | XDH |
| 315 | FLT3 |
| 316 | CCNB3 |
| 317 | ABCC1 |
| 318 | CSNK2A1 |
| 319 | CFTR |
| 320 | CYP1B1 |
| 321 | TNKS2 |
| 322 | TNKS |
| 323 | ALOX12 |
| 324 | STS |
| 325 | FNTA FNTB |
| 326 | TYR |
| 327 | AHR |
| 328 | IGFBP3 |
| 329 | KDM4E |
| 330 | ALOX15 |
| 331 | CDK1 |
| 332 | CD38 |
| 333 | TOP1 |
| 334 | SLC22A12 |
| 335 | MGAM |
| 336 | ESRRB |
